# CRISPR-mediated activation of autism gene *Itgb3* restores cortical network excitability via mGluR5 signaling

**DOI:** 10.1016/j.omtn.2022.07.013

**Published:** 2022-07-20

**Authors:** Fanny Jaudon, Agnes Thalhammer, Lorena Zentilin, Lorenzo A. Cingolani

**Affiliations:** 1 Center for Synaptic Neuroscience and Technology (NSYN), Fondazione Istituto Italiano di Tecnologia (IIT), 16132 Genoa, Italy; 2 Department of Life Sciences, University of Trieste, 34127 Trieste, Italy; 3 IRCCS Ospedale Policlinico San Martino, 16132 Genoa, Italy; 4 AAV Vector Unit, International Centre for Genetic Engineering and Biotechnology (ICGEB), 34149 Trieste, Italy

**Keywords:** CRISPRa, fragile X syndrome, integrins, metabotropic glutamate receptors, multi-electrode arrays, autism, calcium imaging

## Abstract

Many mutations in autism spectrum disorder (ASD) affect a single allele, indicating a key role for gene dosage in ASD susceptibility. Recently, haplo-insufficiency of *ITGB3*, the gene encoding the extracellular matrix receptor β3 integrin, was associated with ASD. Accordingly, *Itgb3* knockout (KO) mice exhibit autism-like phenotypes. The pathophysiological mechanisms of *Itgb3* remain, however, unknown, and the potential of targeting this gene for developing ASD therapies uninvestigated. By combining molecular, biochemical, imaging, and pharmacological analyses, we establish that *Itgb3* haplo-insufficiency impairs cortical network excitability by promoting extra-synaptic over synaptic signaling of the metabotropic glutamate receptor mGluR5, which is similarly dysregulated in fragile X syndrome, the most frequent monogenic form of ASD. To assess the therapeutic potential of regulating *Itgb3* gene dosage, we implemented CRISPR activation and compared its efficacy with that of a pharmacological rescue strategy for fragile X syndrome. Correction of neuronal *Itgb3* haplo-insufficiency by CRISPR activation rebalanced network excitability as effectively as blockade of mGluR5 with the selective antagonist MPEP. Our findings reveal an unexpected functional interaction between two ASD genes, thereby validating the pathogenicity of *ITGB3* haplo-insufficiency. Further, they pave the way for exploiting CRISPR activation as gene therapy for normalizing gene dosage and network excitability in ASD.

## Graphical abstract

**Figure:**
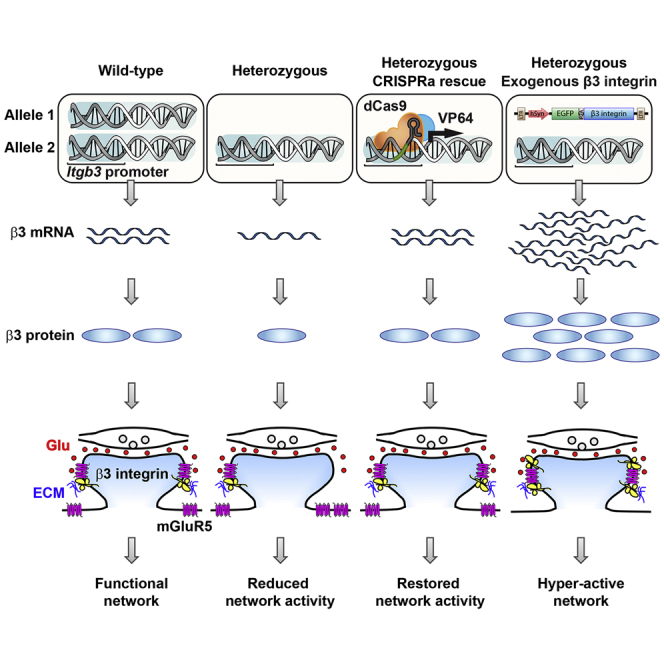

## Teaser

CRISPR activation technologies are attractive for developing therapies for autism because they provide us with a means to rebalance dysfunctional gene networks. Jaudon et al. show that enhancing expression of the autism gene *Itgb3* by CRISPR activation rebalances neuronal network activity by normalizing signaling of the metabotropic glutamate receptor mGluR5.

## Introduction

Alterations in gene dosage are important contributing factors to autism spectrum disorder (ASD), as highlighted by the high frequency of copy-number variations and mutations affecting a single allele in patients with ASD.^1^, ^2^, ^3^, ^4^, ^5^, ^6^ In particular, recent genetic screens have identified patients with ASD with rare missense and protein-truncating mutations in *ITGB3*, the gene encoding the extracellular matrix (ECM) receptor β3 integrin.^7^, ^8^, ^9^, ^10^ Together with previous genetic studies,^11^^,^^12^ these findings indicate that *ITGB3* haplo-insufficiency predisposes to ASD.^13^ Accordingly, *Itgb3* knockout (KO) mice exhibit increased grooming in novel environments and deficits in social memory,^14^ two features that recapitulate the repetitive behaviors and abnormal social interactions of patients with ASD.^15^^,^^16^

Integrins are cell adhesion molecules (CAMs) that bind to ECM proteins and counter-receptors on adjacent cells.^17^^,^^18^ Their overactivation is involved in diverse pathologies, including cancer and thrombosis, and thus they represent a target of choice for developing drug therapies based on pharmacological inhibitors.^19^ In the brain, integrins regulate synaptic connectivity and plasticity in response to chemical and mechanical cues.^13^^,^^16^^,^^20^, ^21^, ^22^, ^23^ The two most abundant neuronal integrins are β1 and β3, which have non-overlapping functions. While inhibition of β1 integrin compromises basal synaptic transmission and stabilization of long-term potentiation (LTP),^24^, ^25^, ^26^, ^27^, ^28^ full, but not partial, loss of β3 integrin impairs homeostatic plasticity, a form of plasticity that stabilizes network output in the face of external perturbations.^29^, ^30^, ^31^ Although the identification of highly selective roles for integrins in neurons suggests their potential as therapeutic targets also for brain disorders, the pathophysiological mechanisms of *Itgb3* haplo-insufficiency remain unclear and the possibility of targeting this integrin for developing ASD therapies unexplored.

To address these questions, we have established high-throughput cellular systems to assess how changes in *Itgb3* gene dosage affect synaptic ASD signaling pathways and cortical excitability, which is consistently dysfunctional in ASD cellular models.^13^^,^^32^^,^^33^ We find that *Itgb3* haplo-insufficiency impairs network excitability and synchrony by limiting synaptic signaling of the metabotropic glutamate receptor mGluR5, which is similarly disrupted in fragile X syndrome (FXS), the most frequent monogenic form of ASD.^34^, ^35^, ^36^, ^37^ To assess the therapeutic potential of targeting *Itgb3*, we have implemented neuronal CRISPR activation (CRISPRa) systems that, unlike overexpression of exogenous β3 integrin, restored precisely β3 integrin protein levels to wild-type (WT) values both *in vitro* and *in vivo*. Targeted activation of *Itgb3* rescued mGluR5 signaling and cortical excitability as effectively as pharmacological blockade of mGluR5 with the selective inverse agonist MPEP, an established FXS rescue strategy.^34^ Altogether, our findings reveal that synaptic integrins shape core ASD signaling pathways and, in turn, circuit dynamics. Further, they indicate that CRISPRa-based gene therapies are ideally suited to normalizing gene dosage and network excitability in ASD.

## Results

### *Itgb3* haplo-insufficiency impairs network excitability

**Figure 1:**
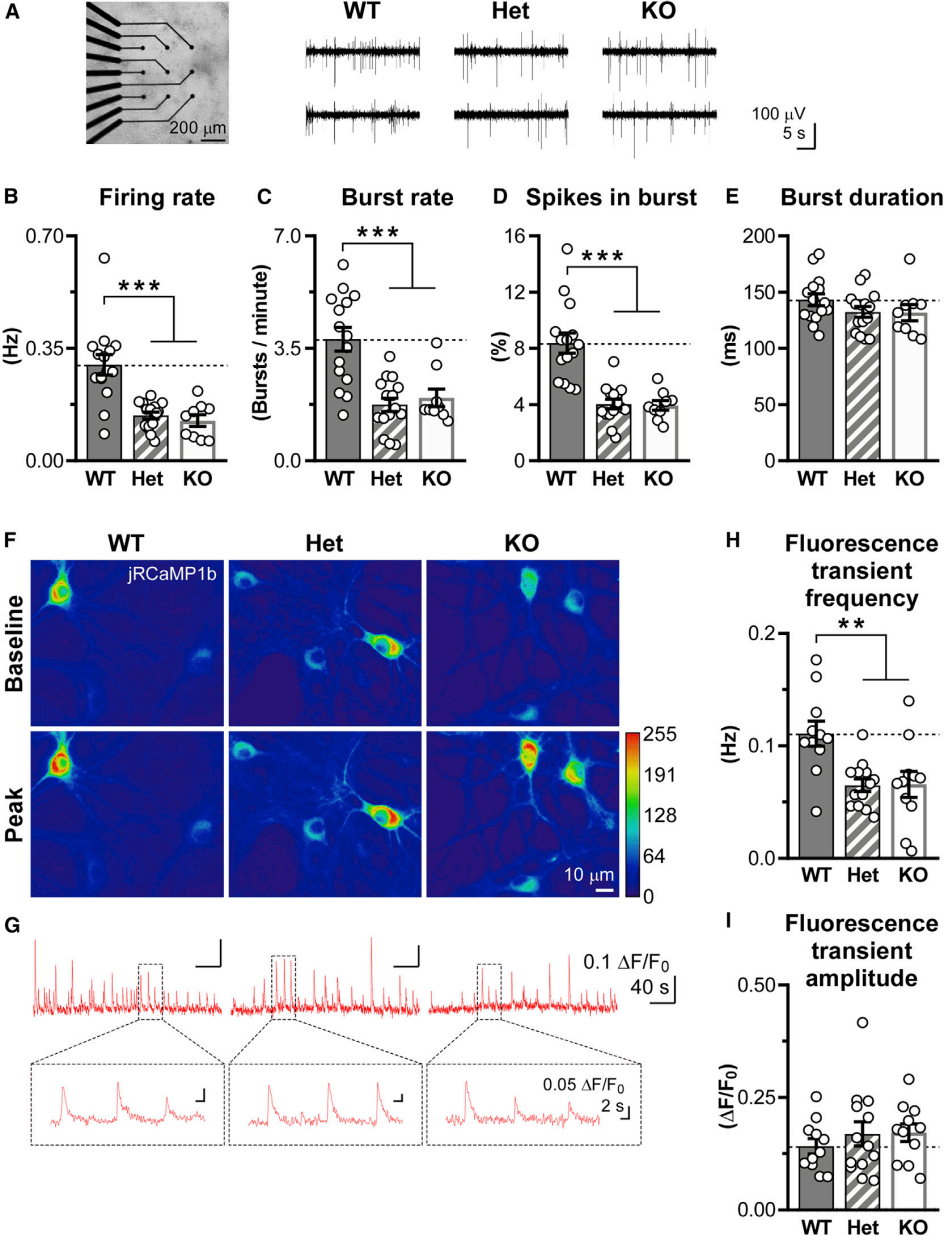
Network activity is reduced in both *Itgb3* Het and KO cortical neurons (A) Left, cortical neurons plated on MEAs. Right, representative traces from two electrodes for each genotype. (B–E) Quantification of experiments as in (A), showing that firing rate, burst rate, and percentage of spikes in burst are reduced in both *Itgb3* Het and KO cortical neurons (∗∗∗p < 0.001, one-way ANOVA followed by Tukey’s post-test, n = 9–15 recordings from 5 independent cultures). (F) Representative jRCaMP1b fluorescence transients in response to spontaneous network activity in primary cortical neurons. Images are average of 6 consecutive frames during baseline (top) and at the peak of the largest transient (bottom). (G) Spontaneous somatic jRCaMP1b responses over 5 min. Inset, higher magnification, showing good signal-to-noise ratio. (H and I) Quantification of experiments as in (F) and (G) for frequency and amplitude. The frequency of spontaneous fluorescence transients is reduced in both *Itgb3* Het and KO cortical neurons (∗∗p < 0.01, one-way ANOVA followed by Tukey’s post-test; n = 11–13 fields of view from 7–8 independent cultures). Data are presented as mean ± SEM; dots represent individual recordings. See also Figure S1.

To assess whether neuronal β3 integrin regulates network excitability, we performed multi-electrode array (MEA) recordings in WT, *Itgb3*^*+/−*^ (Heterozygous; Het), and *Itgb3*^*−/−*^ (KO) primary cortical neurons cultured in the absence of glial cells. While qRT-PCR, western blots, and confocal imaging showed that mRNA and protein levels for β3 integrin were decreased by ∼50% and ∼100% in Het and KO neurons, respectively (Figures S1A–S1D), MEA recordings indicated that firing rate, burst rate, and percentage of spikes in burst were reduced to the same extent in Het and KO networks (40%–50%; Figures 1A–1E).

To monitor network activity at the level of individual neurons, we used a red-shifted, genetically encoded Ca^2+^ indicator, jRCaMP1b,^38^ whose spontaneous somatic fluorescence transients correlate with firing activity.^39^ While the amplitude of jRCaMP1b signals was not different between the three genotypes, their frequency was decreased by ∼38% in both Het and KO neurons (Figures 1F–1I). Thus, a 50% reduction in β3 integrin expression, as observed in Het neurons, is as effective as a complete ablation of this gene in compromising network excitability.

### *Itgb3* haplo-insufficiency promotes membrane expression of mGluR5

To gain molecular insight into how β3 integrin regulates network dynamics, we screened WT, Het, and KO neurons for differences in the expression of 48 transcripts, including those for synaptic proteins, ion channels, and CAMs. We found that the expression of six genes (*Grm1*, *Grm5*, *Homer1a*, *Fmr1*, *Cacnb2*, and *Nlgn3*), all of which have been implicated in ASD (http://gene.sfari.org), was reduced in both Het and KO neurons (Figure S2A).

**Figure 2:**
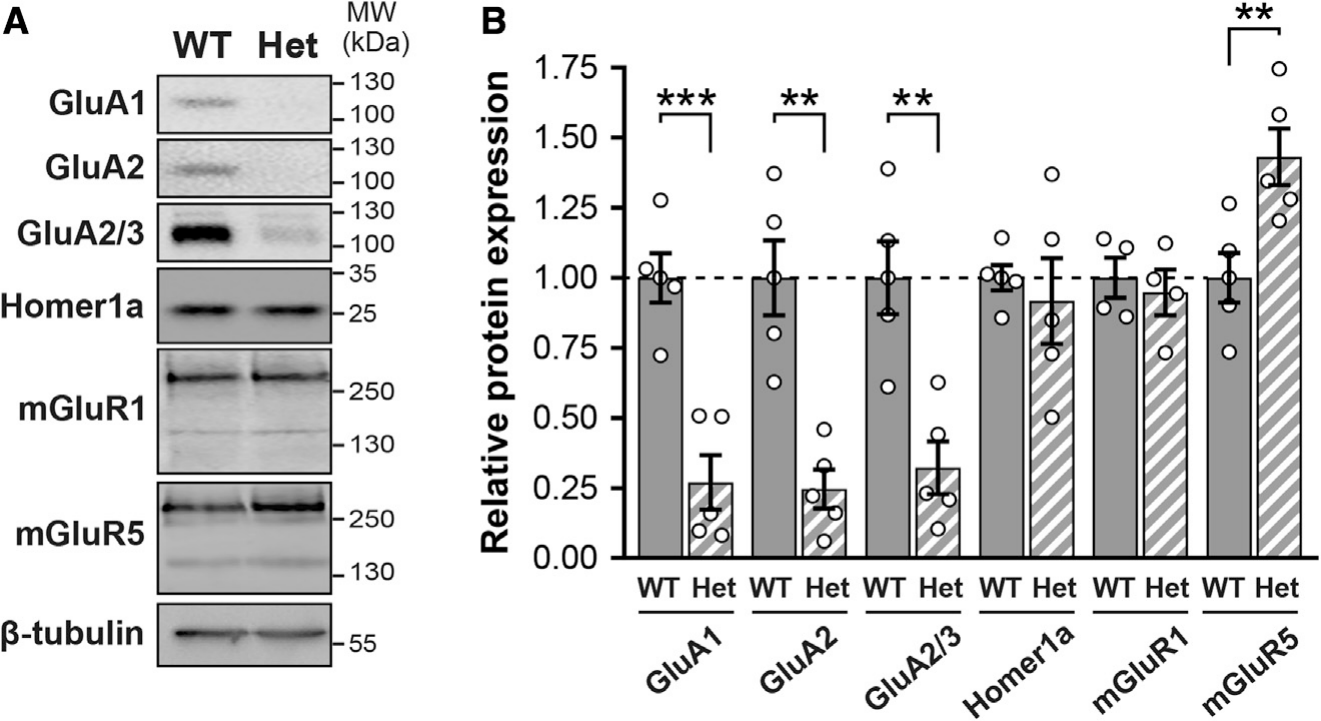
Regulation of glutamate receptor expression by β3 integrin (A) Representative western blots of membrane-enriched fractions from WT and *Itgb3* Het cortical neurons. (B) Quantification of experiments as in (A) showing that levels of AMPARs are reduced while those of mGluR5 are increased in Het neurons (∗∗p < 0.01, ∗∗∗p < 0.001, unpaired Student’s t test; n = 4–5 independent cultures). Data are shown as mean ± SEM; dots represent individual values. See also Figure S2.

Because the group I metabotropic glutamate receptors mGluR1 (*Grm1*) and mGluR5 (*Grm5*) cooperate with Homer1a to regulate excitatory synaptic transmission and intrinsic excitability,^40^, ^41^, ^42^, ^43^ we selected these three genes for further analyses. Although changes in mRNA abundance can affect protein expression, correlation between transcript and protein levels is generally low.^44^ We therefore assessed Homer1a, mGluR1, and mGluR5 protein expression in membrane fractions. Surprisingly, protein levels for Homer1a and mGluR1 were unchanged, while those for mGluR5 were 43% higher, in Het neurons. In line with previous findings,^29^^,^^45^ all AMPA-type glutamate receptors (AMPARs) were instead reduced by 68%–75% (Figure 2). Hence, *Itgb3* haplo-insufficiency promotes membrane expression of the metabotropic glutamate receptor mGluR5.

### *Itgb3* haplo-insufficiency favors functional expression of mGluR5

Several studies have investigated how integrins regulate ionotropic glutamate receptors.^25^^,^^30^^,^^45^, ^46^, ^47^, ^48^, ^49^, ^50^ By contrast, a crosstalk between integrins and mGluRs has never been described despite both being key players in shaping excitatory synaptic transmission and intrinsic excitability.^40^^,^^41^^,^^43^

**Figure 3:**
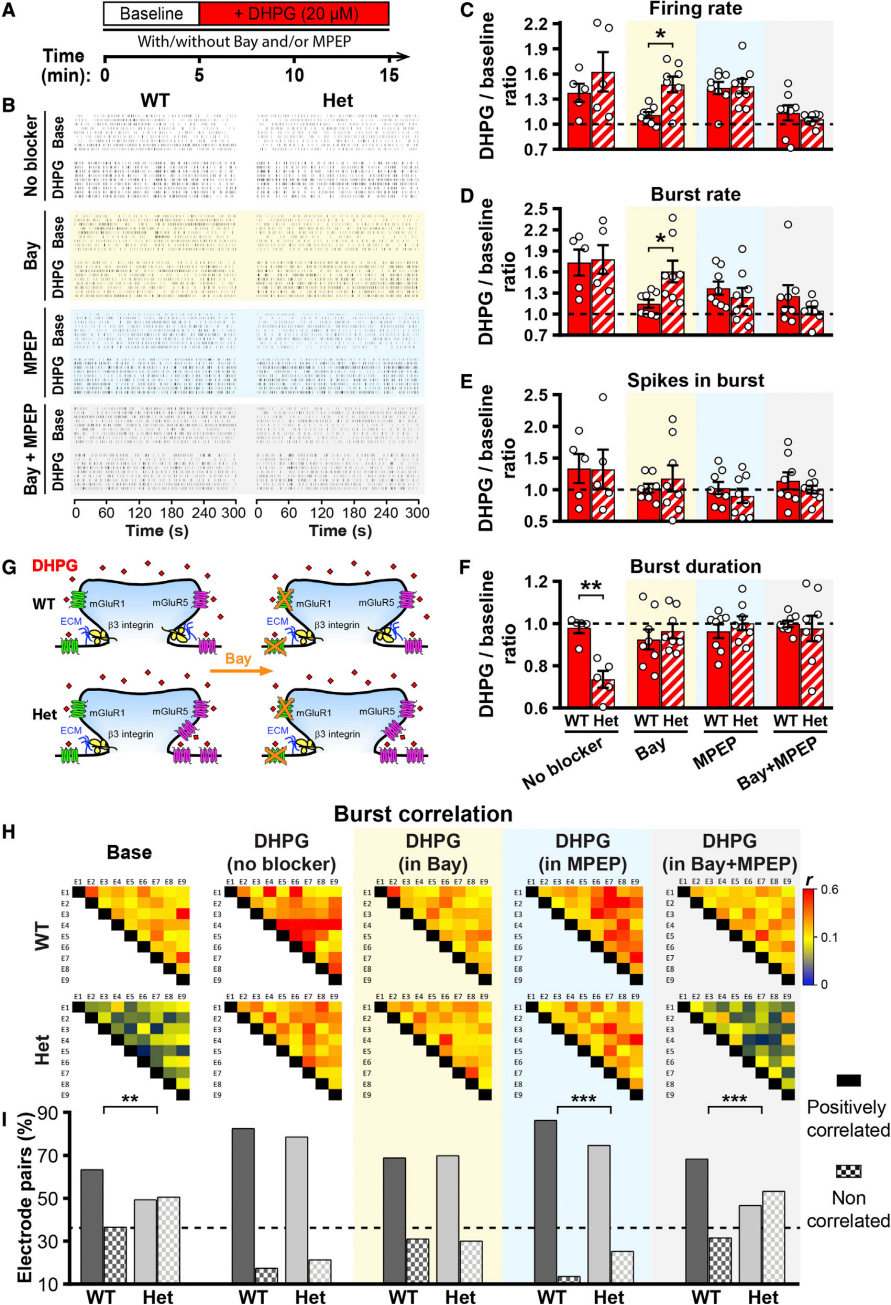
*Itgb3* haplo-insufficiency boosts overall activation of mGluR5 (A) Timeline for application of the group I mGluR agonist DHPG (20 μM) in the presence or absence of mGluR1 (Bay; 10 μM) and/or mGluR5 (MPEP; 5 μM) inverse agonists. Bay and MPEP application started 30 min before DHPG. (B) Representative raster plots of network activity from WT and Het cultures for the indicated conditions. (C–F) Quantification for experiments as in (A) and (B). Values are normalized to baseline for each recording. Selective activation of mGluR5 (Bay condition; yellow background) elevates firing and burst rate only in Het cultures (∗p < 0.05, ∗∗p < 0.01, two-way ANOVA followed by Bonferroni post-test; n = 5–8 for each condition from 5 independent cultures). Data are presented as mean ± SEM; dots represent individual values. (G) Scheme showing that DHPG activates only mGluR5 in the presence of the mGluR1 antagonist Bay. (H) Representative heatmaps of Pearson’s correlation coefficients (*r*) for burst activity from wells containing 9 electrodes (E1–E9). Selective activation of mGluR5 (DHPG in Bay condition; yellow background) rescues network synchrony of Het cultures to WT values. (I) Quantification of experiment as in (H). All electrode pairs exhibited a positive *r*. The graph shows the percentage of *r* with a p <0.05 (positively correlated) and a p >0.05 (non-correlated; ∗∗p < 0.01, ∗∗∗p < 0.001, chi-square test; n = 164–272 pairs).

To test whether *Itgb3* haplo-insufficiency favors functional expression of mGluR5, we investigated the relative contribution of mGluR1 and mGluR5 to network excitability by activating them with the selective group I mGluR agonist (S)-3,5-dihydroxyphenylglycine (DHPG) in the presence or absence of mGluR1 (Bay 36-7620; Bay) and/or mGluR5 (2-methyl-6-(phenylethynyl)-pyridine [MPEP]) antagonists. In the hippocampus, pharmacological stimulation of group I mGluRs induces synchronized discharges, mainly because activation of these receptors elevates intrinsic excitability.^40^^,^^51^^,^^52^ Accordingly, DHPG increased firing rate (≥38%; Figure 3C), burst rate (≥73%; Figure 3D), and burst synchrony (Figures 3H and 3I) in both WT and *Itgb3* Het cultures. These effects were due to activation of group I mGluRs because they were blocked by a co-application of Bay and MPEP (Figure 3). Previous work has shown that mGluR1 is more effective than mGluR5 in supporting hippocampal excitability.^53^^,^^54^ This is the case also for WT cortical networks where the mGluR1 blocker Bay, but not the mGluR5 blocker MPEP, prevented the DHPG effects as effectively as a co-application of the two blockers (Figures 3C, 3D, 3H, and 3I). These results indicate that mGluR1 is both necessary and sufficient for increasing network excitability and synchrony in WT cortical circuits.

By contrast, neither Bay nor MPEP, when applied alone, prevented the effects of DHPG in *Itgb3* Het cultures (Figures 3C, 3D, 3H, and 3I), indicating that both mGluR1 and mGluR5 are sufficient but neither one is necessary for supporting firing activity of β3-integrin-deficient neurons (Figure 3G).

### β3 integrin interacts with and regulates synaptic localization of mGluR5

**Figure 4:**
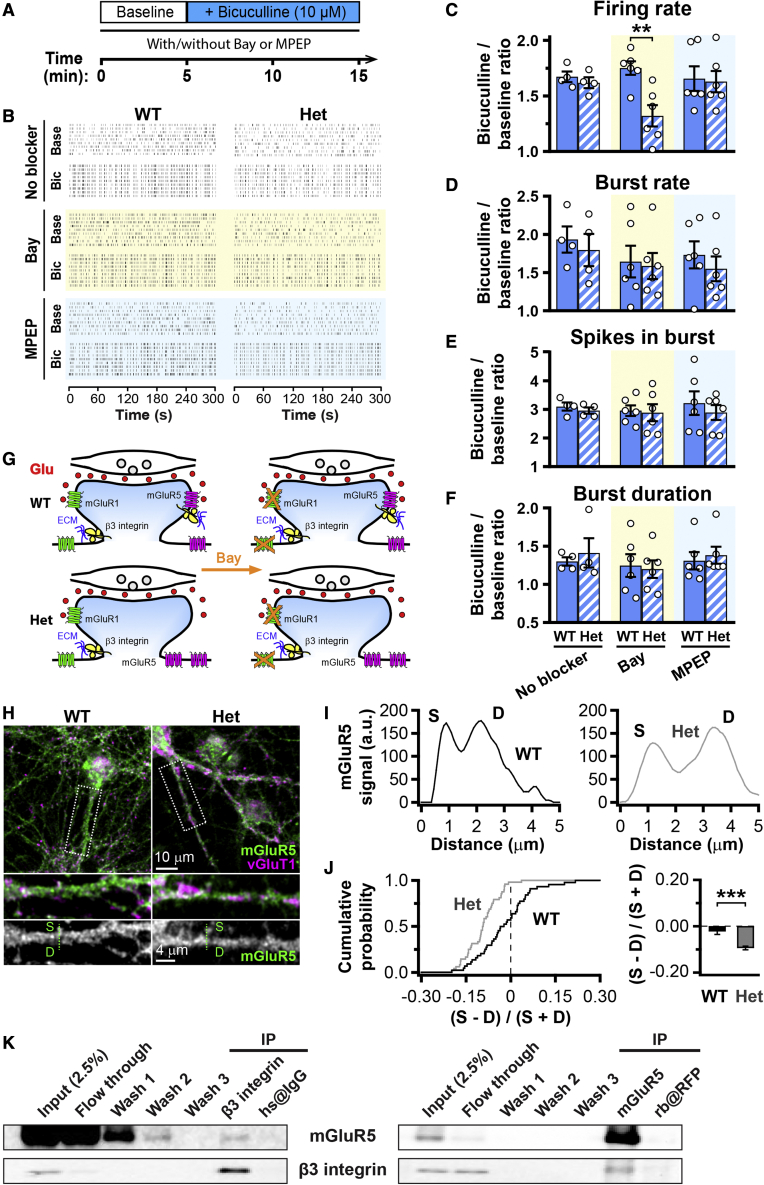
*Itgb3* haplo-insufficiency limits synaptic activation of mGluR5 (A) Timeline for bicuculline application (10 μM) in the presence or absence of mGluR1 (Bay; 10 μM) or mGluR5 (MPEP; 5 μM) inverse agonists. Bay and MPEP application started 30 min before Bicuculline. (B) Representative raster plots of network activity from WT and Het cultures for the indicated conditions. (C–F) Quantification for experiments as in (A) and (B). Values are normalized to baseline for each recording. If only mGluR5 can be activated (Bay condition; yellow background), bicuculline is less effective in elevating firing rate in Het cultures (∗∗p < 0.01, two-way ANOVA followed by Bonferroni post-test; n = 4–6 for each condition from 3 independent cultures). Data are presented as mean ± SEM; dots represent individual values. (G) Scheme showing that synaptically released glutamate (Glu) activates only synaptic mGluR5 in the presence of the mGluR1 antagonist Bay. (H) Representative confocal images of primary cortical neurons from WT and Het cultures stained for mGluR5 and the presynaptic marker vGlut1. (I) Line profiles of mGluR5 intensity for WT (left, black) and Het (right, gray) from the green dotted lines in H (S, dendritic spine; D, dendritic shaft). (J) Quantification of the distribution of mGluR5 between vGlut1-positive dendritic spines and adjacent dendritic shafts for experiments as in (H). Left, cumulative distribution of the relative difference ((S−D)/(S+D); S, synaptic signal; D, dendritic signal) for each spine/dendritic shaft pair (n = 44–48 pairs). Right, same data expressed as mean ± SEM (∗∗∗p < 0.001, unpaired two-tailed Student’s t test). mGluR5 signal is weaker in dendritic spines of *Itgb3* Het neurons. (K) Co-immunoprecipitation experiments from brain cortical extracts, showing that β3 integrin co-precipitates mGluR5 (left) and mGluR5 co-precipitates β3 integrin (right).

The reduction in burst duration induced by DHPG selectively in Het networks (Figure 3F) further indicated that group I mGluR signaling is anomalous in β3-integrin-deficient neurons. Burst duration can be affected by the level of synaptic localization of group I mGluRs.^55^^,^^56^ We therefore used the GABAergic blocker bicuculline to elevate network excitability, thus promoting activation of synaptic group I mGluRs by synaptically released glutamate.^51^^,^^55^ Bicuculline boosted network activity largely independently of group I mGluR signaling (Figures 4A–4F). In the presence of Bay, when only synaptic mGluR5 could be activated, the bicuculline-dependent increase in firing rate was nonetheless lower in *Itgb3* Het than WT cultures (Figure 4C; 32% versus 75% increase).

Interestingly, these effects mirrored those obtained upon pharmacological stimulation of mGluR5, which was effective in increasing firing rate only in Het networks (Figure 3C). This might be because β3 integrin is required for the synaptic localization of mGluR5 (Figure 4G); a requirement that cannot be compensated for by an overall increase in mGluR5 functional expression (Figures 2 and 3). To test this hypothesis, we used confocal microscopy to compare mGluR5 levels in dendritic spines, as identified by vGlut1 signal, and adjacent dendritic shafts (Figures 4H and 4I). The relative difference ((S−D)/(S+D); S, synaptic signal; D, dendritic signal), which ranges from −1 (maximal dendritic localization) to +1 (maximal synaptic localization), revealed that mGluR5 was equally distributed between dendritic spines and dendritic shafts in WT neurons but was depleted from dendritic spines of *Itgb3* Het neurons (Figure 4J). Given these results, we tested whether β3 integrin and mGluR5 were present in the same signaling complexes. Indeed, they co-immunoprecipitated from cortical tissue (Figure 4K). Taken together, these results indicate that β3 integrin interacts with and, in turn, regulates functional expression of synaptic mGluR5.

### Normalization of neuronal β3 integrin expression by CRISPRa

We next used CRISPRa to enhance transcription of β3 integrin as a means to normalize mGluR5 expression and network excitability. To this end, we employed a nuclease deficient Cas9 (dCas9) from *S. pyogenes* fused to the transcriptional activator VP64.^57^, ^58^, ^59^, ^60^, ^61^, ^62^, ^63^ We designed three gRNAs targeting the *Itgb3* promoter (Figure S3A). As quantified by qRT-PCR, all gRNAs increased β3 integrin expression by 3- to 4-fold in N2a cells, with gRNA 3 being the most effective (Figures S3B and S3C). While co-expressing gRNAs 1 and 3 had no additive effect, likely because of a steric hindrance (Figure S3A), combining gRNAs 2 and 3 enhanced β3 integrin expression by 8-fold (Figure S3C). As functional readout, we performed a cell attachment assay and found that CRISPRa strengthened adhesion of N2a cells to fibronectin, a β3 integrin ligand (Figures S3D and S3E).

We next infected primary cortical neurons with lentiviruses expressing a gRNA together with dCas9-VP64 and EGFP (Figure S4A). gRNA 3 enhanced *Itgb3* mRNA levels in WT and Het (by 2- to 2.5-fold) but not KO neurons, where both copies of the target gene are missing. As in N2a cells, combining gRNAs 2 with 3 led to larger effects in both WT and Het (3- to 4.5-fold increments; Figure S4B). Notably, exogenous expression of β3 integrin using a lentivirus with a Synapsin promoter^31^ resulted in an excessive increase in the amount of β3 integrin mRNA (∼200-fold; Figure S4B). Western blots indicated that changes in β3 integrin protein levels paralleled those of the transcript (Figures S4C and S4D). In summary, CRISPRa, but not overexpression, can be used to normalize neuronal expression of β3 integrin.

CRISPRa is not as prone as CRISPR-Cas9 to off-target effects because it does not cleave chromosomal DNA; spurious activation of off-target genes would require dCas9-VP64 to bind consistently to a promoter region.^64^^,^^65^ Indeed, previous studies have failed to identify significant off targets for CRISPRa in neurons.^59^^,^^60^ Although none of the predicted off targets for gRNAs 2 and 3 were on a promoter region (Figure S5A), we used chromatin immunoprecipitation followed by qPCR (ChIP-qPCR) to evaluate binding of dCas9 to the top ten predicted off targets for each gRNA (Figures S5B and S5C). Relative to gRNA control (Ctrl), gRNAs 2 and 3 induced a 25-fold enrichment of dCas9 at the *Itgb3* promoter (Figures S5C), confirming binding to the target site. A small enrichment of dCas9 was detected for two predicted off targets (#9 of gRNA 2 and #6 of gRNA 3; Figure S5C). As quantified by qRT-PCR, this had, however, no effect on the expression of the genes at these loci (*Msra* and *Braf*; Figure S5D).

β3 integrin is expressed in dendrites in apposition to synaptic markers.^30^^,^^45^^,^^49^ As quantified by confocal microscopy, CRISPRa rescued the reduced expression of β3 integrin in soma and dendrites of Het neurons to WT values (Figures S4E–S4G) while having no effects in KO neurons (Figure S1E). Size and density of dendritic β3 integrin clusters were largely preserved (Figures S4E and S4G). By contrast, expression of exogenous β3 integrin led to widespread and ectopic localization of this protein (Figures S4E–S4G). None of the experimental conditions affected expression of and co-localization with the synaptic marker vGlut1 (Figures S4H and S4I). In summary, β3 integrin maintains its expression pattern when the activity of its endogenous promoter is enhanced by CRISPRa.

### Rebalancing β3 integrin levels normalizes mGluR5 expression and network excitability

**Figure 5:**
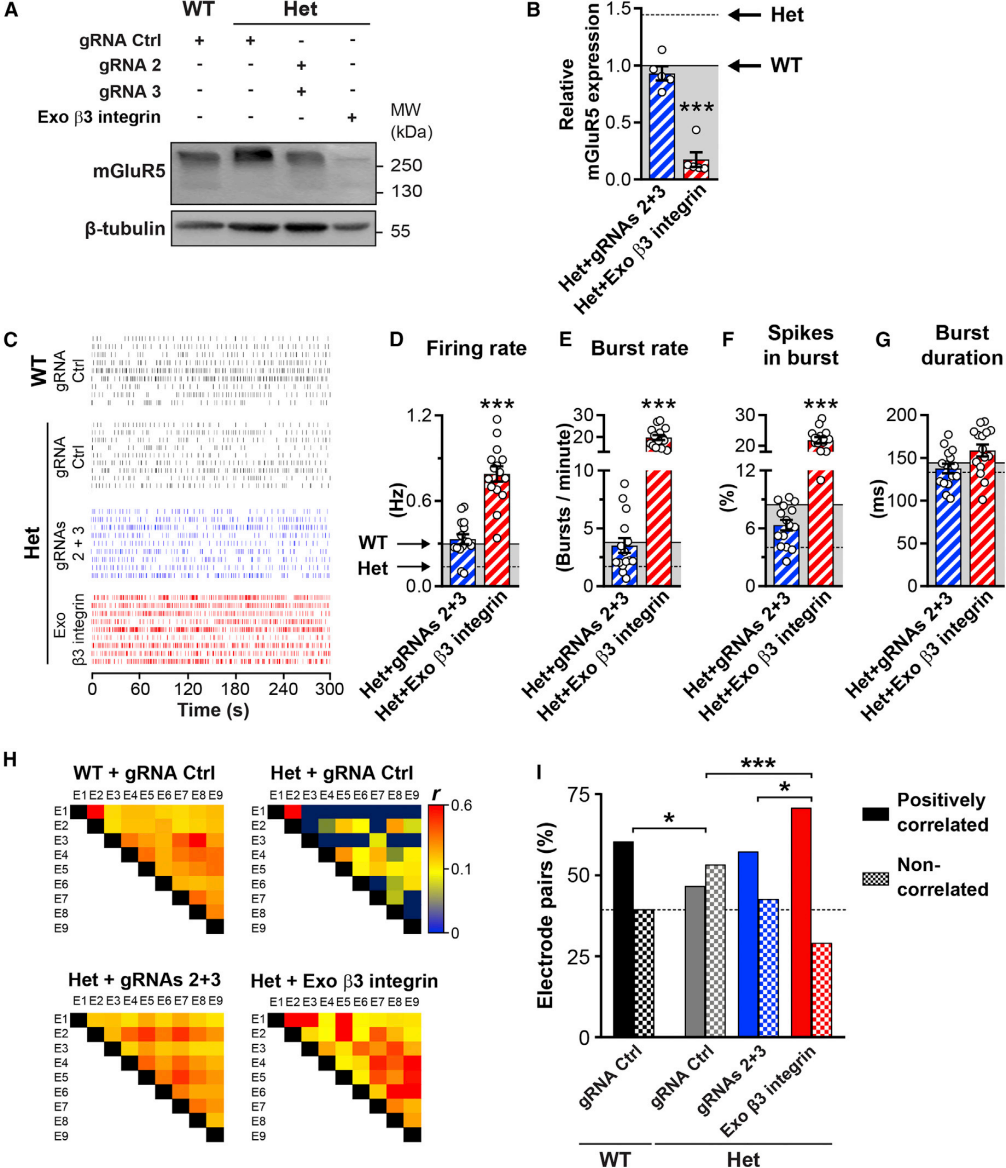
CRISPRa normalizes mGluR5 expression and firing activity in *Itgb3* Het cortical networks (A) Representative western blots of membrane-enriched fractions from WT and *Itgb3* Het cortical neurons expressing the indicated constructs. (B) Quantification of experiments as in (A) showing that CRISPRa restores mGluR5 expression levels in Het neurons to WT values (∗∗∗p < 0.001 relative to WT, one-way ANOVA followed by Tukey’s post-test; n = 5 independent cultures). (C) Representative raster plots of network activity in MEA experiments for WT and *Itgb3* Het cortical neurons expressing the indicated constructs. (D–G) Quantification of experiments as in (C). Unlike exogenous expression of β3 integrin, CRISPRa normalizes cortical network activity in Het cultures (∗∗∗p < 0.001 relative to WT, one-way ANOVA followed by Tukey’s post-test, n = 15 each from 5 independent cultures). Data are presented as mean ± SEM; dots represent individual values. (H) Representative heatmaps of Pearson’s correlation coefficients (*r*) for burst activity from wells containing 9 electrodes (E1–E9). Network synchrony correlates with β3 integrin levels. (I) Quantification of experiment as in (H). All electrode pairs exhibited a positive *r*. The graph shows the percentage of *r* with a p <0.05 (positively correlated) and a p >0.05 (non-correlated; ∗p < 0.05, ∗∗∗p < 0.001, chi-square test; n = 447–532 pairs). See also Figures S6 and S7.

We first addressed whether rebalancing β3 integrin levels with CRISPRa is effective at normalizing mGluR5 expression. In *Itgb3* Het neurons, CRISPRa brought both mRNA (Figure S2A) and protein levels (Figures 5A and 5B) of mGluR5 back to WT values. By contrast, overexpressing β3 integrin nearly abolished mGluR5 expression (Figures 5A and 5B).

We next assessed, by MEA recordings, how enhancing neuronal expression of β3 integrin regulates circuit dynamics. While CRISPRa was effective in restoring excitability and synchrony of Het neurons to WT values, exogenous expression of β3 integrin induced oversynchronous bursting (Figures 5C–5I). Importantly, CRISPRa acted specifically via *Itgb3* because it had no effect in KO neurons (Figure S6).

Since changes in inhibitory synaptic strength can contribute to differences in network dynamics, we evaluated the effects of β3 integrin in the presence of bicuculline, a GABA_A_ receptor blocker. Disinhibition promoted firing and burst rate, though to a similar extent in most experimental conditions (Figure S7; see also Figure 4). In neurons expressing exogenous β3 integrin, some increments were not as pronounced (Figure S7), possibly because of a ceiling effect. Altogether, these data suggest that β3 integrin supports network activity irrespective of synaptic inhibition.

**Figure 6:**
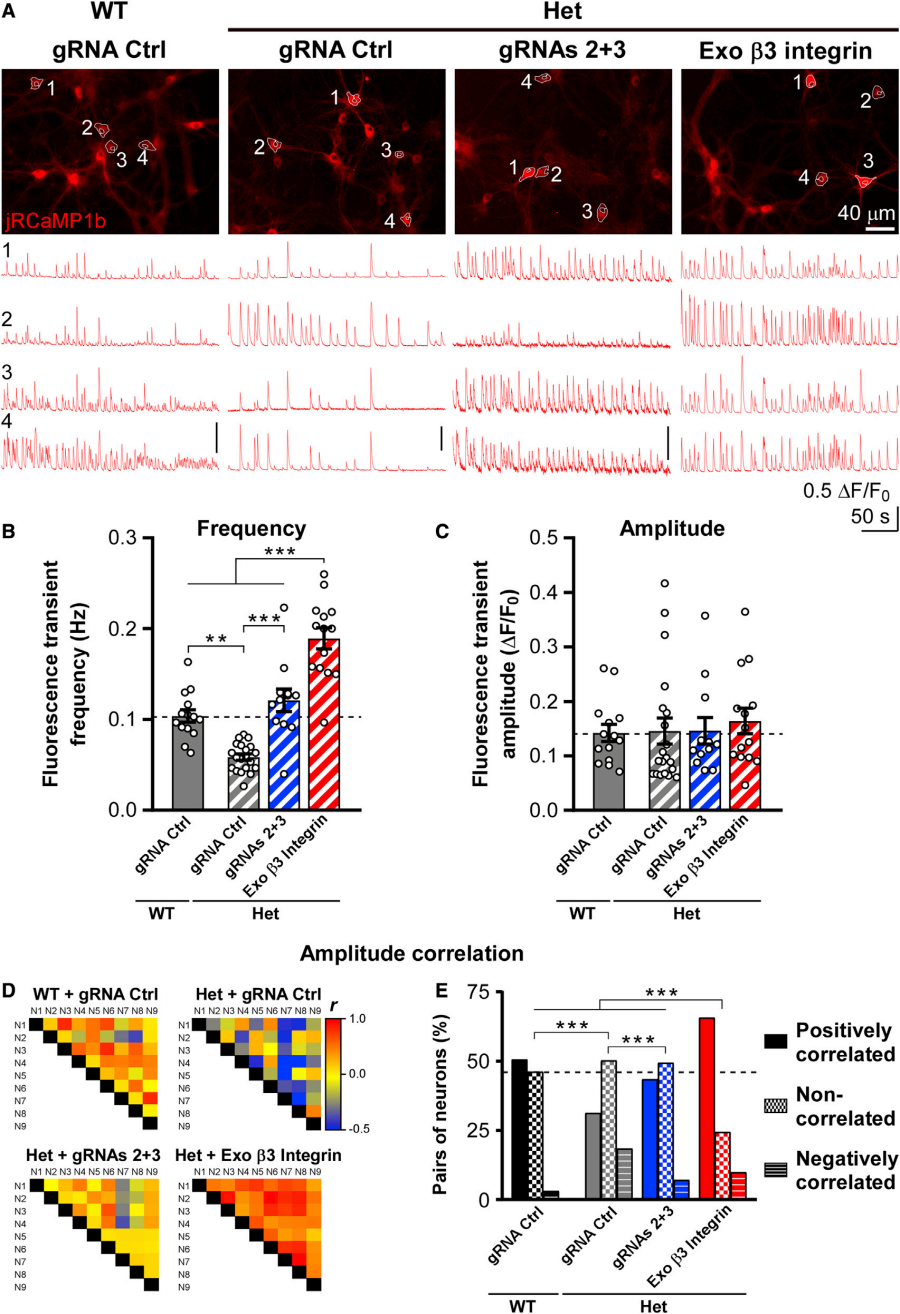
CRISPRa normalizes spontaneous activity in *Itgb3* Het neurons (A) Top, representative images of jRCaMP1b in WT and *Itgb3* Het primary cortical neurons expressing the indicated constructs. Images are an average over a 5 min recording period. Bottom, spontaneous somatic fluorescence responses from the ROIs shown in the above panels. (B and C) Quantification of experiments as in (A). CRISPRa rescues the reduction in fluorescence transient frequency of Het neurons (∗∗p < 0.01, ∗∗∗p < 0.001, one-way ANOVA followed by Tukey’s post-test, n = 12–20 fields of view from 5 independent cultures). Data are shown as mean ± SEM; dots represent individual values. (D) Heatmaps of Pearson’s correlation coefficients (*r*) for fluorescence transient amplitudes from representative fields of view. *r* correlates with β3 integrin levels. (E) Quantification of experiment as in (D). The graph shows the percentage of positive *r* with a p <0.05 (positively correlated), negative *r* with a p <0.05 (negatively correlated), and *r* with a p >0.05 (non-correlated; ∗∗∗p < 0.001, chi-square test; n = 158–396 pairs). See also Figure S8.

To monitor circuit defects and their rescue at the level of individual neurons, we resorted to jRCaMP1b. Somatic fluorescence transients exhibited differences in frequency between the various conditions analogous to those found for network firing rates (Figures 6A and 6B versus Figures 5C and 5D). Unlike MEA recordings, fluorescence signals can be interpreted also in terms of their amplitude. Mean fluorescence transients were of a similar size across conditions (Figures 6C and S8B), whereas individual transients were highly variable in amplitude within each recording (Figures 1G and 6A), suggesting variability of the underlying firing activity.^39^

Because a positive correlation in the amplitude profiles between two neurons is indicative of similar firing rates (Figure S8D), we compared the fluorescence amplitude profiles of all the neurons within a field of view. 51% and 31% of neuron pairs were positively correlated in WT and Het networks, respectively. In Het, CRISPRa could restore the percentage of positively correlated neuron pairs to WT values, while exogenous β3 integrin increased positive correlation far above WT levels (Figures 6D, 6E, S8C, and S8E). These data suggest that neuronal synchronization, which is abnormal in many models of ASD,^33^ is critically dependent on *Itgb3*.

### Chronic blockade of mGluR5 rescues impaired network function of *Itgb3* Het neurons

Our data indicate that *Itgb3* haplo-insufficiency reduces network excitability (Figure 1) and enhances functional expression of extra-synaptic mGluR5 (Figures 2, 3, and 4). Further, rebalancing β3 integrin levels with CRISPRa effectively normalizes both network activity and mGluR5 expression (Figures 5 and 6).

**Figure 7:**
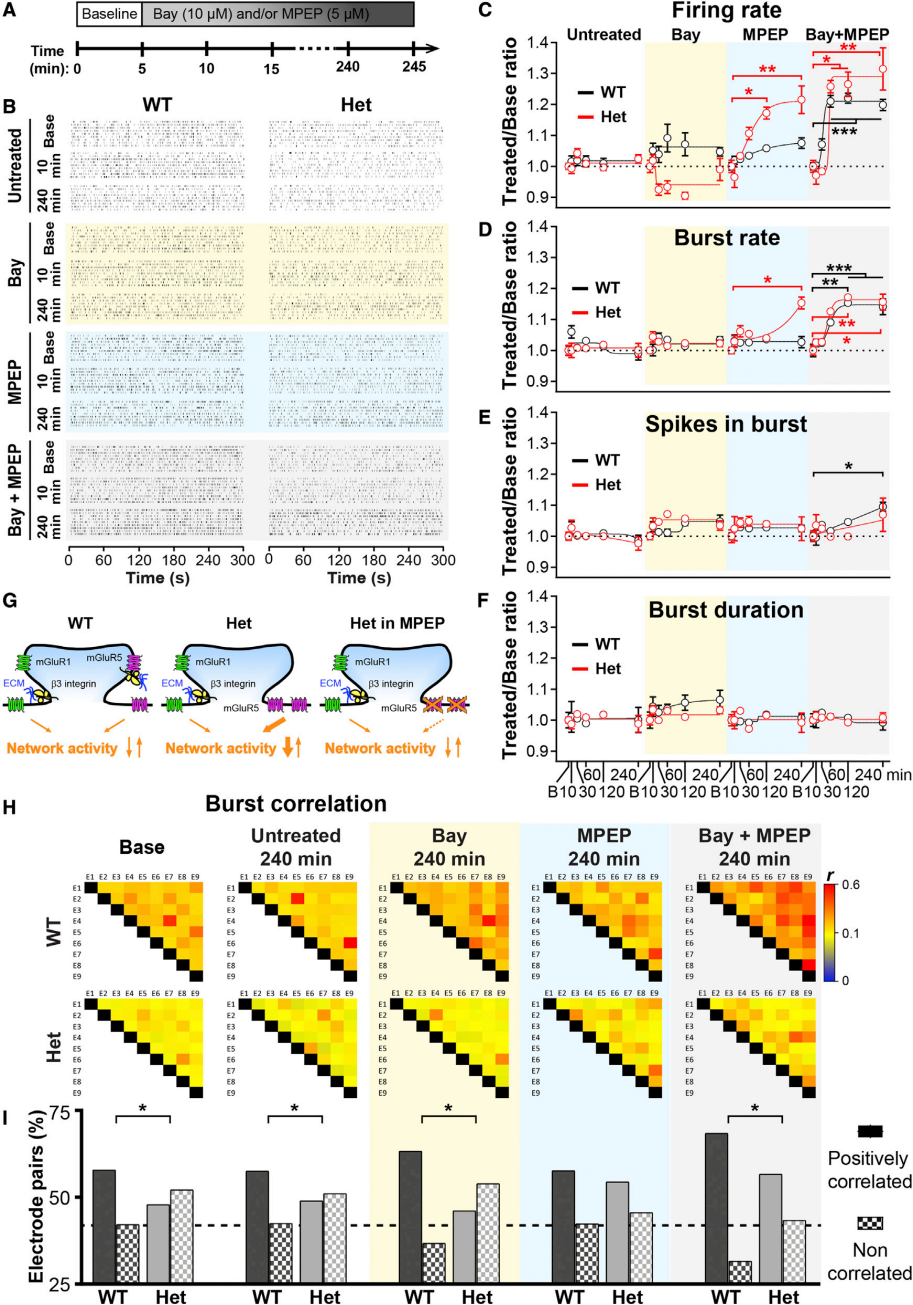
Chronic blockade of mGluR5 rescues impaired network activity of *Itgb3* Het neurons (A) Experimental timeline of MEA experiments with mGluR1 (Bay; 10 μM) and mGluR5 (MPEP; 5 μM) inverse agonists. (B) Representative raster plots of network activity from WT and *Itgb3* Het cultures for the indicated conditions. (C–F) Quantification of experiments as in (A) and (B) after 10, 30, 60, 120, and 240 min long applications of the indicated drugs. Values are normalized to baseline (B) for each recording. Chronic blockade of mGluR5 with MPEP increases firing and burst rate selectively in *Itgb3* Het cultures (∗p < 0.05, ∗∗p < 0.01, ∗∗∗p < 0.001, two-way ANOVA followed by Dunnett post-test; n = 4–8 per condition; 3 independent cultures). Data are presented as mean ± SEM. Lines through points are sigmoid fits. (G) Scheme depicting the action of the mGluR5 inverse agonist MPEP on *Itgb3* Het neurons. (H) Representative heatmaps of Pearson’s correlation coefficients (*r*) for burst activity from wells containing 9 electrodes (E1–E9). (I) Quantification of experiment as in (H). All electrode pairs exhibited a positive *r*. The graph shows the percentage of *r* with a p <0.05 (positively correlated) and a p >0.05 (non-correlated; ∗p < 0.05, chi-square test; n = 128–280 pairs). Chronic blockade of mGluR5 with MPEP rescues burst synchrony in *Itgb3* Het cultures.

To test whether it is enhanced extra-synaptic mGluR5 signaling that compromises the excitability of *Itgb3* Het neurons, we employed the inverse agonists Bay and MPEP, which block constitutive agonist-independent activity of mGluR1 and mGluR5, respectively. Chronic co-application of Bay and MPEP is known to potentiate excitatory synaptic transmission in cortical neurons.^42^ Accordingly, when used together, Bay and MPEP increased firing rate (≥20% at 1–4 h), burst rate (≥15% at 2–4 h), and network synchrony of both WT and Het neurons (Figure 7). Compellingly, chronic treatment with MPEP alone was sufficient to increase excitability (Figures 7B–7D) and synchrony (Figures 7H and 7I) of *Itgb3* Het, but not WT, circuits.

Altogether, these findings indicate that heightened mGluR5 activation in β3-integrin-deficient neurons impairs network excitability and synchrony. Further, they suggest that circuit defects caused by *Itgb3* haplo-insufficiency can be rescued by either CRISPR-mediated activation of *Itgb3* or chronic pharmacological blockade of mGluR5.

### CRISPRa normalizes β3 integrin expression *in vivo*

**Figure 8:**
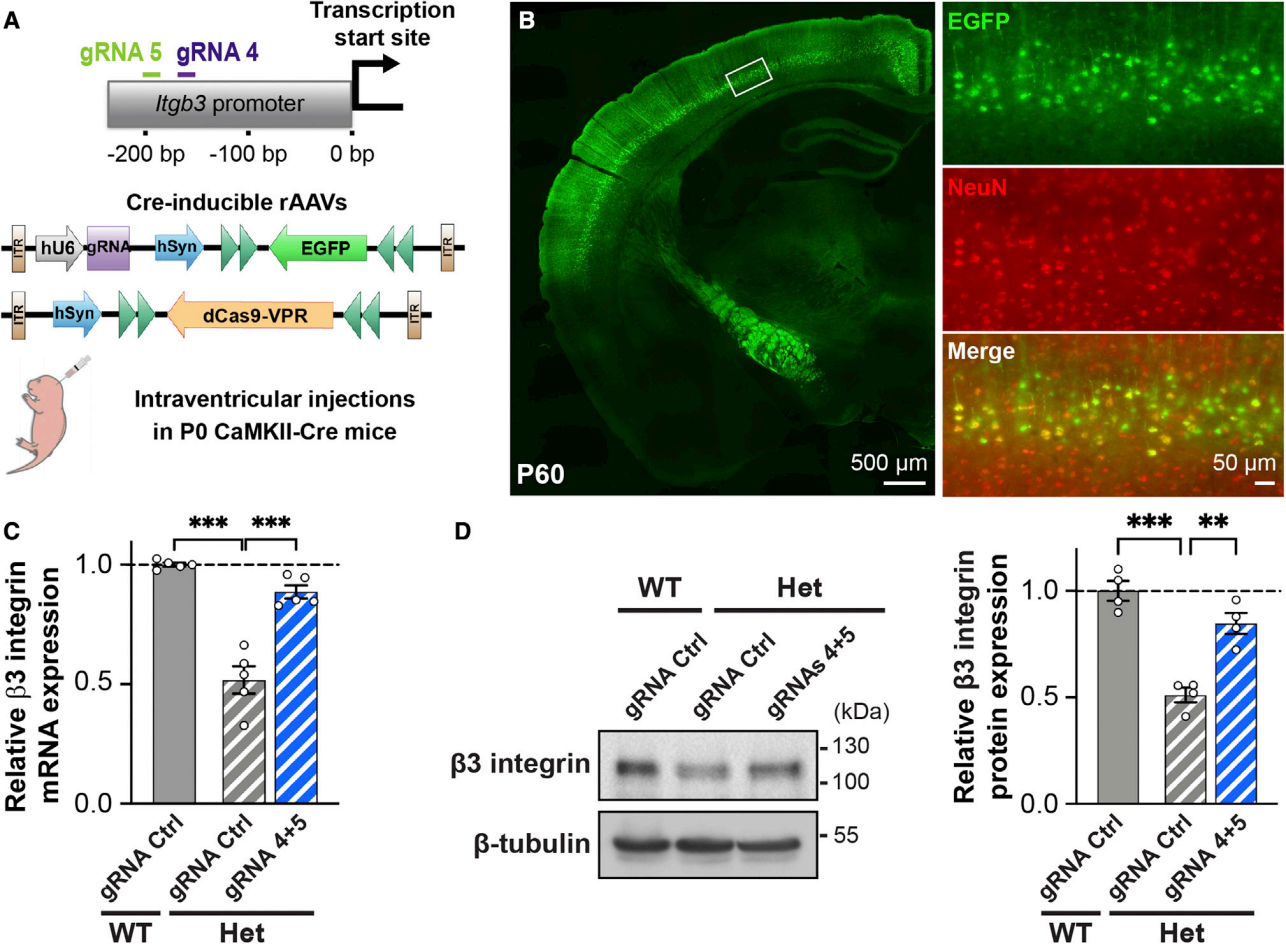
Normalization of β3 integrin expression *in vivo* by CRISPRa (A) gRNA targets on the *Itgb3* promoter and rAAV constructs for *in vivo* experiments. (B) Coronal section of injected P60 mouse showing EGFP expression in deep layer cortical pyramidal neurons. (C) qRT-PCR quantification of *Itgb3* mRNA levels in cortex of WT and *Itgb3* Het P60 mice expressing the indicated constructs (n = 5 cortices per group). (D) Left, western blots of membrane fractions from cortices of P60 mice. Right, western blot quantification (n = 4 cortices per group). CRISPRa rescues *Itgb3* gene dosage in *Itgb3* Het neurons *in vivo* at both the mRNA and protein level (∗∗p < 0.01, ∗∗∗p < 0.001, one-way ANOVA followed by Tukey’s post-test). Data are presented as mean ± SEM; dots represent individual values. See also Figure S9.

In the brain, β3 integrin is enriched in deep-layer cortical pyramidal neurons.^13^^,^^66^^,^^67^ To explore the translational potential of modulating β3 integrin expression, we therefore expressed the CRISPRa machinery *in vivo* in these neurons. To this end, we crossed the *Itgb3* mouse line with CaMKIIa-Cre knockin mice, which express the recombinase Cre in pyramidal neurons of the forebrain. Deep-layer cortical pyramidal neurons can be efficiently targeted in these mice with intraventricular injections at postnatal day 0 (P0) of Cre-dependent recombinant adeno-associated viruses (rAAVs; AAV-PHP.eB serotype; Figures 8A and 8B).

Because of the reduced packaging capacity of rAAVs, we used dCas9 from *S. aureus* (Sa-dCas9), which is smaller than the canonical dCas9 from *S. pyogenes*, while exhibiting improved specificity.^68^ We co-injected two rAAVs: the first (pAAV-Syn-DIO-Sa-dCas9-VPR) to deliver Sa-dCas9 fused to the transcriptional activator VPR, while the second (pAAV-U6-gRNA-Syn-DIO-EGFP) to deliver a gRNA (Ctrl or active) and EGFP (Figure 8A). As *S. aureus* Cas9 uses a different protospacer adjacent motif (PAM), we generated three new gRNAs targeting the *Itgb3* promoter and tested their efficiency in N2a cells (Figures S9A and S9B). From these experiments, we selected gRNAs 4 and 5, the combination of which resulted in a 7.2-fold increase in β3 integrin expression (Figure S9B).

*In vivo* CRISPRa treatment with these gRNAs restored cortical β3 integrin expression of *Itgb3* Het mice to WT values at both mRNA and protein levels (Figures 8C and 8D). To assess CRISPRa specificity *in vivo*, we performed a genome-wide ChIP-seq analysis and compared the peaks identified in the gRNA Ctrl and gRNA 4 + 5 conditions. While we observed on-target binding at the *Itgb3* promoter (Figures S9C and S9D), we did not detect any additional target significantly enriched in the gRNA 4 + 5 conditions (Figure S9E). We nevertheless checked the expression level of the four genes (*Gm9758*, *Gm11168*, *Asmt*, and *Speer4cos*) with a peak within 1 kb of their transcription start site (TSS) and found no difference in gene expression (Figure S9F), confirming the specificity of CRISPRa *in vivo*.

Taken together, these results indicate that CRISPRa can be used to normalize *Itgb3* expression in cortical pyramidal neurons *in vivo*.

## Discussion

Our study reveals an unexpected function for the ASD gene *Itgb3* in regulating synaptic signaling of mGluR5 with a direct impact on neuronal excitability. Further, it indicates CRISPR-mediated activation of *Itgb3* as suitable gene therapy to normalize network defects in ASD. First, by using MEA recordings and Ca^2+^ imaging of populations of neurons, we found that *Itgb3* haplo-insufficiency impairs activity and synchrony of cortical networks (Figure 1). Second, by combining biochemical, imaging, and pharmacological analyses, we demonstrated that reduced levels of neuronal β3 integrin favor extra-synaptic over synaptic mGluR5 signaling (Figures 2, 3, and 4). Third, we implemented CRISPRa to control *Itgb3* expression *in vitro* and *in vivo* (Figures 8 and S4). Using this approach, we established that it is necessary to restore WT gene dosage of *Itgb3* in order to rescue mGluR5 expression and neuronal excitability (Figures 5 and 6). Fourth, we determined that pharmacological blockade of mGluR5 with the selective inverse agonist MPEP rescues circuit defects of *Itgb3* Het neurons (Figure 7), thereby mechanistically linking molecular and cellular deficits of *Itgb3* haplo-insufficiency.

Multiple cues from ECM and glial cells regulate circuit dynamics. For example, enzymatic digestion of ECM components induces epileptiform activity in primary neurons.^69^, ^70^, ^71^ Integrins on the neuronal surface are ideally positioned to adjust neuronal excitability in response to changes in the extracellular environment. In particular, the glial factors TNFα and SPARC, which are down- and up-regulated by action-potential firing, increase and decrease β3 integrin expression, respectively.^30^^,^^48^^,^^72^ This integrin is therefore negatively regulated by neuronal activity via glia-released factors. Because we find here that elevating β3 integrin expression boosts, in turn, cortical excitability via mGluR5 signaling, we propose the existence of a negative feedback loop between neuronal β3 integrin and the excitability of the network.

Both MEA recordings and Ca^2+^ imaging indicated that β3 integrin promotes network synchrony (Figures 5H, 5I, 6D, and 6E). Several factors determine network dynamics, such as the number of shared connections, the excitatory/inhibitory ratio, and the level of intrinsic excitability.^41^^,^^73^^,^^74^ β3 integrin positively modulates membrane expression of AMPARs (Figure 2) and excitatory synaptic strength,^29^^,^^30^^,^^45^ though not the overall number of excitatory synaptic connections (Figure S4I). By contrast, the role of this integrin in setting the strength of inhibitory synaptic coupling is likely minor because changes in its expression had no effect on the degree of disinhibition (Figure S7) or the expression of GABA receptors (Figure S2). In addition to those for AMPARs, β3 integrin regulated other neuronal genes (Figure S2), many of which are involved in ASD. Although some of these effects could be secondary to changes in network activity, they clearly indicate that a mere 50% deficiency in one ASD gene affects the expression of many others. Five of them (*Grm1*, *Grm5*, *Homer1a*, *Fmr1*, and *Nlgn3*) drew our attention as potentially more tightly linked to integrin signaling in ASD because their expression was diminished in both *Itgb3* Het and KO neurons and could be rescued to WT values by CRISPRa (Figure S2).

A closer analysis of two of them (*Grm1 and Grm5*; mGluR1 and mGluR5) indicated that β3 integrin, which is localized at excitatory synapses (Figures S4E–S4I), interacts with mGluR5 (Figure 4K) and contributes at anchoring this group I mGluR at peri-synaptic locations (Figure 4). Group I mGluRs play a major role in the induction of both a Hebbian (mGluR-dependent long-term depression [mGluR-LTD]) and a homeostatic form of synaptic plasticity (synaptic down-scaling).^42^^,^^75^ While in mGluR-LTD mGluR1/5 are activated by synaptically released glutamate, in homeostatic synaptic down-scaling, they disperse from the synapse to enter a constitutive, agonist-independent activated state.^42^^,^^76^ The results from both the acute activation (Figures 3 and 4) and chronic blockade of mGluR1/5 (Figure 7) are in line with a model whereby *Itgb3* haplo-insufficiency favors extra-synaptic, constitutively active mGluR5 over synaptic, agonist-dependent mGluR5. This presumably reduces membrane expression of AMPARs independently of GluA1 phosphorylation (Figure 2, S2B, and S2C), excitatory synaptic strength,^30^^,^^45^ and network excitability (Figure 1). The effects of β3 integrin are unlikely mediated by Homer1a because the transcript and protein for this gene were reduced and unchanged, respectively, in *Itgb3* Het neurons (Figures 2 and S2); elevated levels of Homer1a would instead be required to support dispersed and constitutively active mGluR5.^42^^,^^77^ The function that we report here for β3 integrin at excitatory synapses appears similar to that played by other CAMs. For example, extracellular leucine-rich repeat and fibronectin type III domain-containing 1 (ELFN1) clusters and regulates the activity of mGluR7 at synapses on somatostatin interneurons.^78^^,^^79^ It is therefore likely that CAMs and extracellular interactions cooperate with intracellular scaffolding proteins, such as short and long forms of Homer, in regulating localization and function of mGluRs.^41^

Dysregulation of mGluR5 signaling is a common feature of several neurological disorders, including schizophrenia, addictive disorders, Phelan-McDermid syndrome, and FXS, the most frequent monogenic form of ASD. While many of these disorders are characterized by a general down-regulation of mGluR5 and may benefit from mGluR5-positive modulators, FXS is associated with enhanced mGluR5 activity, and, indeed, various mGluR5 inhibitors can rescue cellular, electrophysiological, and behavioral defects of the Fmr1^−/y^ mouse model of FXS.^34^, ^35^, ^36^, ^37^ The mGluR5 signaling defects that we identified in *Itgb3* Het neurons are closely reminiscent of those found in the Fmr1^−/y^ mouse model. Like in *Itgb3* Het neurons, mGluR5 signaling in Fmr1^−/y^ mice is not only potentiated but is also skewed toward an extra-synaptic activation mode.^77^^,^^80^^,^^81^ Together with our results that *Fmr1* transcript levels are correlated to those of *Itgb3* (Figure S2) and the recent finding that fragile X mental retardation protein (FMRP; the product of *Fmr1*) binds the *Itgb3* mRNA in the juvenile hippocampus and cerebellum,^82^ these data suggest therefore a potential convergence of the integrin and FMRP signaling pathways in ASD.

Finding new genetic approaches to correct metabotropic signaling in ASD is important because current pharmacological approaches based on mGluR antagonists, such as MPEP, albeit effective in preclinical studies, have failed to show significant benefits in humans.^34^ These failures may be due to the intrinsic inability of pharmacology to discriminate between synaptic and extra-synaptic mGluR5 and could be overcome by genetic approaches, such as ours, aimed at anchoring mGluR5 to the synapse. More in general, patients with ASD exhibit an increase in copy-number variations.^1^, ^2^, ^3^, ^4^ Further, many ASD mutations affect a single allele.^5^ These observations highlight the importance of gene dosage in ASD and the potential of CRISPRa-based strategies for rebalancing gene deficiency in ASD.

Here, we designed CRISPRa tools to activate *Itgb3*, which is deficient in some cases of ASD,^7^^,^^11^^,^^12^^,^^14^^,^^83^ and demonstrated that *Itgb3* haplo-insufficiency can be compensated for both *in vitro* and *in vivo* by activating the transcription of the remaining functional allele. By contrast, overexpression led to a ∼50-fold increase in β3 integrin protein levels, aberrant mGluR5 expression, and hyperactive cortical networks (Figures 5, 6, S4, and S10). Thus, whenever possible, CRISPRa should be preferred to overexpression of exogenous genes in rescue experiments and future therapeutic strategies. Although CRISPRa holds great promise for diseases caused by haplo-insufficiency, it is nevertheless not suitable for compensating dominant mutations as it would increase expression of both functional and aberrant alleles.

## Materials and methods

### Experimental model and subject details

All experiments were performed in accordance with EU and Italian legislation (authorization no. 1168/2020-PR). *Itgb3* KO and Het mice (B6;129S2-Itgb3tm1Hyn ⁄J, Jackson Laboratory) were described previously^30^^,^^31^ and were backcrossed to the C57BL⁄6j background >10 times at the time of experiments. For *in vivo* experiments, male CaMKIIa-CreTg/Tg mice^31^ were crossed with female *Itgb3*^+/−^ mice to obtain CaMKIIa-CreTg/+;Itgb3^+/+^ and CaMKIIa-CreTg/+;Itgb3^+/−^ littermates for rAAV injections.

### gRNA design and plasmid construction

We used the web tool http://crispr.mit.edu/, which maximizes the regions with low off-target probability, to design six gRNAs targeting the region from −100 to −200 bp relative to the TSS of the *Itgb3* gene. As negative Ctrl (gRNA Ctrl), we used a non-targeting gRNA sequence (Figure S3A). The pU6-(BbsI)-EF1a-dCas9-VP64-T2A-EGFP plasmid (Figure S3A), used to co-express a gRNA, the dCas9-VP64 fusion protein, and EGFP, was constructed by inserting the EF1a-dCas9-VP64-T2A-EGFP cassette from the dCAS9-VP64-GFP plasmid (gift from Feng Zhang; cat. no. 61422, Addgene)^58^ in place of the CBh-Cas9-T2A-mCherry cassette of the pU6-(BbsI)-CBh-Cas9-T2A-mCherry plasmid (gift from Ralf Kuehn; cat. no. 64324, Addgene). The gRNA sequences were inserted downstream of the U6 promoter using the BbsI cloning sites. The lentiviral vectors pLL-U6-(gRNA)-EF1a-dCas9-VP64-T2A-EGFP (Figure S4A) were constructed by inserting the cassette U6-gRNA Ctrl, U6-gRNA 2, or U6-gRNA 3 from the pU6-(gRNA)-EF1a-dCas9-VP64-T2A-EGFP plasmids described above in dCAS9-VP64-GFP (cat. no. 61422, Addgene) using the PacI and AgeI sites. Human β3 integrin was expressed under the control of the short human Synapsin promoter using the lentiviral vector pLL-Syn-EGFP-P2A-ITGB3.^31^ The rAAV vector pAAV-Syn-DIO-Sa-dCas9-VPR (Figure 8A) was constructed from pJEP313-pAAV-CMV-Sa-Cas9-DIO-pA (gift from Jonathan Ploski; cat. no. 113690, Addgene) by replacing the CMV promoter with the human Synapsin promoter from pAAV-hSyn-EGFP (gift from Bryan Roth; cat. no. 50465, Addgene) and Sa-Cas9 with Sa-dCas9-VPRmini from pAAV-CMV-dSa-VPRmini-syn-pA (gift from George Church; cat. no. 99689, Addgene).^84^ For the rAAV vectors pAAV-U6-gRNA-Syn-DIO-EGFP (Figure 8A), we first inserted the gRNA sequences downstream of the U6 promoter in BPK2660 (gift from Keith Joung; cat. no. 70709, Addgene)^85^ using the BsmbI cloning sites. We then inserted the cassette U6-gRNA Ctrl, U6-gRNA 4, or gRNA 5 from the BPK2660-gRNA plasmids into pAAV-hSyn-EGFP-DIO (gift from Bryan Roth; cat. no. 50457, Addgene) using the MluI cloning site. Constructs were generated by standard cloning strategies and verified by sequencing.

### N2a cell culture and transfection

N2a mouse neuroblastoma cells were cultured in Dulbecco’s modified Eagle medium (DMEM, Gibco) supplemented with 10% FBS, 2 mM glutamine, 100 U/mL penicillin, and 0.1 mg/mL streptomycin (complete culture medium) and were maintained in a 5% CO_2_ humidified incubator at 37°C.^86^ Transfection was performed in 60%–70% confluent cultures seeded in 6-well plates at 200,000 cells/well in complete culture medium the previous day. Cells were transfected with 3 μg DNA/well using the Ca^2+^ phosphate method^87^ and used 24–48 h post-transfection.

### Cell adhesion assay

N2a cells were trypsinized 2 days after transfection and seeded in complete culture medium on fibronectin-coated coverslips (5 μg/mL for 16 h; cat. no. F8141, Sigma) in 24-well plates at a density of 100,000 cells/well. After 1 h at 37°C, coverslips were washed 4 times with PBS to remove non-attached cells; remaining adherent cells were fixed in 4% PFA, stained with Hoechst, and mounted with ProLong Gold mounting medium (Thermo Fisher Scientific). To quantify the number of attached cells, three images per coverslip were taken using a Leica SP8 confocal microscope with a 40× oil immersion objective (numerical aperture [NA] 1.30); for each condition, six coverslips from three independent cultures were imaged in total.

### Primary cortical culture

Cortical neuronal cultures were prepared from P0 *Itgb3*^*+/+*^ (WT), *Itgb3*^*+/−*^ (Het), or *Itgb3*^*−/−*^ (KO) pups as previously described,^88^^,^^89^ with minor modifications. Briefly, cortices were dissected in ice-cold HBSS, digested with papain (30 U; cat. no. 3126, Worthington) for 40 min at 37°C, washed, and triturated in attachment medium (BME medium supplemented with 10% FBS, 3 mg/mL glucose, 1 mM sodium pyruvate, and 10 mM HEPES-NaOH [pH 7.40]) with a flame-polished glass Pasteur pipette. For qRT-PCR and western blot experiments, cells were seeded at a concentration of 750,000 cells/well onto 6-well plates coated with 2.5 μg/mL poly-D-lysine (PDL; P7405, Sigma) and 1 μg/mL laminin (L2020, Sigma); for confocal microscopy and Ca^2+^ imaging experiments, cells were seeded at 75,000/well onto 1.2 cm diameter glass coverslips coated with PDL/laminin as above. After 4 h, the attachment medium was replaced with maintenance medium (neurobasal medium supplemented with 2.6% B27, 6 mg/mL glucose, 2 mM GlutaMax, 90 U/mL penicillin, and 0.09 mg/mL streptomycin). To prevent glia overgrowth, 0.5 μM of cytosine β-D-arabinofuranoside (AraC) was added at 4–5 days *in vitro*.

### Lentivirus production and infection

HEK293T cells were maintained in Iscove’s modified Dulbecco’s medium supplemented with 10% FBS, 2 mM glutamine, 100 U/mL penicillin, and 0.1 mg/mL streptomycin in a 5% CO_2_ humidified incubator at 37°C. Cells were transfected with the Δ8.9 encapsidation plasmid, the VSVG envelope plasmid, and the pLL-U6-(gRNA)-EF1a-dCas9-VP64-T2A-EGFP or the pLL-Syn-EGFP-P2A-ITGB3 plasmid described above using the Ca^2+^ phosphate method. The transfection medium was replaced by fresh medium after 14 h. Supernatants were collected 36 to 48 h after transfection, centrifuged to remove cell debris, passed through a 0.45 μm filter, and ultra-centrifuged for 2 h at 20,000 g at 4°C. Viral pellets were re-suspended in PBS, aliquoted, and stored at −80°C until use.^87^ Neuronal cultures were infected at 6 DIV with the lowest infectious dose capable of transducing ≥95% of neurons (dilution range: 1:300 to 1:700) and used for experiments after ≥10 days (Figure S4A).

### Western blotting

Membrane protein-enriched fractions were prepared from cortical neurons at 16 DIV as previously described.^87^ Briefly, cells were washed once in ice-cold PBS and scraped in 100 μL buffer A (25 mM Tris-HCl [pH 7.4], 150 mM NaCl, 2 mM KCl, 2.5 mM EDTA) supplemented with protease and phosphatase inhibitors (complete EDTA-free protease inhibitors [cat. no. 1187358001, Roche]; serine/threonine and tyrosine phosphatase inhibitors [cat. nos. P0044 and P5726, Sigma]). After removal of the cell debris at 1,000 × *g*, 4°C, for 10 min, the supernatant was centrifuged at 15,000 × *g*, 4°C, for 15 min. The resulting pellet was dissolved in 100 μL RIPA buffer (50 mM Tris [pH 8.0], 150 mM NaCl, 1% NP-40, 0.5% sodium deoxycholate, 0.1% SDS) and centrifuged at 15,000 × *g*, 4°C, for 15 min. The resulting supernatant was used for western blot analysis. Protein concentration was quantified with the BCA Protein Assay kit (cat. no. 23227, Thermo Fisher Scientific). Proteins were separated by SDS-PAGE using 7.5% acrylamide gels and transferred on polyvinylidene fluoride (PVDF) membranes. After incubation with primary rabbit anti-β3 integrin (1:200; cat. no. 4702, Cell Signaling); rabbit anti-mGluR1 (1:1,000; cat. no. 191 002, Synaptic Systems); rabbit anti-mGluR5 (1:200; cat. no. AB5675, Millipore), mouse anti-GluA2 (1:500; cat. no. MAB397, Millipore); rabbit anti-GluA2/3 (1:200; cat. no. 07-598, Millipore); rabbit anti-GluA1 (1:500; cat. no. AB1504, Millipore); rabbit anti-GluA1 phosphoSer831 (1:500; cat. no. AB5847, Millipore); rabbit anti-GluA1 phosphoSer845 (1:500; cat. no. AB5849, Millipore); rabbit anti-Homer1a (1:1,000; cat. no. 160 013, Synaptic Systems); or rabbit anti-β-tubulin III (1:1,000; cat. no. T2200, Sigma) antibodies, membranes were incubated with secondary HRP-conjugated goat anti-rabbit antibody (1: 5,000; cat. no. 31460, Thermo Fisher Scientific) or goat anti-mouse antibody (1: 5,000; cat. no. 31430, Thermo Fisher Scientific), and immunocomplexes were detected with the chemiluminescent substrate (cat. no. RPN2106, ECL Prime Western Blotting System, GE Healthcare). We acquired chemiluminescent signals using a ChemiDoc imaging system (Biorad) and quantified immunoreactive bands using ImageJ (http://rsb.info.nih.gov/ij). Band intensity from different samples was normalized to that of WT Ctrl within the same membrane. Intensity of phosphorylated proteins was normalized to the total amount of the corresponding proteins.

### Immunoprecipitation

Adult male C57BL⁄6j mice (3–5 months) were deeply anesthetized and transcardially perfused with ice-cold phosphate buffer to remove β3-integrin-rich platelets from brain tissue. Cortices were immediately dissected on ice, flash frozen in liquid N_2_, and stored at −80°C until use. On the day of the experiment, cortices were defrosted on ice, homogenized with 15 strokes in buffer A (in mM: sucrose, 320; HEPES, 10; CaCl_2_, 0.2; MgCl_2_, 1; supplemented with protease inhibitors [complete EDTA-free protease inhibitors]) using a Douncer at 300 RPM. The homogenate was centrifuged at 1,000 × *g*, 4°C, for 10 min, and the supernatant collected and further centrifuged at 50,000 × *g*, 4°C, for 15 min. The pellet was then re-suspended in 0.5 mL buffer B (in mM: NaCl, 100; HEPES, 20; CaCl_2_, 0.2; MgCl_2_, 1; containing 1% Triton X-100, supplemented with complete EDTA-free protease inhibitors and serine/threonine and tyrosine phosphatase inhibitors) per mouse brain and incubated on ice for 15 min, before clearing the lysate from undissolved particles by centrifugation at 100,000 × *g*, 4°C, for 30 min. The so-obtained lysate was incubated overnight with Sepharose-ProteinG (Dynabeads, Thermo Fisher Scientific) coupled with hamster anti-CD61 (cat. no. 553343, BD Pharmingen) or rabbit anti-mGluR5 (cat. no. AB5675, Merck) in a total volume of 400 μL per sample. As negative controls served non-immune hamster IgG (cat. no. PA5-33219, Pierce) or rabbit anti-RFP (cat. no. 600-401-379, Rockland), respectively. The depleted lysate (flow trough) was removed, and the beads were washed 3 times in buffer B before elution of the immunoprecipitate with gel loading dye for 10 min at 70°C. Fractions were analyzed by western blot.

### RNA extraction and qRT-PCR

Total RNA was extracted with QIAzol lysis reagent (cat. no. 79306, Qiagen) from primary cortical cultures at 16 DIV or cortex of P60 mice, as previously described.^87^ We prepared cDNAs by reverse transcription of 1 μg of RNA using the QuantiTect Reverse Transcription Kit (cat. no. 205311, Qiagen). qRT-PCR was performed in triplicate with 10 ng of template cDNA using iQ SYBR Green Supermix (cat. no. 1708886, Biorad) on a CFX96 Real-Time PCR Detection System (Biorad) with the following universal conditions: 5 min at 95°C, 45 cycles of denaturation at 95°C for 15 s, and annealing/extension at 60°C for 45 s. Primers were designed with Primer-BLAST (www.ncbi.nlm.nih.gov/tools/primer-blast) to avoid significant crosshomology regions with other genes. Product specificity and absence of primer dimers was verified by melting curve analysis and agarose gel electrophoresis. qPCR reaction efficiency for each primer pair was calculated by the standard curve method with a five points serial dilution of cDNA. Calculated qPCR efficiency for each primer set was used for subsequent analysis. The relative quantification of gene expression was determined using the ΔΔCt method. Data were normalized to glyceraldehyde-3-phosphate dehydrogenase (GAPDH), β-actin (ACTB), and hypoxanthine phosphoribosyltransferase 1 (HPRT1) by the multiple internal Ctrl gene method with GeNorm algorithm. mRNA expression was normalized to WT Ctrl samples within the same qRT-PCR plate. Sequences of all the primers used are listed in Table S1.

### ChIP in primary neurons

After crosslinking with 1% formaldehyde for 10 min and quenching with 125 mM glycine for 5 min, we extracted the chromatin from transduced primary cortical neurons using the Chromatin Extraction kit (cat. no. ab117152, Abcam) according to the manufacturer’s instructions. After sonication, samples were further processed for ChIP using the ChIP Kit-One step (cat. no. ab117138, Abcam) and a rabbit polyclonal anti-Cas9 antibody (cat. no. C15310258, Diagenode) or a Ctrl non-immune immunoglobulin G (IgG) (cat. no. ab117138, Abcam; Figure S5B). Enrichment of target regions was assessed by qRT-PCR as detailed in previous section using the primers listed in Table S1.

### *In vivo* genome-wide ChIP-seq

Chromatin was extracted from cortices of P60 mice (two biological replicates per condition) injected with pAAV-Syn-DIO-Sa-dCas9-VPR and pAAV-U6-gRNA-Ctrl-Syn-DIO-EGFP (gRNA Ctrl condition; 1:2 rAAV ratio) or pAAV-Syn-DIO-Sa-dCas9-VPR, pAAV-U6-gRNA-4-Syn-DIO-EGFP, and pAAV-U6-gRNA-5-Syn-DIO-EGFP (gRNA 4+5 condition; 1:1:1 rAAV ratio) and prepared using the SimpleChIP Plus Enzymatic Chromatin IP Kit (cat. no. 9005, Cell Signaling) according to the manufacturer’s instructions. After chromatin shearing, immunoprecipitation was performed using an anti *S. aureus* Cas9 antibody (cat. no. C15200230, Diagenode). Library preparation and sequencing on the Illumina HiSeq/NovaSeq platform were performed by Novogene (Cambridge, UK). Sequencing reads were mapped to the genome using BWA.^90^ Mapping was restricted to reads that were uniquely assigned to the mouse genome (GRCm38.p6). Biological replicates were pooled to call peaks of gRNAs 4 + 5 versus gRNA Ctrl using MACS2.^91^

### Confocal microscopy and image analysis

Ten days post-infection, cultures were fixed for 8 min with 4% PFA/4% sucrose at room temperature (RT), treated for 10 min at 50°C with a sodium citrate solution (10 mM tri-sodium citrate dihydrate [pH 6.0], 0.05% Tween-20) to retrieve the β3 integrin antigen,^92^ and permeabilized for 10 min at RT with 0.1% TritonX-100. β3 integrin staining was revealed using a rabbit monoclonal anti-integrin β3 (1:200; cat. no. 13166, Cell Signaling) and the Tyramide SuperBoost kit (cat. no. B40922, Thermo Fisher Scientific) with the Alexa Fluor 568 Tyramide Reagent (10 min, 1:10 dilution; cat. no. B40956, Thermo Fisher Scientific) before counterstaining for GFP and vGlut1 using chicken anti-GFP (1:1,000; cat. no. AB13970, Abcam) and guinea pig anti-vGlut1 (1:500; cat. no. 135304, Synaptic System), respectively. For mGluR5, we used rabbit anti-mGluR5 (1:200; cat. no. AB5675, Millipore) at 16 DIV without antigen retrieval treatment. Secondary antibodies were Alexa Fluor 488-conjugated anti-chicken (1:1,000; cat. no. A11039, Thermo Fisher Scientific); Dylight405-conjugated donkey anti-guinea pig (1:150; cat. no. 706-475-148, Jackson ImmunoResearch); Alexa Fluor 488-conjugated anti-rabbit (1:1,000; cat. no. A11034, Thermo Fisher Scientific); and Alexa Fluor 647-conjugated anti-guinea pig (1:1,000; cat. no. A21450, Thermo Fisher Scientific). Confocal stacks were acquired at 200 Hz with a Leica SP8 using a 63× oil immersion objective (NA 1.40), 1.2× digital zoom, 0.15 μm pixel size, 1 AU pinhole, 0.3 μm between optical sections, with a sequential line-scan mode and 3× scan averaging. For all experimental conditions compared, we used the same settings for laser intensity, offset, and PMT gain. Confocal images were analyzed using ImageJ. Each stack was filtered using a Gaussian filter (radius: 0.5 pixels), and the maximal fluorescence intensities of in-focus stacks were Z-projected. For Figure S4, the images were automatically thresholded using the Robust Automatic Threshold Selection plugin followed by the watershed algorithm. Dendritic analysis was performed on dendritic regions of interest (ROIs) of 40–120 μm in length, manually selected in the GFP channel blind to the experimental condition. Co-localization was estimated for the thresholded ROIs with the Coloc2 plugin using the Manders’ coefficients (M_A_ = ∑_i_ A_i,coloc_/∑_i_ A_i_, where ∑_i_ A_i_ is the sum of intensities of all pixels above threshold for channel A and ∑_i_ A_i,coloc_ is calculated as ∑_i_ A_i_ but only for pixels where also the second channel B is above threshold). For Figure 4, the images were automatically thresholded using the Auto Local Threshold Otsu with a radius of 15 pixels. Blind to the genotype, we used the Time Series Analyzer v.3 plugin to position circular ROIs (Ø = 2.17 μm) on vGlut1-positive dendritic protrusions (operationally defined as dendritic spines). These were used to obtain the synaptic signal (S) in the mGluR5 channel. For the dendritic mGluR5 signal (D), we positioned a second ROI adjacent to the first on the parental dendritic shaft. The intensity of a third ROI positioned within 10 μm from the first two was used to subtract the local background noise. Background subtracted S and D signals were quantified as relative difference ((S−D)/(S+D)) for each spine/dendritic shaft pair. P60 mice were anesthetized and intracardially perfused with 4% PFA. The brain was postfixed, cryoprotected in 30% sucrose, and embedded in optical cutting temperature (OCT) compound; frozen sagittal sections (40 μm) were cut with a cryostat. Sections were permeabilized with 0.3% Triton X-100 for 10 min, blocked for 30 min with 5% normal goat serum (NGS), and then incubated with primary antibodies (chicken anti-GFP and mouse anti-NeuN; 1:500; cat. no. MAB377, Merck-Millipore) for 2 h and with secondary antibodies (Alexa Fluor 488-conjugated anti-chicken and Alexa Fluor 568-conjugated anti-mouse; 1:500; cat. no. A11004, Thermo Fisher Scientific) for 1 h. Sections were imaged using a Nikon Eclipse E800 epifluorescence microscope with a 20× objective.

### MEA recordings

Cortical neurons were seeded at 150,000 cells/well on 6-well MEAs (Multichannel Systems) coated with PDL/laminin. Each well contained nine electrodes (30 μm diameter; 200 μm center-to-center spacing). Neurons were transduced at 6 DIV, and network activity was recorded at 16–17 DIV using a MEA1060INV amplifier (Multichannel Systems). Neurons were kept in maintenance medium at 37°C throughout the recordings. To ensure stabilization of the electrical signal, experiments were initiated 10 min after transferring the MEAs from the incubator to the setup. Network activity was recorded for 5 min under basal conditions and for a further 5 min at the indicated time points upon drug application.

Spike detection and spike train analysis were performed with the MC-Rack software (Multichannel Systems). Spike threshold was set for each electrode at 5 times the standard deviation of the baseline noise level. Bursts were operationally defined as a collection of a minimum number of spikes (N_min_ = 5) separated by a maximum interspike interval (ISI_max_ = 100 ms). Following spike and burst detection, we computed mean firing rate, burst rate, mean burst duration, percentage of spike in burst, and intraburst spike frequency. To evaluate network synchrony, we computed the Pearson’s correlation coefficient (*r*) of burst activity for all electrode pairs from each MEA well. Electrodes were not included in the analysis if they recorded less than 5 bursts over 5 min recording period.

### Ca^2+^ imaging

Imaging was performed in primary cortical cultures at 30°C ± 2°C in aCSF containing (in mM): 140 NaCl, 3.5 KCl, 2.2 CaCl_2_, 1.5 MgCl_2_, 10 D-glucose, and 10 HEPES-NaOH (pH 7.38; osmolarity adjusted to 290 mOsm). In initial experiments, we tested the red-shifted genetically encoded Ca^2+^ indicators (GECIs) jRGECO1a, jRCaMP1a, and jRCaMP1b^38^ and chose jRCaMP1b for subsequent experiments as it provided overall the best signal-to-noise ratio, the largest dynamic range, and the best temporal resolution of the three GECIs in our experimental conditions. Cultures were infected with the appropriate lentivirus and pAAV.Syn.NES-jRCaMP1b.WPRE.SV40 (cat. no. 100851-AAV1, Addgene; titer: 1.7∗10^13^ GC/mL; dilution 1: 50,000) 9–12 and 4–6 days prior to experiments, respectively, and recorded at 15–18 DIV. Imaging was performed with a cooled charge-coupled device (CCD) camera (ORCA-R2, Hamamatsu) mounted on an inverted microscope (DMI6000B, Leica) with a 20×, 0.75 NA glycerol immersion objective. A 200 W metal halide lamp (Lumen200Pro, Prior Scientific) and a filter set comprising a BP 515–560 nm excitation filter, a 580 nm dichroic mirror, and an LP 590 emission filter (filter set N2.1, Leica) were used for illumination. Images were captured at 15.3 Hz with 50 ms integration times at a depth of 8 bits. Network activity was recorded for 5 min. Images were analyzed in ImageJ with the plugin Time Series Analyzer v.3.0. ROIs were manually drawn on the soma (excluding the nuclear region; Figure 6A) of each neuron exhibiting at least one spontaneous fluorescence transient above two SD of the background noise during 5 min recording period. The intensity of twin ROIs positioned within 50 μm were used to subtract the background noise. Signals were quantified as ΔF/F_0_, where ΔF = F − F_0_, with F_0_ measured over 1 s period preceding the fluorescence transient. The Pearson’s correlation coefficient for fluorescence transient amplitude was computed for all neuron pairs in each field of view.

### rAAV production and injection

rAAVs (AAV-PHP.eB serotype) were prepared by the AAV Vector Unit at the International Centre for Genetic Engineering and Biotechnology Trieste, as previously described.^93^ Injections were performed into the lateral ventricles (two-fifths of the distance from the lambda suture to each eye) at P0 (1.5 μL per ventricle), as previously described.^94^

### Statistical analysis

Statistical differences were assessed using unpaired two-tailed Student’s t test and one-way analysis of variance (ANOVA) test followed by Tukey-Kramer post-test and two-way ANOVA, as required. The chi-square test was used for Figures 3I, 5I, 6E, 7I, S6F, and S8C (Prism 7, GraphPad Software). Average data are expressed as mean ± SEM.

### Data availability statement

All data generated or analyzed during this study are included in this published article and its supplemental information files.

## Supplemental information

Document S1. Figures S1–S10 and Table S1

Document S2. Article plus supplemental information
