# Supplementary material for: CRISPR-mediated activation of autism gene Itgb3 restores cortical network excitability via mGluR5 signaling
Source: Mol Ther Nucleic Acids. 2022 Jul 20;29:462–80. doi: 10.1016/j.omtn.2022.07.013 (PMC9382421; doi:10.1016/j.omtn.2022.07.013)
Supplement: Document S1. Figures S1–S10 and Table S1 [file mmc1.pdf]

OMTN, Volume *29*

Supplemental information

CRISPR-mediated activation of autism gene *Itgb3* restores cortical network excitability via mGluR5 signaling

Fanny Jaudon, Agnes Thalhammer, Lorena Zentilin, and Lorenzo A. Cingolani

SUPPLEMENTAL MATERIAL

SUPPLEMENTAL FIGURES AND TABLES

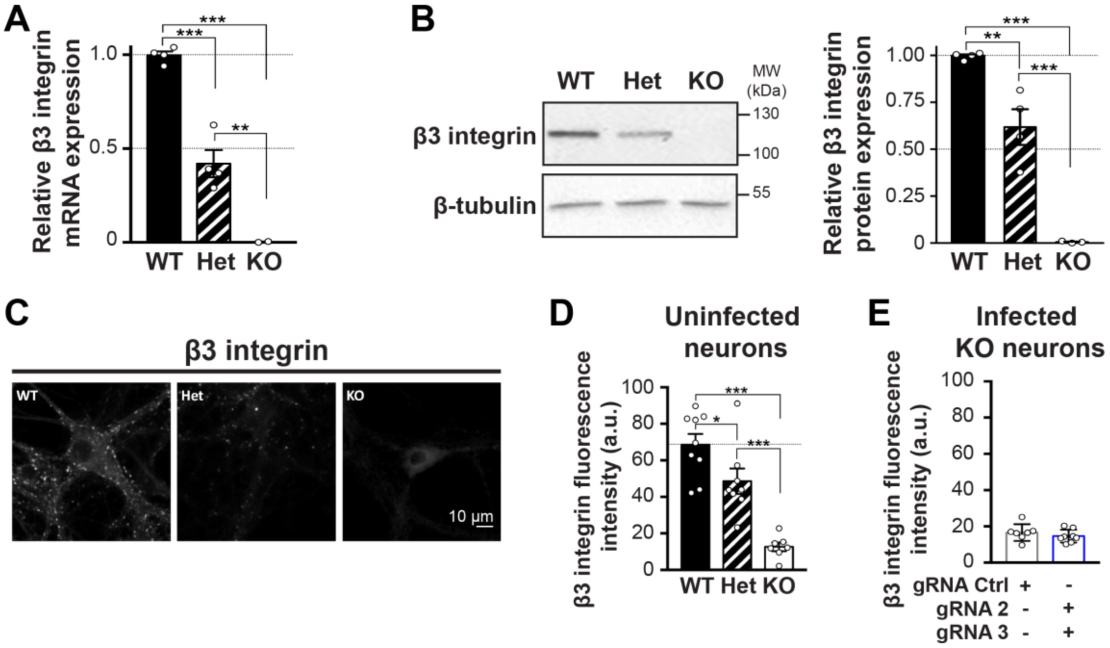

Figure S1. β3 integrin expression in primary cortical neurons. (A) Expression of β3 integrin mRNA in mouse primary cortical neurons at 16 DIV. Values are normalized to WT samples within the same RT-qPCR plate (n=4, 4 and 2 independent cultures for WT, *Itgb3* Het and KO, respectively). **(B)** Membrane protein fractions from mouse primary cortical neurons were analysed by Western blotting at 16 DIV. Left, representative immunoblots for β3 integrin; β-tubulin was used as a loading control. Right, quantification of immuno-reactive bands; band intensities were normalized to WT within the same membrane (n=4, 4 and 3 independent cultures for WT, Het and KO, respectively). **(C)** Representative confocal images of WT, *Itgb3* Het and KO primary cortical neurons stained for β3 integrin at 16 DIV. **(D)** Quantification of experiments as in (C; n=9, 8 and 7 images for WT, Het and KO, respectively). **(E)** Infection with CRISPRa and gRNAs 2+3 does not increase β3 integrin expression in *Itgb3* KO cultures (n=7 and 8 images for gRNA Control and gRNAs 2+3, respectively). Data are presented as mean±SEM; dots represent individual values (*p<0.05, **p<0.01, ***p<0.001, one-way ANOVA followed by Tukey post-test for panels A, B and D; p=0.37, unpaired Student's t-test for panel E).

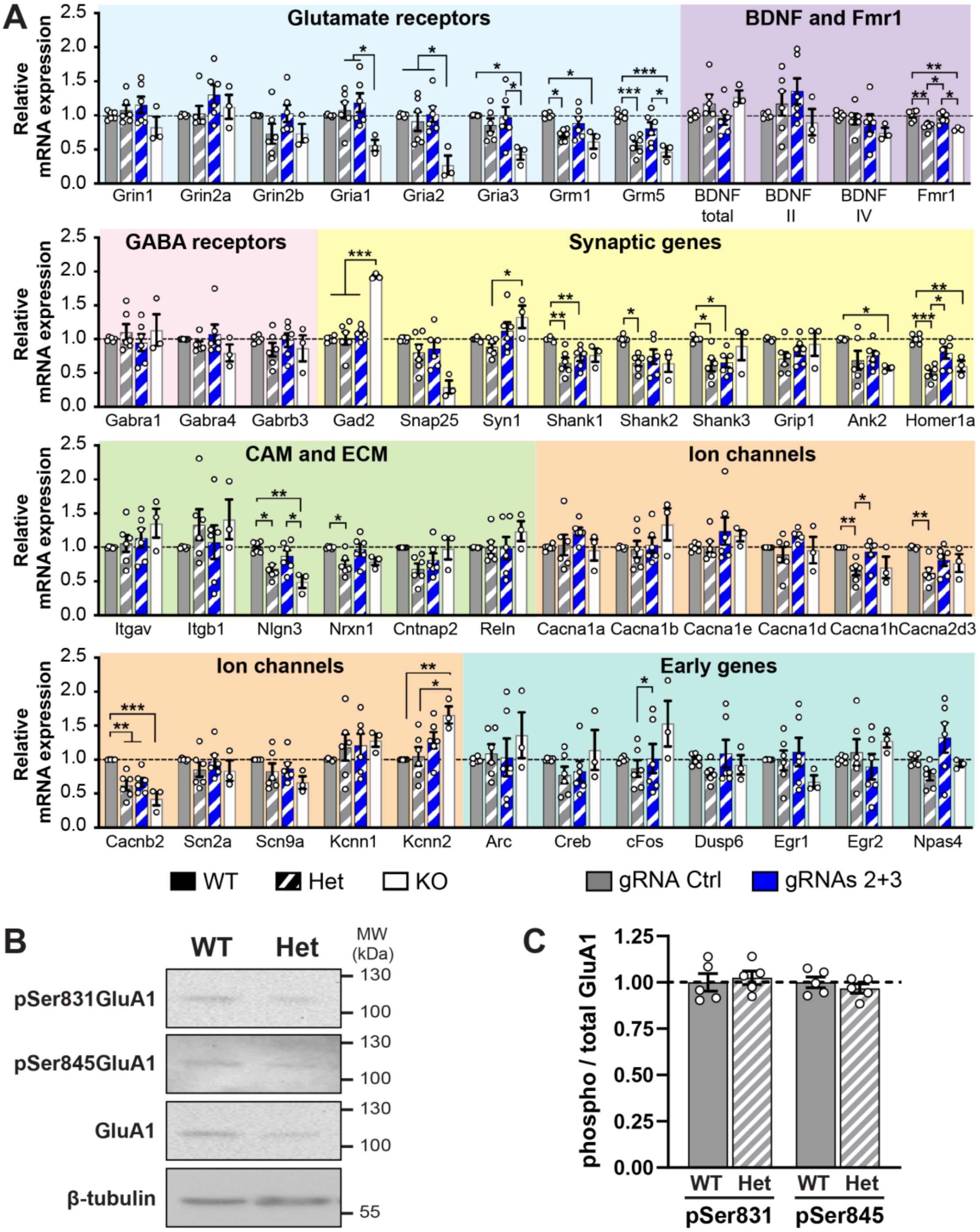

Figure S2. Regulation of neuronal gene expression by β3 integrin. (A) RT-qPCR quantification of mRNA expression for 48 neuronal genes (grouped into functional categories as labelled) in WT, *Itgb3* Het and KO cortical neurons expressing the indicated constructs (*p<0.05, **p<0.01, ***p<0.001, one-way ANOVA followed by Tukey post-test; n=6, 6 and 3 independent cultures for WT, Het and KO, respectively; 2 technical replicates each). **(B)** Representative Western blots of membrane-enriched fractions from WT and *Itgb3* Het cortical neurons. **(C)** Quantification of experiments as in (B) showing that phosphorylation levels of GluA1 are not altered in Het neurons (unpaired Student's t-test; n=5 independent cultures). Data are shown as mean±SEM; dots represent individual values.

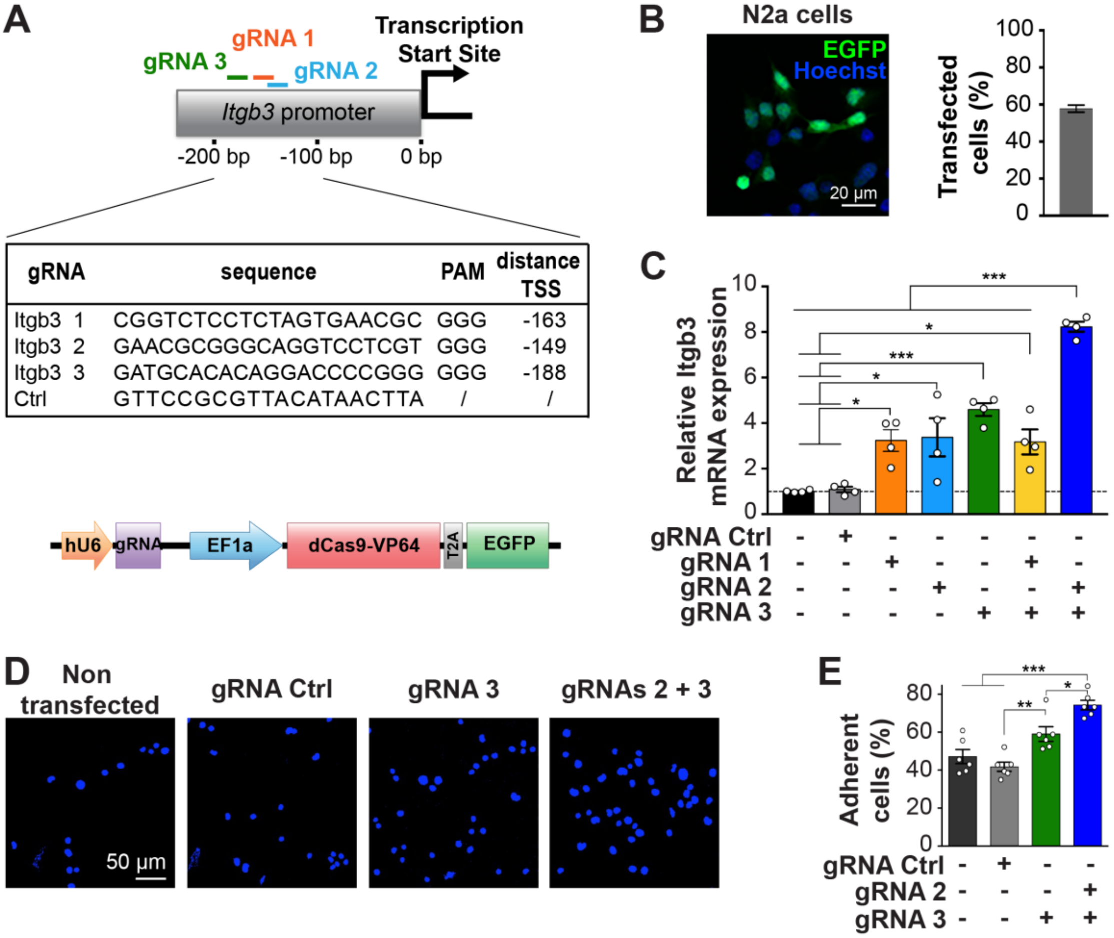

Figure S3. CRISPR/dCas9-mediated enhancement of β3 integrin expression in N2a cells. (A) Top, gRNA sequences and position of their targets on the *Itgb3* promoter. Bottom, construct used for transfecting murine N2a cells, containing a cassette for expressing gRNA and one for expressing dCas9-VP64 and EGFP. **(B)** Representative image of transfected N2a cells (left) and quantification of transfection efficiency (right). **(C)** Quantification of β3 integrin mRNA levels in N2a cells 24 hours after transfection with the indicated constructs. mRNA expression values were normalized to those of non-transfected samples within the same RT-qPCR plate (n=4 independent cultures each; 2 technical replicates per culture). **(D-E)** Cell adhesion assay for N2a cells transfected with the indicated constructs and plated onto fibronectin-coated coverslips. Representative images of adherent cells stained with Hoechst (D) and quantification of the percentage of adherent cells (E; n=6 each from 3 independent cultures). Data are presented as mean±SEM; dots represent individual values (*p<0.05, **p<0.01, ***p<0.001, one-way ANOVA followed by Tukey post-test).

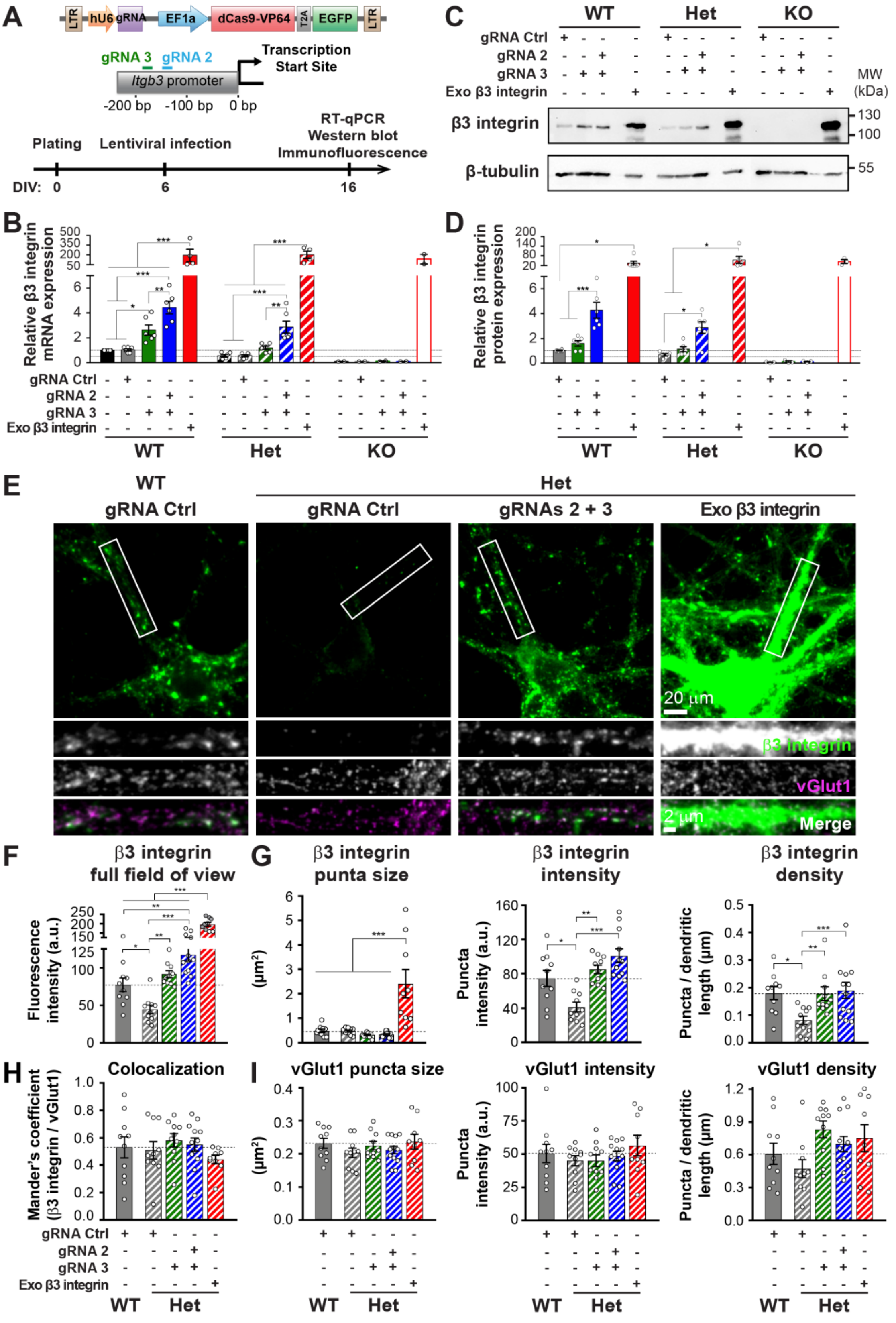

Figure S4. Rescue of β3 integrin expression by CRISPRa. (A) Scheme of lentiviral construct, gRNA targets on the *Itgb3* promoter and experimental timeline. **(B)** RT-qPCR quantification of β3 integrin mRNA expression in WT, *Itgb3* Het and KO cortical neurons transduced with the indicated constructs. (n=6, 6 and

2 independent cultures for WT, Het and KO, respectively; 2 technical replicates each). **(C)** Representative Western blots of membrane fractions. **(D)** Quantification of experiments as in (C; n=6, 6 and 3 independent cultures for WT, Het and KO, respectively; 2 technical replicates each). CRISPRa, but not over-expression of exogenous β3 integrin, rescues *Itgb3* gene dosage in *Itgb3* het neurons at both the mRNA and protein level. **(E)** Representative confocal images of primary cortical neurons from WT and Het cultures expressing the indicated constructs. β3 integrin and the presynaptic marker vGlut1 are shown in false colors; infection was confirmed by EGFP. **(F)** Quantification of β3 integrin fluorescence intensity for the full field of view for experiments as in (E), indicating that CRISPRa elevates β3 integrin expression in Het to WT values while exogenous expression of β3 integrin increases several-fold the signal for this protein (the fluorescence intensity for Het+exogenous β3 integrin is a lower estimate because of pixel saturation; gray filled circles indicate pixel saturation >30%). **(G)** Left panel, dendritic puncta size for β3 integrin; expression of exogenous β3 integrin resulted in large dendritic areas of saturated signal, which were no further analyzed. Middle and right panels, quantification of the effects of the indicated gRNAs on dendritic puncta intensity and number for β3 integrin. **(H)** Mander's co-localization coefficient of β3 integrin with vGlut1. **(I)** Quantification of dendritic puncta size, intensity and number for vGlut1 (n=10, 10, 10, 12 and 9 from 3 independent cultures each for WT+gRNA Ctrl, Het+gRNA Ctrl, Het+gRNA 3, Het+gRNAs 2+3 and Het+exogenous β3 integrin, respectively). Data are presented as mean±SEM; dots represent individual values (*p<0.05, **p<0.01, ***p<0.001, one-way ANOVA followed by Tukey post-test).

A

	Off-target sequences	Number of mismatches	Genomic coordinates (GRCm38.p4 C57BL/6J)	Position	Gene
**gRNA 2**	**GAACGCGGGCAGGTCCTCGT**				
#1	GAGCGCTGGCAGGTCCTCGG	3	Chr5: 128607080 - 128607099	Intergenic	
#2	GGATGGGGGAAGGTCCTCGT	4	Chr14: 65726265 - 65726284	Intronic	Scara5
#3	GACCGCAGAGAGGTCCTCGT	4	Chr7: 135803303 - 135803322	Intergenic	
#4	GATGGCAGGCAGTTCCTCGT	4	Chr6: 86380681 - 86380700	Intergenic	
#5	GGAGGGGGGCAGTTCCTCGT	4	Chr4: 44576073 - 44576092	Intronic	Pax5
#6	GAACTCCGACCGGTCCTCGT	4	Chr13: 34002574 - 34002593	Exonic	Serpinb6a
#7	AAACCCGGGAAGGTCCTAGT	4	Chr16: 89832908 - 89832927	Intronic	Tiam1
#8	GAAAGAGTGCAGGTCCTAGT	4	Chr11: 60178341 - 60178360	Intergenic	
#9	GAACACAGGCAGTTCCTCGG	4	Chr14: 64286180 - 64286199	Intronic	Msra
#10	GACCGAGGGCTGGTCCTCAT	4	Chr2: 58176765 - 58176784	Exonic	Gm13546
**gRNA 3**	**GATGCACACAGGACCCCGGG**				
#1	GATGCCCACAGGGCCCCGGG	2	Chr4: 108941267 - 108941286	Exonic	Rab3B
#2	CCTGAACGCAGGACCCCGGG	4	Chr12: 105964060 - 105964079	Intergenic	
#3	CTTGCACACAGGACCCTGGG	3	Chr6: 146208088 - 146208107	Intronic	Itpr2
#4	GGAGAAGACAGGACCCCGGG	4	Chr17: 68274174 - 68274193	Intergenic	
#5	CCTGTACACAGCACCCCGGG	4	Chr8: 122480377 - 122480396	Exonic	Ctu2
#6	GCTGAACTCAGCACCCCGGG	4	Chr6: 39621848 - 39621867	Intronic	Braf
#7	GATGAACAGAGGACCCGGGG	3	Chr17: 45669894 - 45669913	Exonic	Tmem63B
#8	GACACACCCAGGACCCCGGA	4	Chr1: 91602261 - 91602280	Intergenic	
#9	GAGGTACTCAGGACCCTGGG	4	Chr3: 134181351 - 134181370	Intergenic	
#10	CCTACACACAGGCCCCCGGG	4	Chr12: 117153148 - 117153167	Intronic	Ptprn2

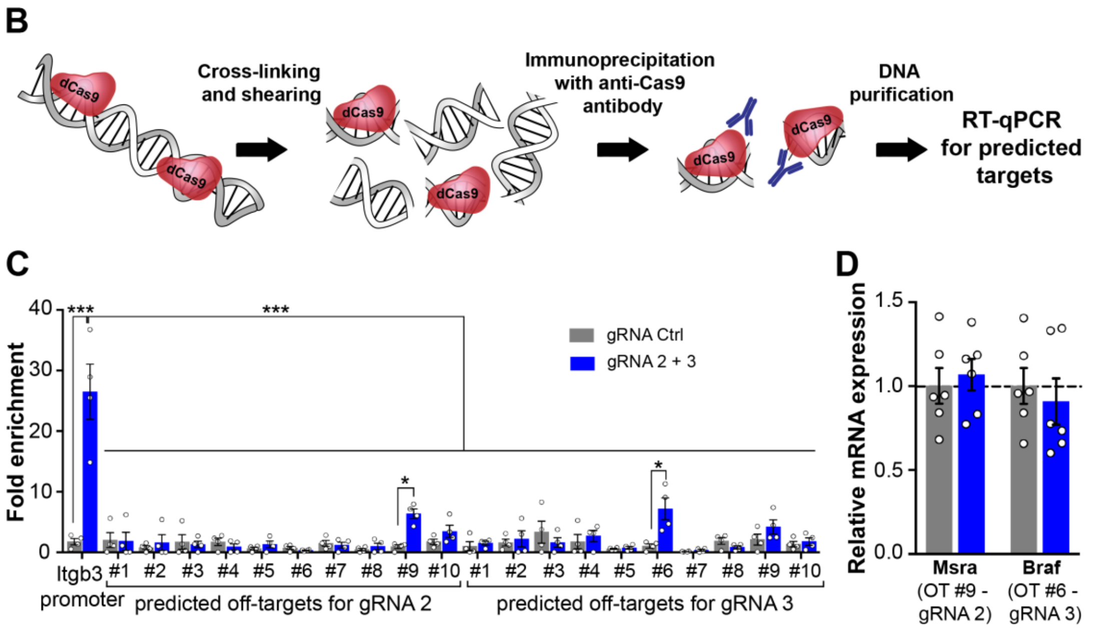

Figure S5. Target specificity of CRISPRa for β3 integrin in primary cortical neurons. (A) List of top-ten predicted off-targets for gRNAs 2 and 3 (http://crispr.mit.edu and https://crispr.cos.uni-heidelberg.de). The mismatches between the predicted off-targets and the on-target sequence are highlighted in red. Number of mismatches, genomic coordinates, position and name of potentially targeted genes are indicated. Scara5, scavenger receptor class A, member 5; Pax5, paired box protein 5; Serpinb6a, serine/cysteine peptidase inhibitor, clade B, member 6a; Tiam1, T cell lymphoma invasion and metastasis 1; Msra, mitochondrial peptide methionine sulfoxide reductase; Gm13546, predicted gene 13546, long non-coding RNA; Rab3B, member RAS oncogene family Rab3b; Itpr2, inositol 1,4,5-triphosphate receptor 2, transcript variant 2; Ctu2, cytosolic thiouridylase subunit 2; Braf, Braf transforming gene; Tmem63B,

transmembrane protein 63b; Ptprn2, protein tyrosine phosphatase, receptor type, N polypeptide 2. **(B)** ChIP-qPCR workflow. dCas9 co-expressed with gRNA Ctrl or gRNAs 2+3 is allowed to bind to chromatin. After cross-linking, the chromatin is sheared, immune-precipitated with an anti-Cas9 antibody and subjected to RT-qPCR. **(C)** Fold enrichment of dCas9 at on- and predicted off-target sites was calculated over an IgG control IP (*$p<0.05$, ***$p<0.001$, two-way ANOVA followed by Tukey post-test, n=4 independent cultures, 2 technical replicates each). **(D)** RT-qPCR quantification of mRNA expression for the two predicted off-target genes displaying significant dCas9 binding. Expression of both genes is not altered by dCas9-VP64 binding ($p\geq0.60$, unpaired Student's t-test, n=6 independent cultures, 2 technical replicates each). Data are presented as mean±SEM; dots represent individual values.

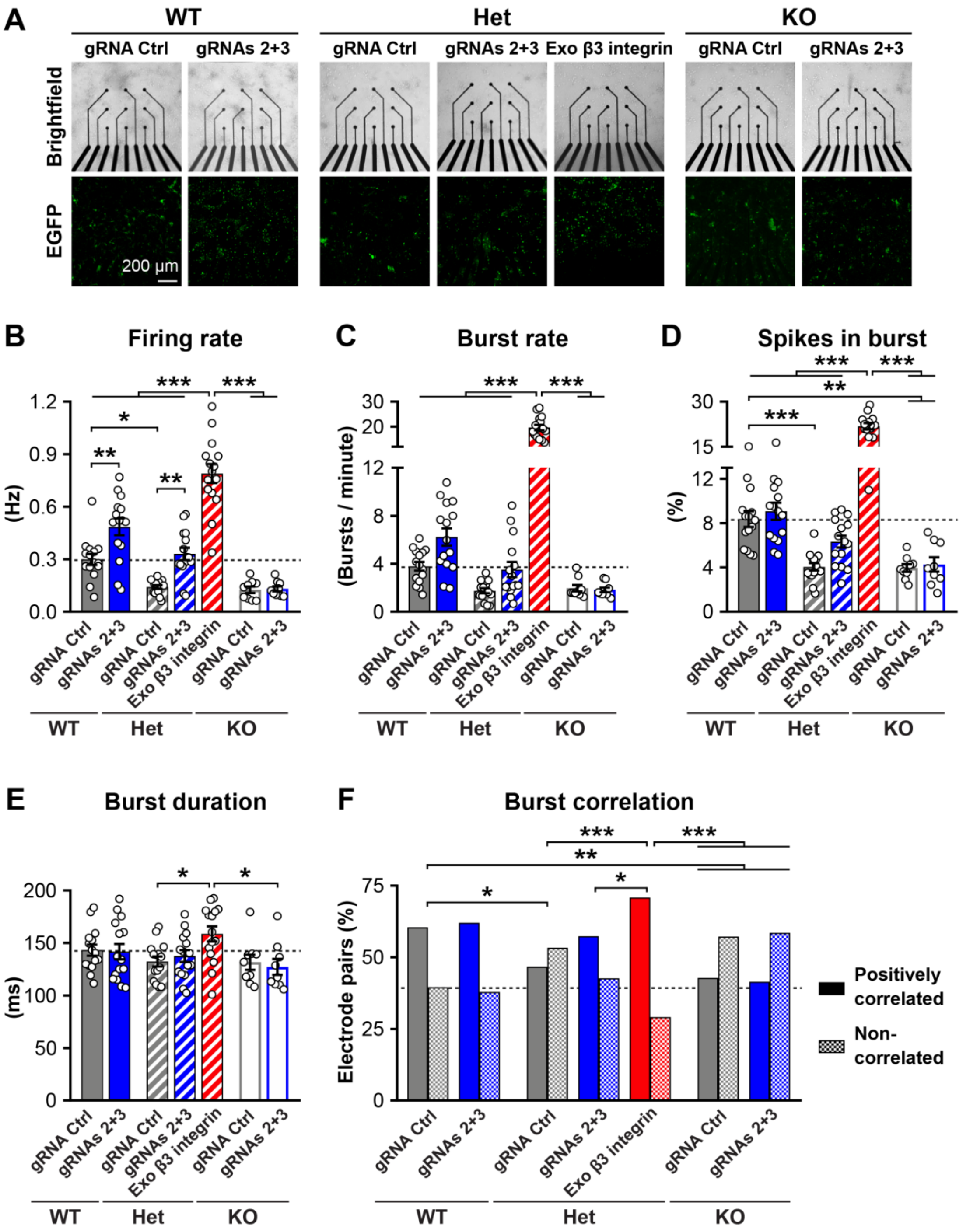

Figure S6. Further characterization of the effects of β3 integrin on network excitability. (A) Top, WT, *Itgb3* Het and KO cortical neurons expressing the indicated constructs plated on MEAs. Bottom, transduction efficiency was confirmed by EGFP expression. **(B-E)** Quantification of experiments as in Fig 5C-G. CRISPRa is effective in WT and *Itgb3* Het cultures but not *Itgb3* KO cultures (*p<0.05, **p<0.01, ***p<0.001, one-way ANOVA followed by Tukey post-test, n=15 each from 5 independent cultures). Data are presented as mean±SEM; dots represent individual values. **(F)** Quantification of Pearson's correlation coefficients (r) for burst activity as in Fig 5H, I. All electrode pairs exhibited a positive r. The graph shows the percentage of r with a p-value <0.05 (Positively

correlated) and a p-value >0.05 (Non-correlated; *p<0.05, **p<0.01, ***p<0.001, Chi-square test; n=473, 493, 447, 455, 532, 257 and 279 pairs for WT+gRNA Ctrl, WT+gRNAs 2+3, Het+gRNA Ctrl, Het+gRNAs 2+3, Het+exogenous β3 integrin, KO+gRNA Ctrl and KO+gRNAs 2+3, respectively).

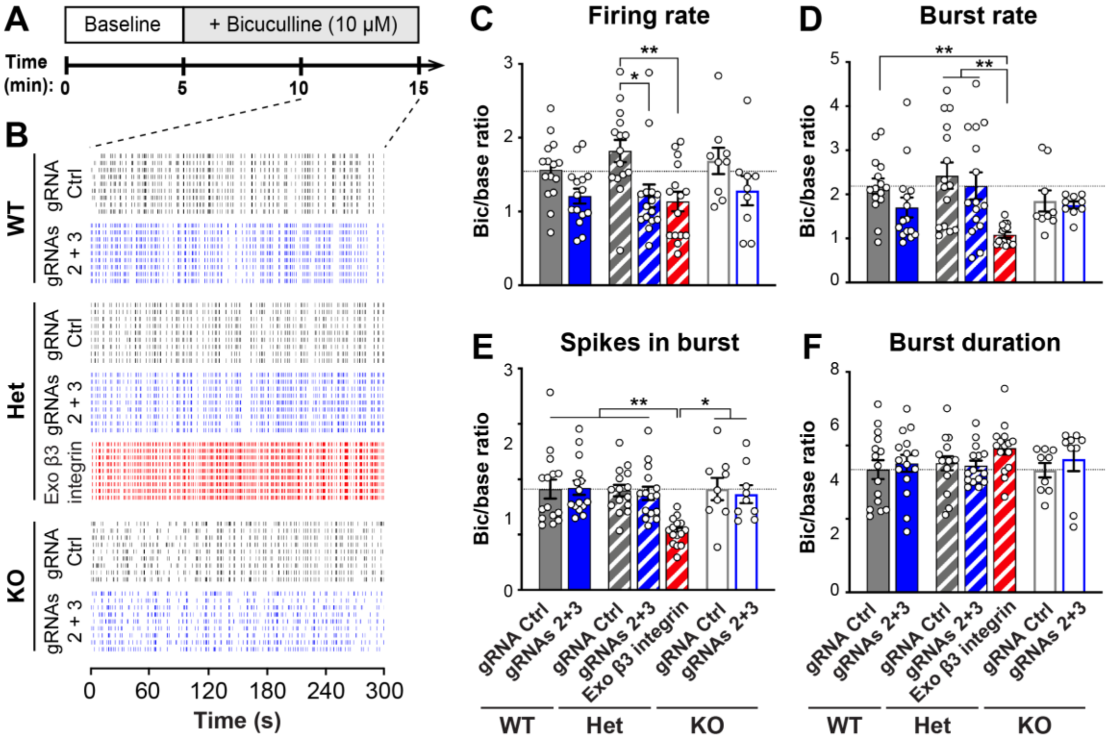

Figure S7. Effects of *Itgb3* expression levels on the bicuculline-dependent increase in network excitability. (A) Experimental timeline for bicuculline application (10 µM) in MEA experiments. **(B)** Representative raster plots of network activity after bicuculline application in WT, *Itgb3* Het and KO cultures expressing the indicated constructs. **(C-F)** Quantification of the bicuculline effects in experiments as in (A-B). Values are normalized to baseline for each recording (*p<0.05, **p<0.01, one-way ANOVA followed by Tukey post-test; n=15, 15 and 9 for WT, Het and KO, respectively; 5 independent cultures). Data are presented as mean±SEM; dots represent individual values.

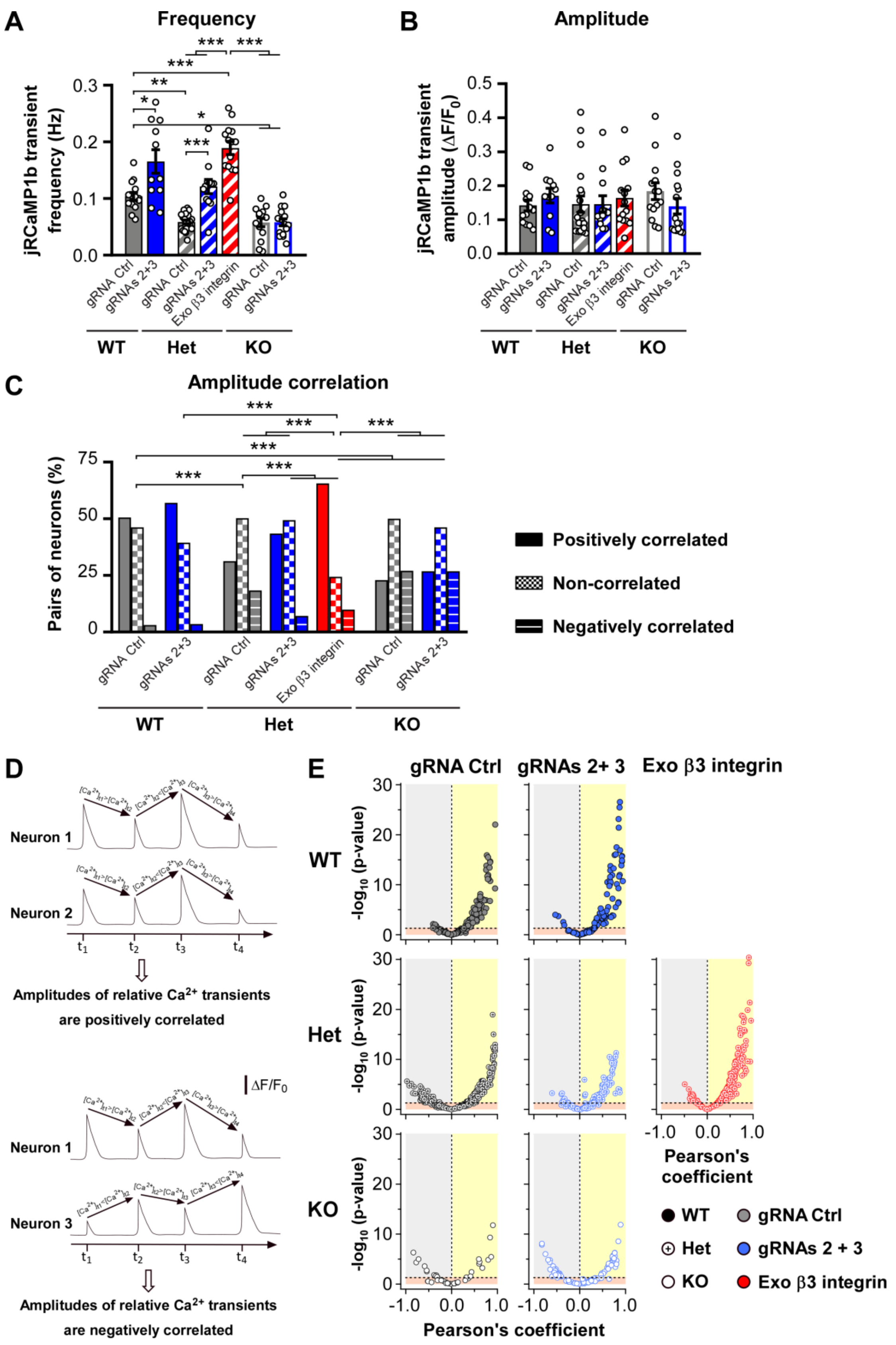

Figure S8. Further characterization of the effects of β3 integrin on jRCaMP1b fluorescence transients. (A-B) Quantification of experiments as in Fig 6A-C. CRISPRa is effective in WT and *Itgb3* Het cultures but not *Itgb3* KO cultures

(*p<0.05, **p<0.01, ***p<0.001, one-way ANOVA followed by Tukey post-test, n=14, 11, 20, 12, 14, 14 and 15 fields of view for WT+gRNA Ctrl, WT+gRNAs 2+3, Het+gRNA Ctrl, Het+gRNAs 2+3, Het+exogenous β3 integrin, KO+gRNA Ctrl and KO+gRNAs 2+3, respectively; 4-5 independent cultures). Data are shown as mean±SEM; dots represent individual values. **(C)** Quantification of Pearson's correlation coefficients (*r*) for fluorescence transient amplitudes as in Fig 6D, E. The graph shows the percentage of positive *r* with a p-value <0.05 (Positively correlated), negative *r* with a p-value <0.05 (Negatively correlated) and *r* with a p-value >0.05 (Non-correlated;, ***p<0.001, Chi-square test; n=158, 114, 396, 168, 233, 48 and 119 pairs for WT+gRNA Ctrl, WT+gRNAs 2+3, Het+gRNA Ctrl, Het+gRNAs 2+3, Het+exogenous β3 integrin, KO+gRNA Ctrl and KO+gRNAs 2+3, respectively). **(D)** Graphical illustration of the correlation analysis for fluorescence transient amplitudes. Assuming steady state conditions in the jRCaMP1b experiments, differences in amplitude of fluorescence signals are indicative of relative differences in Ca^{2+} transients at different time points within one neuron. Although it is not possible to compare directly differences in amplitude of fluorescence transients across neurons (e.g. because of differences in jRCaMP1b expression), fluorescence amplitude profiles between pairs of neurons can be compared to reveal positive (top) or negative (bottom) correlation in Ca^{2+} transients. **(E)** Volcano plots of all data points used for panel (C) and Fig 6E. The Pearson's correlation coefficient of fluorescence transient amplitudes for pairs of neurons is plotted against the $-\log_{10}$ of its p-value. Grey, pink and yellow backgrounds indicate negative, non-significant and positive correlation, respectively.

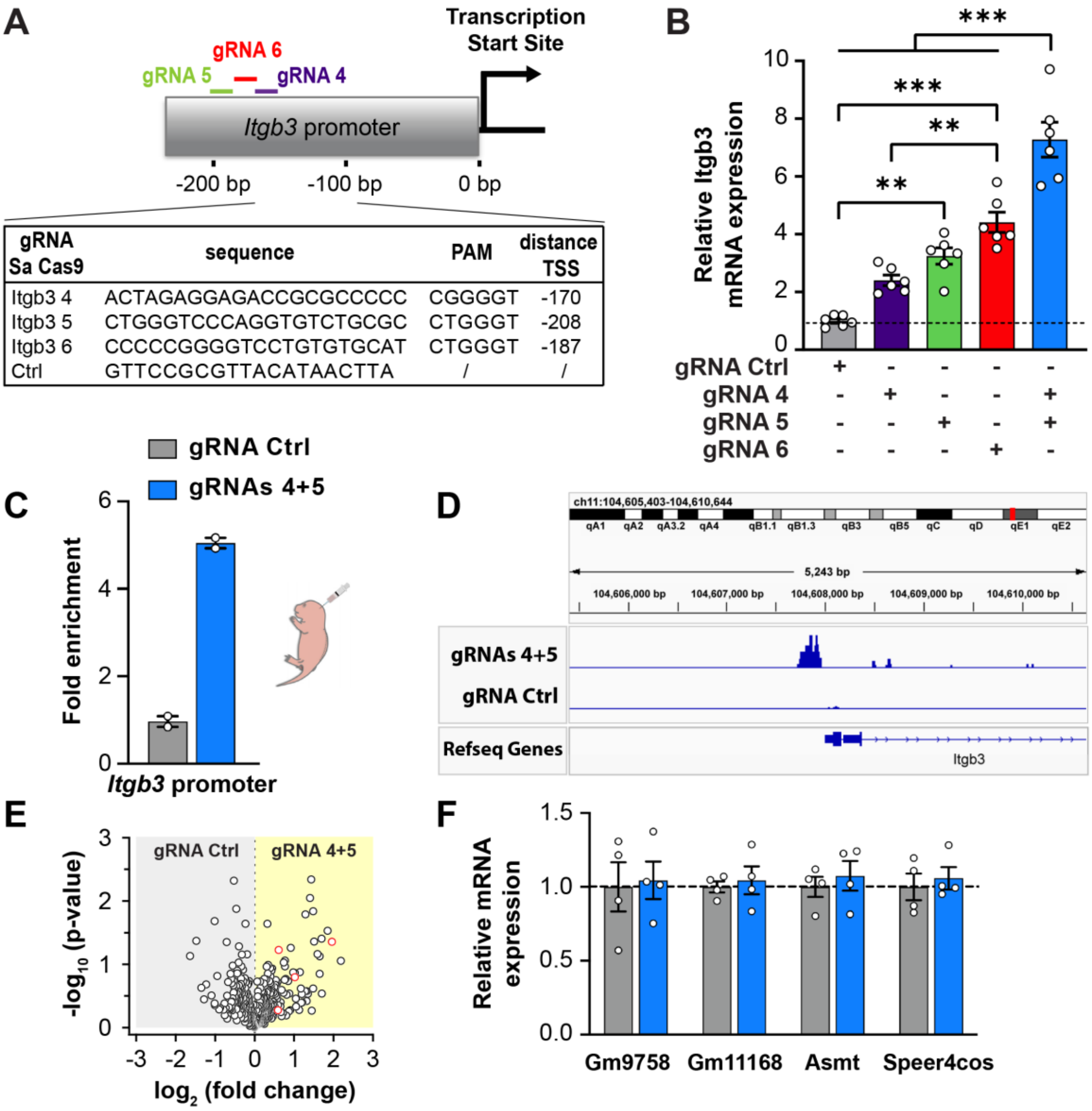

Figure S9. Efficacy and specificity of *S. aureus* Cas9-mediated CRISPRa for β3 integrin. (A) gRNA sequences for Sa-Cas9 and position of their targets on the *Itgb3* promoter. **(B)** Quantification of *Itgb3* mRNA levels in N2a cells 72 hours after transfection with Sa-dCas9-VPR and the indicated constructs. mRNA expression values were normalized to those of gRNA Ctrl-expressing samples within the same RT-qPCR plate (**p<0.01, ***p<0.001, one-way ANOVA followed by Tukey post-test; n=3 independent cultures each; 2 technical replicates per culture). **(C)** *In vivo* fold enrichment of Sa-dCas9-VPR at the *Itgb3* promoter calculated over an IgG control IP (n=2 cortices per condition). **(D)** Integrative genomics viewer snapshot of the peak at the *Itgb3* promoter in *in vivo* ChIP-seq experiments. **(E)** Volcano plot of the genome-wide off-target peaks for *in vivo* ChIP-seq experiments. The $\log_2$ fold change of gRNAs 4 + 5 over gRNA Ctrl (n = 2 cortices per condition) is plotted against the $-\log_{10}$ of the p-value for each peak (n = 494). None of the peaks is significantly enriched in either gRNA Ctrl (grey background) or gRNAs 4 + 5 condition (yellow background; no p-value was recognized as 'discovery' using the two-stage linear step-up procedure of

Benjamini, Krieger and Yekutieli for the false discovery rate with Q = 1 or 5%). Red circles indicate genes with a peak within 1 kb of their TSS. **(F)** RT-qPCR quantification of mRNA expression for the four genes marked in red in (E) indicates no change in their expression ($p \geq 0.56$, unpaired Student's t-test, n=4 cortices per group). *Gm9758*, predicted gene 9758; *Gm11168*, predicted gene 11168; *Asmt*, acetylserotonin O-methyltransferase; *Speer4cos*, spermatogenesis associated glutamate (E)-rich protein 4C. Data are presented as mean±SEM; dots represent individual values.

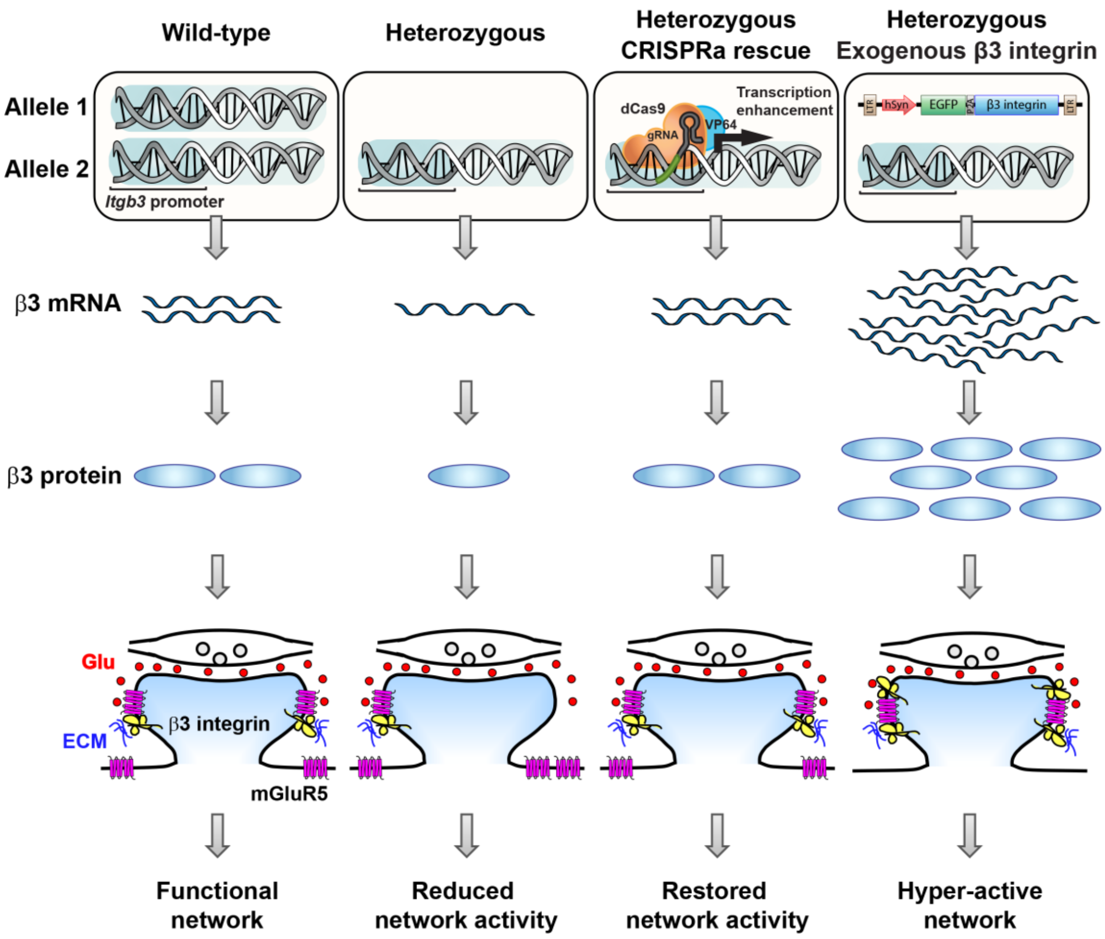

Figure S10. Working model comparing the effects of CRISPRa and over-expression on β3 integrin signalling. In Het neurons, the remaining allele of *Itgb3* produces an amount of mRNA and protein for β3 integrin that is 50% of that in WT neurons, with a consequent reduction in synaptic mGluR5 signalling and network excitability. By enhancing expression of the remaining allele, CRISPRa restores β3 integrin levels back to WT values, thus precisely rebalancing mGluR5 expression and network activity. Exogenous β3 integrin fails to mimic WT conditions, leading to un-physiologically high mRNA and protein levels for β3 integrin that result in hyperactive networks.

Table S1. List of primers used

RT-qPCR primers Gene	GenBank Accession	Forward sequence (5' → 3')	Reverse sequence (5' → 3')
m.Itgb3	NM_016780.2	GGGCGTTGTTGTTGGAGAG	ACAAAGTCTCATCTGAGCACCAG
h.ITGB3	NM_000212.2	CATCTCTGGGGCTGATGACT	GAGCGGATTTTCCCGTAAGC
Grin1	NM_008169.3	AAACCAGGCCAATAAGCGAC	GCGTAGACCTGGCTAGAGAT
Grin2a	NM_008170.2	GGTCAGCTTGAAAACTGGGAAG	AGATGTACCCGCTCCCAATG
Grin2b	NM_008171.3	CCTCCTGTGTGAGAGGAAATCT	CTCCTGGGTTGGGAAGTTCA
Gria1	NM_001113325.2	TGTGTTTGTTCGGACCACAG	GAGCACTGGTCTTGTCCTTAC
Gria2	NM_001083806.2	ATGGTTGTCACCCTAACCGA	AACGCTCATTCCCTTCAAGC
Gria3	NM_016886.4	CTCAGCATTAGGAACGCCTG	TTCCCCCTTATCGTACCACC
Grm1	NM_016976.3	CTGATTCACACACCTTCGGG	CCAAACCCTAGGGGTGTTCT
Grm5	NM_001081414.2	AGCGCACCTGGTGATTTTAC	ATGGGAGGCTTCAGCATACA
Gabra1	NM_010250.5	AAAAGCGTGGTTCCAGAAAA	GCTGGTTGCTGTAGGAGCAT
Gabra4	NM_010251.2	AGAACTCAAAGGACGAGAAATTGT	TTCACTTCTGTAACAGGACCCC
Gabrb3	NM_008071.3	TGCATTGAAAGGTGCCATGT	TATGGTGCATGAGCCACTCT
Gad2	NM_008078.2	GGAATCTTTTCTCCTGGTGGC	ATCAAAAGCCCCATACACGG
Snap25	NM_011428.3	CCTAGTAGGTCTTGCACATACAC	GACAGAGCACACAGGACATTT
Syn1	NM_013680.4	AGCTCAACAAATCCCAGTCTCT	CGGATGGTCTCAGCTTTCAC
Shank1	NM_001034115.1	GCACCCTTTCTTTCTCTAGCC	TATGGGAGTATGCCTGGGTC
Shank2	NM_001081370.3	GAGGAACTCGTGGACAAAGC	GATTCGATGGCCACGTTCTC
Shank3	NM_021423.4	AGGAACTTGCTTCCATTCGG	AGTCAGCATCTGCAATGTCC
Grip1	NM_028736.2	GACTGGAGCGAACAGAACAG	GTGTTAGTGGGTTCTCGTGTC
Ank2	NM_178655.3	TCTGAACCCAGCGTTTTGTC	TCTCCGTGTACCATGGTTGT
Homer1a	NM_011982.4	AATTTAAGGAAGCTGCTCGGC	CCTGTGAAGGGGTACTGGTC
Cacna1a	NM_007578.3	CCTGATGATGACAAGACACC	TTCCAGCCTCAAAACAGAAG
Cacna1b	NM_001042528.2	TTGAGTACCTCACTCGGGAC	GTCGTATTCAGCCCAGACTC
Cacna1e	NM_009782.3	CCTGACTCGAGATTCCTCCAT	ATGCTGCTCTGTCATATTCTGC
Cacna1d	NM_028981.3	TGTGATGTGCCAGTAGGTGA	CACGTATCGGGTTGGTCTTG
Cacna1h	NM_021415.4	CCTGGACCTCTTCATCACCT	GTACTTAAGGGCCTCGTCCA
Cacna2d3	NM_009785.1	ATCCTGAGGAGAATGCAAGAGAG	TTATGTCTCCTATGTCGCACCA
Cacnb2	NM_023116.4	TAAGCCCAGTGCAAACAGTG	CGCATGGAAGGTACCACATC
Scn2a	NM_001099298.3	TGTTTGATGTGAGCGTGGTC	CCAAGTCCCACGTTGTCAAA
Scn9a	NM_001290674.1	ACGGAGGTCTATGCCAAACT	ACCAACGCAAAAAGTAGCCA
Kcnn1	NM_001363407.1	CTTAACCGCGTCACCTTCAA	TATCGTGGTACCTCTCACACA
Kcnn2	NM_001312905.2	TTATCTTCGGCATGTTCGGC	AAGAATACAGCGACGCCTTG
Itgav	NM_008402.3	ATTGACGGGCCAATGAACTG	ATTCCACAGCCCAAAGTGTG
Itgb1	NM_010578.2	CTTATTGGCCTTGCCTTGCT	GATTTTCACCCGTGTCCCAC
Nlgn3	NM_172932.4	CCAACTTGGATATCGTCGCC	CATCTTCCGTGGGCACATAC
Nrxn1	NM_020252.3	TGACAGCAATTTGCCACTGA	CCTGTGTGTGTCTGGGGATA
Reln	NM_011261.2	TCGTCCTAGTAAGCACTCGC	GGAAGGGACACATTGTACGC
Cntnap2	NM_001004357.2	CATGGTGTACCAGACTTGCC	ATTGCTTACAGGGCTTTCCG
Arc	NM_018790.3	CCCCCAGCAGTGATTCATAC	GGTTTCATGCTGGCTTGTCT
Creb	NM_133828.2	ACAGGAGTCTGTGGATAGTGT	CCTGAGGCAGCTTGAACAAC
cFos	NM_010234	CAGAAGGGGCAAAGTAGAGC	TGATCTGTCTCCGCTTGGA
Dusp6	NM_026268.3	TTTCTTTCATAGATGAAGCCCGAG	GGGTCCTTTCGAAGTCAAGC
Egr1	NM_007913.5	GTCCTTTTCTGACATCGCTCTGA	CGAGTCGTTTGGCTGGGATA

Egr2	NM_010118.3	GCCAAGGCCGTAGACAAAAT	GTTGATCATGCCATCTCCCG
Npas4	NM_153553.5	ACCTGTCCCCAGAAGATCAC	CCCCTCCACTTCCATCTTCA
BDNF tot	NM_007540.4	ATTACCTGGATGCCGCAAA	TAATACTGTCACACACGCTCA
BDNF II	NM_001048139.1	GCCATCCACACGTGACAAAAC	TGCTGAATGGACTCTGCTCTC
BDNF IV	NM_001048141.1	CAGAGCAGCTGCCTTGATGTTT	CGCCTTCATGCAACCGAAGTAT
Fmr1	NM_008031.3	GGGTTGGACCTAACTCCTCT	TGATGAAACCACTAACACCCTC
Msra	NM_026322.4	GGTCAGCAGTCTATCCCACA	TGCTTTGAAAGAACCTTTTGGTATT
Braf	NM_139294.5	GGGCTGGTTTCCAAACAGAA	AATTCTCCATATCCCCCTGCT
Gm9758	NM_198666.4	AGTCAGAGGCTGGACATTGC	GCATCCTTCCTCCCCTCTCT
Gm11168	ENSMUST00000 178077	TCACATCCTAAAGTGTTGTGTATT	TGGCGAGAAACTGTAGGAAGA
Asmt	NM_001308488.2	CTTCACCGCCATCTACAGGTC	TGAAGGGCGAGAGGTCGAAG
Speer4cos	NR_001585.3	TAACACCGAAAACACCTCCTCA	TTTCTCTAACATCCTGCTGCACT
Actb	NM_007393.5	TTGCTGACAGGATGCAGAAG	AGTCCGCCTAGAAGCACTTG
Gapdh	NM_001289726.1	TGTGTCCGTCGTGGATCTGA	CCTGCTTCACCACCTTCTTGA
Hprt1	NM_013556.2	AAGCTTGCTGGTGAAAAGGA	TTGCGCTCATCTTAGGCTTT

**ChIP-qPCR primers (figure S5)**		**Forward sequence (5' → 3')**	**Reverse sequence (5' → 3')**
Itgb3 promoter		GAGTCCAGGAAGTGACCCAAA	AGGCTGAGTGTGATGGGTAAA
gRNA 2	Off-target #1	GGGGACACCCCTAGGAAAAT	GGCAAGATACGATGCCTTCC
	Off-target #2	GATTGGTTGTGCAATGATCGAG	CTGGAGACATCTGACGAGGA
	Off-target #3	TGCTGACTCCTCAAGGAACG	CCCCAAAGGTATCCTCGGTC
	Off-target #4	TGGGATTGTGTGGTGGGAAT	AACCCTCACCACTGTTCTCA
	Off-target #5	GTCTGTCCTGAGGTCTGGTG	TTCCACGGAAGTCCTAGCAT
	Off-target #6	TTCAGCACCGAACTCCGA	CCTAGGTGAGGAAGGACGG
	Off-target #7	CCTTTGAAGTGCCAACAGGA	GCTTCACCCATCCATGCC
	Off-target #8	AAAGGCTGCAGGAAAGAGTG	TTTCATGAGTGGGTGAGGGA
	Off-target #9	ATCATGAAGGCTGCGTGAAC	CCTGCTTACGTATGGGTGGA
	Off-target #10	GCCACTTGCATGGAGATACG	AGACGGTGGTGTGCTTCTAT
gRNA 3	Off-target #1	TTTGTAGAGCCCTGAGCCTG	AGCTCTCCAGGGATTAGCAC
	Off-target #2	AATAGGCCATGGAGTGGTCA	TTTCTGTGTGCGTCTGCATC
	Off-target #3	GACTCACTGAGACAAGCCCT	TGGTGTTCTCTTAGGCCAGT
	Off-target #4	ACTCCTCTGGGATGGAAGTC	CAATGAGGCAGGGAGAAGAC
	Off-target #5	GGGTGCTGTGTACAGGT	CAGGGCACACACACACC
	Off-target #6	TGCCAATAAGCAGCTGAACT	CAGACTGTGACTCTGTGGGA
	Off-target #7	CCCCAAAACCATATGAAAGGGG	GTGGCTCGACTTATGTTCCTTG
	Off-target #8	GATCAGCCCTCAGTGAGACA	TGAGCCCTAAGGAGACACAC
	Off-target #9	ATAGCTCCAGCTAAGGCTCG	ATCTGTCTTCACGTTGGCAG
	Off-target #10	ACGTCAAGTAATTGGGTAGGC	AAGCTCTTGTCATCACCGTAG
