# Supplementary material for: CRISPR-mediated activation of autism gene Itgb3 restores cortical network excitability via mGluR5 signaling
Source: Mol Ther Nucleic Acids. 2022 Jul 20;29:462–80. doi: 10.1016/j.omtn.2022.07.013 (PMC9382421; doi:10.1016/j.omtn.2022.07.013)
Supplement: Document S2. Article plus supplemental information [file mmc2.pdf]

Molecular Therapy
Nucleic Acids
Original Article

CRISPR-mediated activation of autism gene *Itgb3* restores cortical network excitability via mGluR5 signaling

Fanny Jaudon,[1,2] Agnes Thalhammer,[2,3] Lorena Zentilin,[4] and Lorenzo A. Cingolani[1,2,5]

[1]Center for Synaptic Neuroscience and Technology (NSYN), Fondazione Istituto Italiano di Tecnologia (IIT), 16132 Genoa, Italy; [2]Department of Life Sciences, University of Trieste, 34127 Trieste, Italy; [3]IRCCS Ospedale Policlinico San Martino, 16132 Genoa, Italy; [4]AAV Vector Unit, International Centre for Genetic Engineering and Biotechnology (ICGEB), 34149 Trieste, Italy

Many mutations in autism spectrum disorder (ASD) affect a single allele, indicating a key role for gene dosage in ASD susceptibility. Recently, haplo-insufficiency of *ITGB3*, the gene encoding the extracellular matrix receptor β3 integrin, was associated with ASD. Accordingly, *Itgb3* knockout (KO) mice exhibit autism-like phenotypes. The pathophysiological mechanisms of *Itgb3* remain, however, unknown, and the potential of targeting this gene for developing ASD therapies uninvestigated. By combining molecular, biochemical, imaging, and pharmacological analyses, we establish that *Itgb3* haplo-insufficiency impairs cortical network excitability by promoting extra-synaptic over synaptic signaling of the metabotropic glutamate receptor mGluR5, which is similarly dysregulated in fragile X syndrome, the most frequent monogenic form of ASD. To assess the therapeutic potential of regulating *Itgb3* gene dosage, we implemented CRISPR activation and compared its efficacy with that of a pharmacological rescue strategy for fragile X syndrome. Correction of neuronal *Itgb3* haplo-insufficiency by CRISPR activation rebalanced network excitability as effectively as blockade of mGluR5 with the selective antagonist MPEP. Our findings reveal an unexpected functional interaction between two ASD genes, thereby validating the pathogenicity of *ITGB3* haplo-insufficiency. Further, they pave the way for exploiting CRISPR activation as gene therapy for normalizing gene dosage and network excitability in ASD.

INTRODUCTION

Alterations in gene dosage are important contributing factors to autism spectrum disorder (ASD), as highlighted by the high frequency of copy-number variations and mutations affecting a single allele in patients with ASD.[1–6] In particular, recent genetic screens have identified patients with ASD with rare missense and protein-truncating mutations in *ITGB3*, the gene encoding the extracellular matrix (ECM) receptor β3 integrin.[7–10] Together with previous genetic studies,[11,12] these findings indicate that *ITGB3* haplo-insufficiency predisposes to ASD.[13] Accordingly, *Itgb3* knockout (KO) mice exhibit increased grooming in novel environments and deficits in social memory,[14] two features that recapitulate the repetitive behaviors and abnormal social interactions of patients with ASD.[15,16]

Integrins are cell adhesion molecules (CAMs) that bind to ECM proteins and counter-receptors on adjacent cells.[17,18] Their overactivation is involved in diverse pathologies, including cancer and thrombosis, and thus they represent a target of choice for developing drug therapies based on pharmacological inhibitors.[19] In the brain, integrins regulate synaptic connectivity and plasticity in response to chemical and mechanical cues.[13,16,20–23] The two most abundant neuronal integrins are β1 and β3, which have non-overlapping functions. While inhibition of β1 integrin compromises basal synaptic transmission and stabilization of long-term potentiation (LTP),[24–28] full, but not partial, loss of β3 integrin impairs homeostatic plasticity, a form of plasticity that stabilizes network output in the face of external perturbations.[29–31] Although the identification of highly selective roles for integrins in neurons suggests their potential as therapeutic targets also for brain disorders, the pathophysiological mechanisms of *Itgb3* haplo-insufficiency remain unclear and the possibility of targeting this integrin for developing ASD therapies unexplored.

To address these questions, we have established high-throughput cellular systems to assess how changes in *Itgb3* gene dosage affect synaptic ASD signaling pathways and cortical excitability, which is consistently dysfunctional in ASD cellular models.[13,32,33] We find that *Itgb3* haplo-insufficiency impairs network excitability and synchrony by limiting synaptic signaling of the metabotropic glutamate receptor mGluR5, which is similarly disrupted in fragile X syndrome (FXS), the most frequent monogenic form of ASD.[34–37] To assess the therapeutic potential of targeting *Itgb3*, we have implemented neuronal CRISPR activation (CRISPRa) systems that, unlike overexpression of exogenous β3 integrin, restored precisely β3 integrin protein levels to wild-type (WT) values both *in vitro* and *in vivo*. Targeted activation of *Itgb3* rescued mGluR5 signaling and cortical excitability

Received 28 July 2021; accepted 15 July 2022;
https://doi.org/10.1016/j.omtn.2022.07.013.

[5]Lead contact

Correspondence: Lorenzo A. Cingolani, Department of Life Sciences, University of Trieste, 34127 Trieste, Italy.
E-mail: lcingolani@units.it

 © 2022 The Author(s).
This is an open access article under the CC BY license (http://creativecommons.org/licenses/by/4.0/).

as effectively as pharmacological blockade of mGluR5 with the selective inverse agonist MPEP, an established FXS rescue strategy.[34] Altogether, our findings reveal that synaptic integrins shape core ASD signaling pathways and, in turn, circuit dynamics. Further, they indicate that CRISPRa-based gene therapies are ideally suited to normalizing gene dosage and network excitability in ASD.

RESULTS

Itgb3 haplo-insufficiency impairs network excitability

To assess whether neuronal β3 integrin regulates network excitability, we performed multi-electrode array (MEA) recordings in WT, *Itgb3*$^{+/-}$ (Heterozygous; Het), and *Itgb3*$^{-/-}$ (KO) primary cortical neurons cultured in the absence of glial cells. While qRT-PCR, western blots, and confocal imaging showed that mRNA and protein levels for β3 integrin were decreased by ~50% and ~100% in Het and KO neurons, respectively (Figures S1A–S1D), MEA recordings indicated that firing rate, burst rate, and percentage of spikes in burst were reduced to the same extent in Het and KO networks (40%–50%; Figures 1A–1E).

To monitor network activity at the level of individual neurons, we used a red-shifted, genetically encoded Ca^{2+} indicator, jRCaMP1b,[38] whose spontaneous somatic fluorescence transients correlate with firing activity.[39] While the amplitude of jRCaMP1b signals was not different between the three genotypes, their frequency was decreased by ~38% in both Het and KO neurons (Figures 1F–1I). Thus, a 50% reduction in β3 integrin expression, as observed in Het neurons, is as effective as a complete ablation of this gene in compromising network excitability.

Itgb3 haplo-insufficiency promotes membrane expression of mGluR5

To gain molecular insight into how β3 integrin regulates network dynamics, we screened WT, Het, and KO neurons for differences in the expression of 48 transcripts, including those for synaptic proteins, ion channels, and CAMs. We found that the expression of six genes (*Grm1*, *Grm5*, *Homer1a*, *Fmr1*, *Cacnb2*, and *Nlgn3*), all of which have been implicated in ASD (http://gene.sfari.org), was reduced in both Het and KO neurons (Figure S2A).

Because the group I metabotropic glutamate receptors mGluR1 (*Grm1*) and mGluR5 (*Grm5*) cooperate with Homer1a to regulate excitatory synaptic transmission and intrinsic excitability,[40–43] we selected these three genes for further analyses. Although changes in mRNA abundance can affect protein expression, correlation between transcript and protein levels is generally low.[44] We therefore assessed Homer1a, mGluR1, and mGluR5 protein expression in membrane fractions. Surprisingly, protein levels for Homer1a and mGluR1 were unchanged, while those for mGluR5 were 43% higher, in Het neurons. In line with previous findings,[29,45] all AMPA-type glutamate receptors (AMPARs) were instead reduced by 68%–75% (Figure 2). Hence, *Itgb3* haplo-insufficiency promotes membrane expression of the metabotropic glutamate receptor mGluR5.

Itgb3 haplo-insufficiency favors functional expression of mGluR5

Several studies have investigated how integrins regulate ionotropic glutamate receptors.[25,30,45–50] By contrast, a crosstalk between integrins and mGluRs has never been described despite both being key players in shaping excitatory synaptic transmission and intrinsic excitability.[40,41,43]

To test whether *Itgb3* haplo-insufficiency favors functional expression of mGluR5, we investigated the relative contribution of mGluR1 and mGluR5 to network excitability by activating them with the selective group I mGluR agonist (S)-3,5-dihydroxyphenylglycine (DHPG) in the presence or absence of mGluR1 (Bay 36-7620; Bay) and/or mGluR5 (2-methyl-6-(phenylethynyl)-pyridine [MPEP]) antagonists. In the hippocampus, pharmacological stimulation of group I mGluRs induces synchronized discharges, mainly because activation of these receptors elevates intrinsic excitability.[40,51,52] Accordingly, DHPG increased firing rate (≥38%; Figure 3C), burst rate (≥73%; Figure 3D), and burst synchrony (Figures 3H and 3I) in both WT and *Itgb3* Het cultures. These effects were due to activation of group I mGluRs because they were blocked by a co-application of Bay and MPEP (Figure 3). Previous work has shown that mGluR1 is more effective than mGluR5 in supporting hippocampal excitability.[53,54] This is the case also for WT cortical networks where the mGluR1 blocker Bay, but not the mGluR5 blocker MPEP, prevented the DHPG effects as effectively as a co-application of the two blockers (Figures 3C, 3D, 3H, and 3I). These results indicate that mGluR1 is both necessary and sufficient for increasing network excitability and synchrony in WT cortical circuits.

By contrast, neither Bay nor MPEP, when applied alone, prevented the effects of DHPG in *Itgb3* Het cultures (Figures 3C, 3D, 3H, and 3I), indicating that both mGluR1 and mGluR5 are sufficient but neither one is necessary for supporting firing activity of β3-integrin-deficient neurons (Figure 3G).

β3 integrin interacts with and regulates synaptic localization of mGluR5

The reduction in burst duration induced by DHPG selectively in Het networks (Figure 3F) further indicated that group I mGluR signaling is anomalous in β3-integrin-deficient neurons. Burst duration can be affected by the level of synaptic localization of group I mGluRs.[55,56] We therefore used the GABAergic blocker bicuculline to elevate network excitability, thus promoting activation of synaptic group I mGluRs by synaptically released glutamate.[51,55] Bicuculline boosted network activity largely independently of group I mGluR signaling (Figures 4A–4F). In the presence of Bay, when only synaptic mGluR5 could be activated, the bicuculline-dependent increase in firing rate was nonetheless lower in *Itgb3* Het than WT cultures (Figure 4C; 32% versus 75% increase).

Interestingly, these effects mirrored those obtained upon pharmacological stimulation of mGluR5, which was effective in increasing firing rate only in Het networks (Figure 3C). This might be because β3

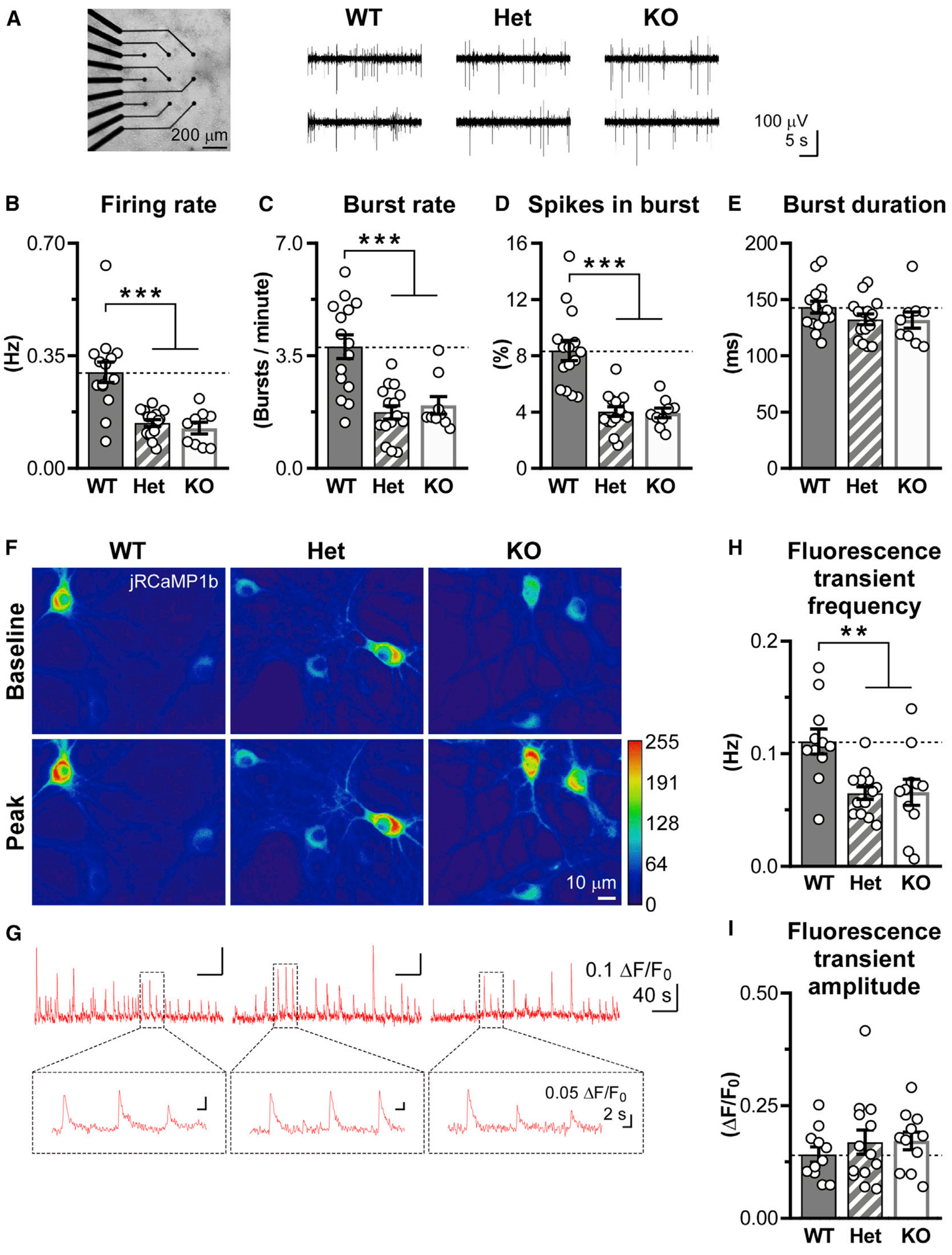

Figure 1. Network activity is reduced in both *Itgb3* Het and KO cortical neurons

(A) Left, cortical neurons plated on MEAs. Right, representative traces from two electrodes for each genotype. (B–E) Quantification of experiments as in (A), showing that firing rate, burst rate, and percentage of spikes in burst are reduced in both *Itgb3* Het and KO cortical neurons (***$p < 0.001$, one-way ANOVA followed by Tukey's post-test, n = 9–15 recordings from 5 independent cultures). (F) Representative jRCaMP1b fluorescence transients in response to spontaneous network activity in primary cortical neurons. Images are average of 6 consecutive frames during baseline (top) and at the peak of the largest transient (bottom). (G) Spontaneous somatic jRCaMP1b responses over 5 min. Inset, higher magnification, showing good signal-to-noise ratio. (H and I) Quantification of experiments as in (F) and (G) for frequency and amplitude. The frequency of spontaneous fluorescence transients is reduced in both *Itgb3* Het and KO cortical neurons (**$p < 0.01$, one-way ANOVA followed by Tukey's post-test; n = 11–13 fields of view from 7–8 independent cultures). Data are presented as mean ± SEM; dots represent individual recordings. See also Figure S1.

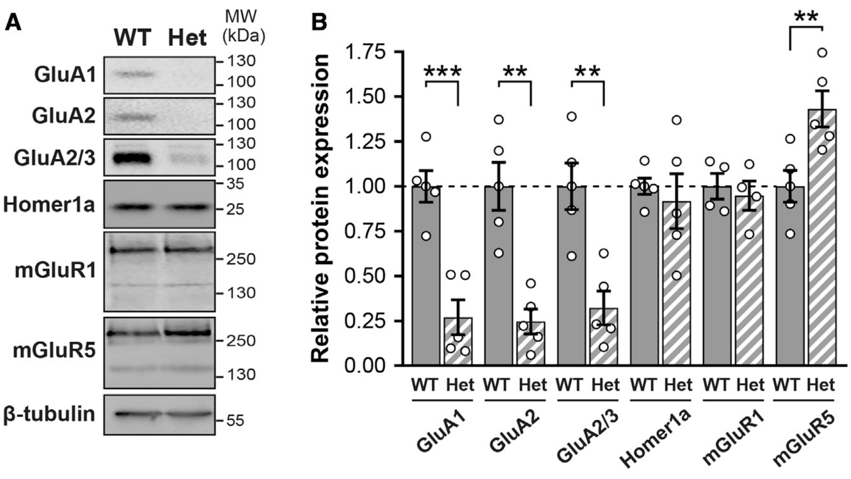

Figure 2. Regulation of glutamate receptor expression by β3 integrin

(A) Representative western blots of membrane-enriched fractions from WT and *Itgb3* Het cortical neurons. (B) Quantification of experiments as in (A) showing that levels of AMPARs are reduced while those of mGluR5 are increased in Het neurons (**$p < 0.01$, ***$p < 0.001$, unpaired Student's t test; n = 4–5 independent cultures). Data are shown as mean ± SEM; dots represent individual values. See also Figure S2.

integrin is required for the synaptic localization of mGluR5 (Figure 4G); a requirement that cannot be compensated for by an overall increase in mGluR5 functional expression (Figures 2 and 3). To test this hypothesis, we used confocal microscopy to compare mGluR5 levels in dendritic spines, as identified by vGlut1 signal, and adjacent dendritic shafts (Figures 4H and 4I). The relative difference ((S−D)/(S+D); S, synaptic signal; D, dendritic signal), which ranges from −1 (maximal dendritic localization) to +1 (maximal synaptic localization), revealed that mGluR5 was equally distributed between dendritic spines and dendritic shafts in WT neurons but was depleted from dendritic spines of *Itgb3* Het neurons (Figure 4J). Given these results, we tested whether β3 integrin and mGluR5 were present in the same signaling complexes. Indeed, they co-immunoprecipitated from cortical tissue (Figure 4K). Taken together, these results indicate that β3 integrin interacts with and, in turn, regulates functional expression of synaptic mGluR5.

Normalization of neuronal β3 integrin expression by CRISPRa

We next used CRISPRa to enhance transcription of β3 integrin as a means to normalize mGluR5 expression and network excitability. To this end, we employed a nuclease deficient Cas9 (dCas9) from *S. pyogenes* fused to the transcriptional activator VP64.[57–63] We designed three gRNAs targeting the *Itgb3* promoter (Figure S3A). As quantified by qRT-PCR, all gRNAs increased β3 integrin expression by 3- to 4-fold in N2a cells, with gRNA 3 being the most effective (Figures S3B and S3C). While co-expressing gRNAs 1 and 3 had no additive effect, likely because of a steric hindrance (Figure S3A), combining gRNAs 2 and 3 enhanced β3 integrin expression by 8-fold (Figure S3C). As functional readout, we performed a cell attachment assay and found that CRISPRa strengthened adhesion of N2a cells to fibronectin, a β3 integrin ligand (Figures S3D and S3E).

We next infected primary cortical neurons with lentiviruses expressing a gRNA together with dCas9-VP64 and EGFP (Figure S4A). gRNA 3 enhanced *Itgb3* mRNA levels in WT and Het (by 2- to 2.5-fold) but not KO neurons, where both copies of the target gene are missing. As in N2a cells, combining gRNAs 2 with 3 led to larger effects in both WT and Het (3- to 4.5-fold increments; Figure S4B). Notably, exogenous expression of β3 integrin using a lentivirus with a Synapsin promoter[31] resulted in an excessive increase in the amount of β3 integrin mRNA (∼200-fold; Figure S4B). Western blots indicated that changes in β3 integrin protein levels paralleled those of the transcript (Figures S4C and S4D). In summary, CRISPRa, but not overexpression, can be used to normalize neuronal expression of β3 integrin.

CRISPRa is not as prone as CRISPR-Cas9 to off-target effects because it does not cleave chromosomal DNA; spurious activation of off-target genes would require dCas9-VP64 to bind consistently to a promoter region.[64,65] Indeed, previous studies have failed to identify significant off targets for CRISPRa in neurons.[59,60] Although none of the predicted off targets for gRNAs 2 and 3 were on a promoter region (Figure S5A), we used chromatin immunoprecipitation followed by qPCR (ChIP-qPCR) to evaluate binding of dCas9 to the top ten predicted off targets for each gRNA (Figures S5B and S5C). Relative to gRNA control (Ctrl), gRNAs 2 and 3 induced a 25-fold enrichment of dCas9 at the *Itgb3* promoter (Figures S5C), confirming binding to the target site. A small enrichment of dCas9 was detected for two predicted off targets (#9 of gRNA 2 and #6 of gRNA 3; Figure S5C). As quantified by qRT-PCR, this had, however, no effect on the expression of the genes at these loci (*Msra* and *Braf*; Figure S5D).

β3 integrin is expressed in dendrites in apposition to synaptic markers.[30,45,49] As quantified by confocal microscopy, CRISPRa rescued the reduced expression of β3 integrin in soma and dendrites of Het neurons to WT values (Figures S4E–S4G) while having no effects in KO neurons (Figure S1E). Size and density of dendritic β3 integrin clusters were largely preserved (Figures S4E and S4G). By contrast, expression of exogenous β3 integrin led to widespread and ectopic localization of this protein (Figures S4E–S4G). None of the experimental conditions affected expression of and co-localization with the synaptic marker vGlut1 (Figures S4H and S4I). In summary, β3 integrin maintains its expression pattern when the activity of its endogenous promoter is enhanced by CRISPRa.

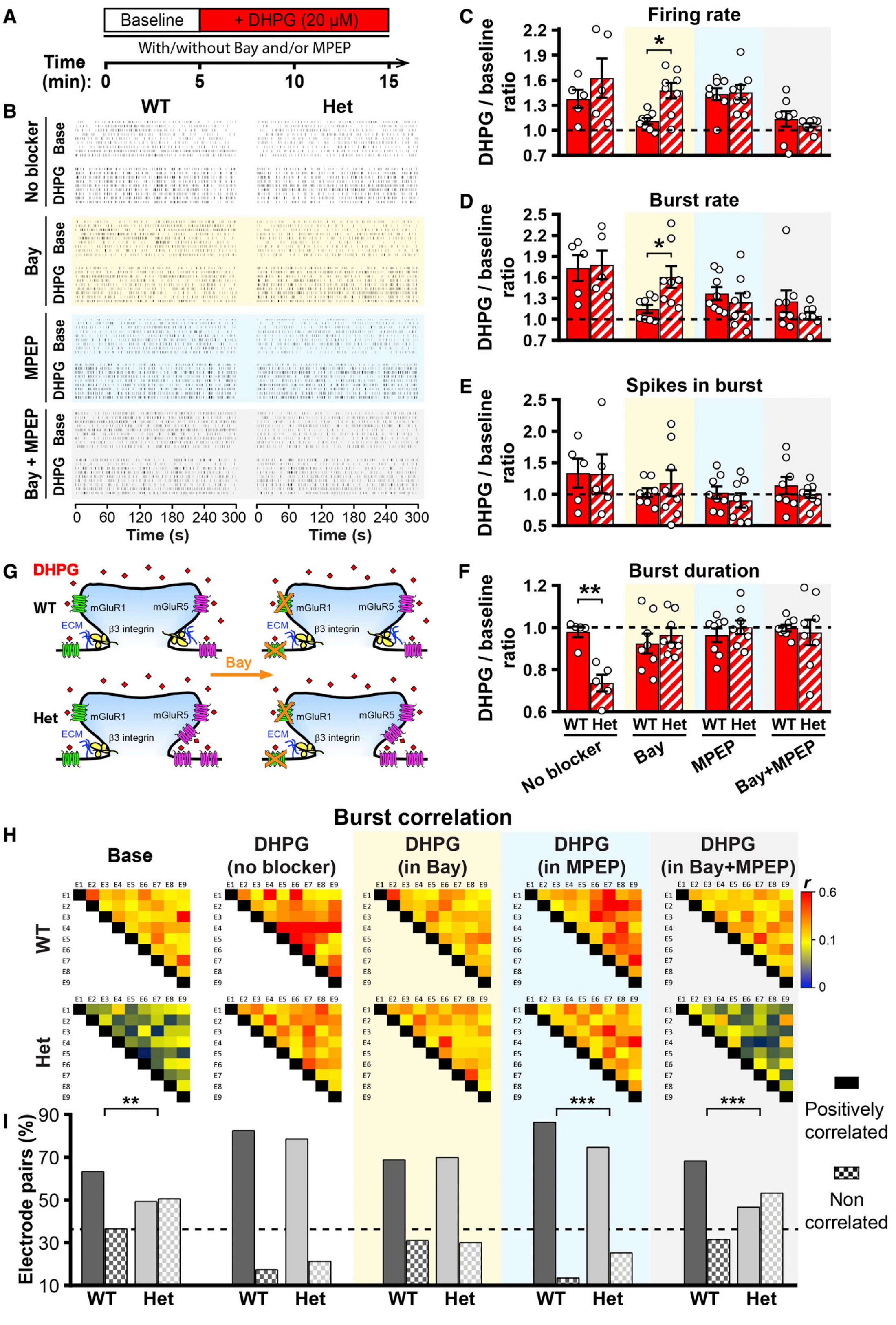

(legend on next page)

Rebalancing β3 integrin levels normalizes mGluR5 expression and network excitability

We first addressed whether rebalancing β3 integrin levels with CRISPRa is effective at normalizing mGluR5 expression. In *Itgb3* Het neurons, CRISPRa brought both mRNA (Figure S2A) and protein levels (Figures 5A and 5B) of mGluR5 back to WT values. By contrast, overexpressing β3 integrin nearly abolished mGluR5 expression (Figures 5A and 5B).

We next assessed, by MEA recordings, how enhancing neuronal expression of β3 integrin regulates circuit dynamics. While CRISPRa was effective in restoring excitability and synchrony of Het neurons to WT values, exogenous expression of β3 integrin induced oversynchronous bursting (Figures 5C–5I). Importantly, CRISPRa acted specifically via *Itgb3* because it had no effect in KO neurons (Figure S6).

Since changes in inhibitory synaptic strength can contribute to differences in network dynamics, we evaluated the effects of β3 integrin in the presence of bicuculline, a $GABA_A$ receptor blocker. Disinhibition promoted firing and burst rate, though to a similar extent in most experimental conditions (Figure S7; see also Figure 4). In neurons expressing exogenous β3 integrin, some increments were not as pronounced (Figure S7), possibly because of a ceiling effect. Altogether, these data suggest that β3 integrin supports network activity irrespective of synaptic inhibition.

To monitor circuit defects and their rescue at the level of individual neurons, we resorted to jRCaMP1b. Somatic fluorescence transients exhibited differences in frequency between the various conditions analogous to those found for network firing rates (Figures 6A and 6B versus Figures 5C and 5D). Unlike MEA recordings, fluorescence signals can be interpreted also in terms of their amplitude. Mean fluorescence transients were of a similar size across conditions (Figures 6C and S8B), whereas individual transients were highly variable in amplitude within each recording (Figures 1G and 6A), suggesting variability of the underlying firing activity.[39]

Because a positive correlation in the amplitude profiles between two neurons is indicative of similar firing rates (Figure S8D), we compared the fluorescence amplitude profiles of all the neurons within a field of view. 51% and 31% of neuron pairs were positively correlated in WT and Het networks, respectively. In Het, CRISPRa could restore the percentage of positively correlated neuron pairs to WT values, while exogenous β3 integrin increased positive correlation far above WT levels (Figures 6D, 6E, S8C, and S8E). These data suggest that neuronal synchronization, which is abnormal in many models of ASD,[33] is critically dependent on *Itgb3*.

Chronic blockade of mGluR5 rescues impaired network function of *Itgb3* Het neurons

Our data indicate that *Itgb3* haplo-insufficiency reduces network excitability (Figure 1) and enhances functional expression of extra-synaptic mGluR5 (Figures 2, 3, and 4). Further, rebalancing β3 integrin levels with CRISPRa effectively normalizes both network activity and mGluR5 expression (Figures 5 and 6).

To test whether it is enhanced extra-synaptic mGluR5 signaling that compromises the excitability of *Itgb3* Het neurons, we employed the inverse agonists Bay and MPEP, which block constitutive agonist-independent activity of mGluR1 and mGluR5, respectively. Chronic co-application of Bay and MPEP is known to potentiate excitatory synaptic transmission in cortical neurons.[42] Accordingly, when used together, Bay and MPEP increased firing rate (≥20% at 1–4 h), burst rate (≥15% at 2–4 h), and network synchrony of both WT and Het neurons (Figure 7). Compellingly, chronic treatment with MPEP alone was sufficient to increase excitability (Figures 7B–7D) and synchrony (Figures 7H and 7I) of *Itgb3* Het, but not WT, circuits.

Altogether, these findings indicate that heightened mGluR5 activation in β3-integrin-deficient neurons impairs network excitability and synchrony. Further, they suggest that circuit defects caused by *Itgb3* haplo-insufficiency can be rescued by either CRISPR-mediated activation of *Itgb3* or chronic pharmacological blockade of mGluR5.

CRISPRa normalizes β3 integrin expression *in vivo*

In the brain, β3 integrin is enriched in deep-layer cortical pyramidal neurons.[13,66,67] To explore the translational potential of modulating β3 integrin expression, we therefore expressed the CRISPRa machinery *in vivo* in these neurons. To this end, we crossed the *Itgb3* mouse line with CaMKIIa-Cre knockin mice, which express the recombinase Cre in pyramidal neurons of the forebrain. Deep-layer cortical pyramidal neurons can be efficiently targeted in these mice with intraventricular injections at postnatal day 0 (P0) of Cre-dependent recombinant adeno-associated viruses (rAAVs; AAV-PHP.eB serotype; Figures 8A and 8B).

Figure 3. *Itgb3* haplo-insufficiency boosts overall activation of mGluR5

(A) Timeline for application of the group I mGluR agonist DHPG (20 μM) in the presence or absence of mGluR1 (Bay; 10 μM) and/or mGluR5 (MPEP; 5 μM) inverse agonists. Bay and MPEP application started 30 min before DHPG. (B) Representative raster plots of network activity from WT and Het cultures for the indicated conditions. (C–F) Quantification for experiments as in (A) and (B). Values are normalized to baseline for each recording. Selective activation of mGluR5 (Bay condition; yellow background) elevates firing and burst rate only in Het cultures (*p < 0.05, **p < 0.01, two-way ANOVA followed by Bonferroni post-test; n = 5–8 for each condition from 5 independent cultures). Data are presented as mean ± SEM; dots represent individual values. (G) Scheme showing that DHPG activates only mGluR5 in the presence of the mGluR1 antagonist Bay. (H) Representative heatmaps of Pearson's correlation coefficients (*r*) for burst activity from wells containing 9 electrodes (E1–E9). Selective activation of mGluR5 (DHPG in Bay condition; yellow background) rescues network synchrony of Het cultures to WT values. (I) Quantification of experiment as in (H). All electrode pairs exhibited a positive *r*. The graph shows the percentage of *r* with a p <0.05 (positively correlated) and a p >0.05 (non-correlated; **p < 0.01, ***p < 0.001, chi-square test; n = 164–272 pairs).

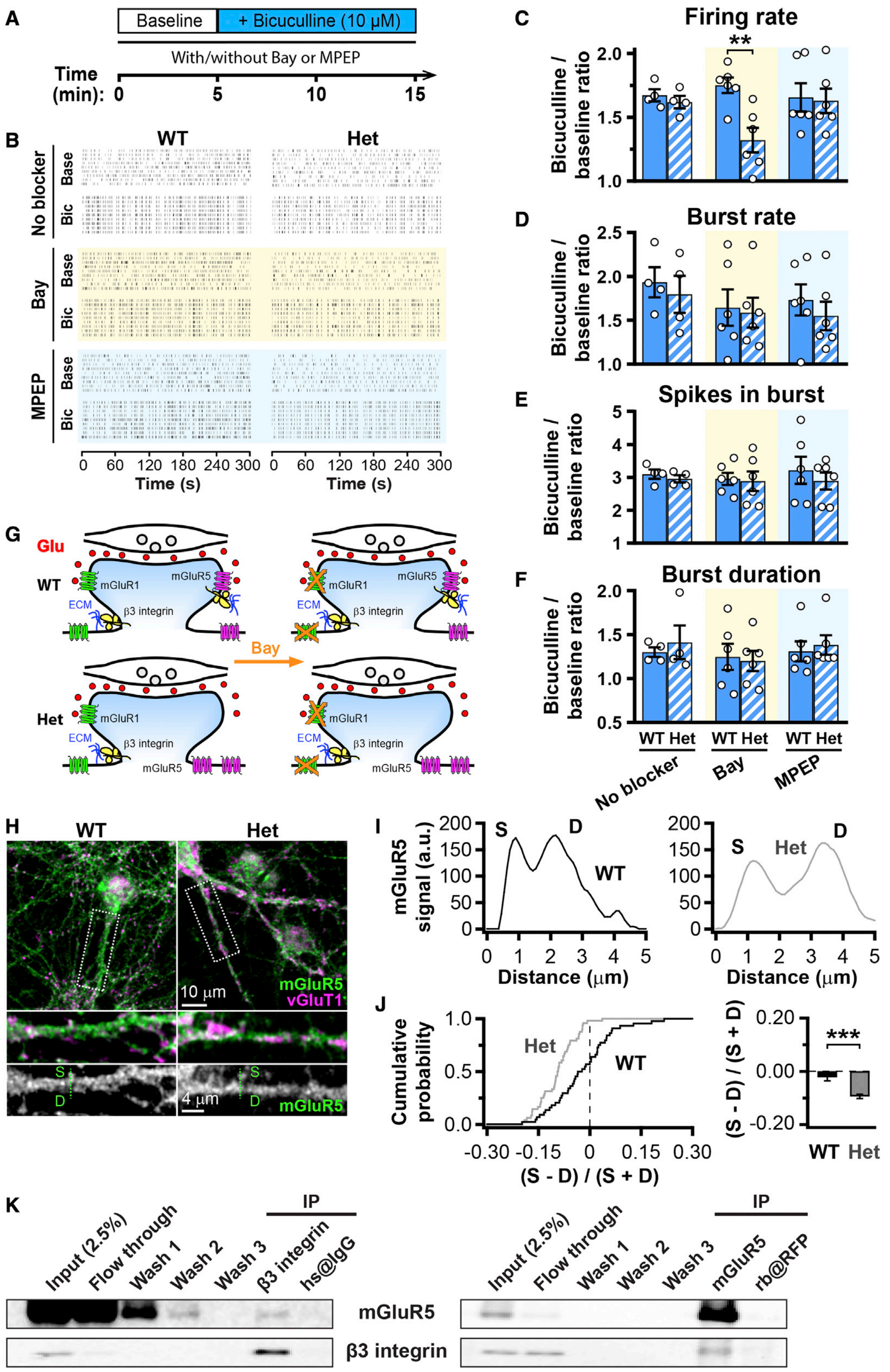

(legend on next page)

Because of the reduced packaging capacity of rAAVs, we used dCas9 from *S. aureus* (Sa-dCas9), which is smaller than the canonical dCas9 from *S. pyogenes*, while exhibiting improved specificity.[68] We co-injected two rAAVs: the first (pAAV-Syn-DIO-Sa-dCas9-VPR) to deliver Sa-dCas9 fused to the transcriptional activator VPR, while the second (pAAV-U6-gRNA-Syn-DIO-EGFP) to deliver a gRNA (Ctrl or active) and EGFP (Figure 8A). As *S. aureus* Cas9 uses a different protospacer adjacent motif (PAM), we generated three new gRNAs targeting the *Itgb3* promoter and tested their efficiency in N2a cells (Figures S9A and S9B). From these experiments, we selected gRNAs 4 and 5, the combination of which resulted in a 7.2-fold increase in β3 integrin expression (Figure S9B).

In vivo CRISPRa treatment with these gRNAs restored cortical β3 integrin expression of *Itgb3* Het mice to WT values at both mRNA and protein levels (Figures 8C and 8D). To assess CRISPRa specificity *in vivo*, we performed a genome-wide ChIP-seq analysis and compared the peaks identified in the gRNA Ctrl and gRNA 4 + 5 conditions. While we observed on-target binding at the *Itgb3* promoter (Figures S9C and S9D), we did not detect any additional target significantly enriched in the gRNA 4 + 5 conditions (Figure S9E). We nevertheless checked the expression level of the four genes (*Gm9758*, *Gm11168*, *Asmt*, and *Speer4cos*) with a peak within 1 kb of their transcription start site (TSS) and found no difference in gene expression (Figure S9F), confirming the specificity of CRISPRa *in vivo*.

Taken together, these results indicate that CRISPRa can be used to normalize *Itgb3* expression in cortical pyramidal neurons *in vivo*.

DISCUSSION

Our study reveals an unexpected function for the ASD gene *Itgb3* in regulating synaptic signaling of mGluR5 with a direct impact on neuronal excitability. Further, it indicates CRISPR-mediated activation of *Itgb3* as suitable gene therapy to normalize network defects in ASD. First, by using MEA recordings and Ca^{2+} imaging of populations of neurons, we found that *Itgb3* haplo-insufficiency impairs activity and synchrony of cortical networks (Figure 1). Second, by combining biochemical, imaging, and pharmacological analyses, we demonstrated that reduced levels of neuronal β3 integrin favor extra-synaptic over synaptic mGluR5 signaling (Figures 2, 3, and 4). Third, we implemented CRISPRa to control *Itgb3* expression *in vitro* and *in vivo* (Figures 8 and S4). Using this approach, we established that it is necessary to restore WT gene dosage of *Itgb3* in order to rescue mGluR5 expression and neuronal excitability (Figures 5 and 6). Fourth, we determined that pharmacological blockade of mGluR5 with the selective inverse agonist MPEP rescues circuit defects of *Itgb3* Het neurons (Figure 7), thereby mechanistically linking molecular and cellular deficits of *Itgb3* haplo-insufficiency.

Multiple cues from ECM and glial cells regulate circuit dynamics. For example, enzymatic digestion of ECM components induces epileptiform activity in primary neurons.[69–71] Integrins on the neuronal surface are ideally positioned to adjust neuronal excitability in response to changes in the extracellular environment. In particular, the glial factors TNFα and SPARC, which are down- and up-regulated by action-potential firing, increase and decrease β3 integrin expression, respectively.[30,48,72] This integrin is therefore negatively regulated by neuronal activity via glia-released factors. Because we find here that elevating β3 integrin expression boosts, in turn, cortical excitability via mGluR5 signaling, we propose the existence of a negative feedback loop between neuronal β3 integrin and the excitability of the network.

Both MEA recordings and Ca^{2+} imaging indicated that β3 integrin promotes network synchrony (Figures 5H, 5I, 6D, and 6E). Several factors determine network dynamics, such as the number of shared connections, the excitatory/inhibitory ratio, and the level of intrinsic excitability.[41,73,74] β3 integrin positively modulates membrane expression of AMPARs (Figure 2) and excitatory synaptic strength,[29,30,45] though not the overall number of excitatory synaptic connections (Figure S4I). By contrast, the role of this integrin in setting the strength of inhibitory synaptic coupling is likely minor because changes in its expression had no effect on the degree of disinhibition (Figure S7) or the expression of GABA receptors (Figure S2). In addition to those for AMPARs, β3 integrin regulated other neuronal genes (Figure S2), many of which are involved in ASD. Although some of these effects could be secondary to changes in network activity, they clearly indicate that a mere 50% deficiency in one ASD gene affects the expression of many others. Five of them (*Grm1*, *Grm5*, *Homer1a*, *Fmr1*, and *Nlgn3*) drew our attention as potentially more tightly linked to integrin signaling in ASD because their expression was diminished in both *Itgb3* Het and KO neurons and could be rescued to WT values by CRISPRa (Figure S2).

A closer analysis of two of them (*Grm1 and Grm5*; mGluR1 and mGluR5) indicated that β3 integrin, which is localized at excitatory

Figure 4. *Itgb3* haplo-insufficiency limits synaptic activation of mGluR5
(A) Timeline for bicuculline application (10 μM) in the presence or absence of mGluR1 (Bay; 10 μM) or mGluR5 (MPEP; 5 μM) inverse agonists. Bay and MPEP application started 30 min before Bicuculline. (B) Representative raster plots of network activity from WT and Het cultures for the indicated conditions. (C–F) Quantification for experiments as in (A) and (B). Values are normalized to baseline for each recording. If only mGluR5 can be activated (Bay condition; yellow background), bicuculline is less effective in elevating firing rate in Het cultures (**$p < 0.01$, two-way ANOVA followed by Bonferroni post-test; n = 4–6 for each condition from 3 independent cultures). Data are presented as mean ± SEM; dots represent individual values. (G) Scheme showing that synaptically released glutamate (Glu) activates only synaptic mGluR5 in the presence of the mGluR1 antagonist Bay. (H) Representative confocal images of primary cortical neurons from WT and Het cultures stained for mGluR5 and the presynaptic marker vGlut1. (I) Line profiles of mGluR5 intensity for WT (left, black) and Het (right, gray) from the green dotted lines in H (S, dendritic spine; D, dendritic shaft). (J) Quantification of the distribution of mGluR5 between vGlut1-positive dendritic spines and adjacent dendritic shafts for experiments as in (H). Left, cumulative distribution of the relative difference ((S−D)/(S+D); S, synaptic signal; D, dendritic signal) for each spine/dendritic shaft pair (n = 44–48 pairs). Right, same data expressed as mean ± SEM (***$p < 0.001$, unpaired two-tailed Student's t test). mGluR5 signal is weaker in dendritic spines of *Itgb3* Het neurons. (K) Co-immunoprecipitation experiments from brain cortical extracts, showing that β3 integrin co-precipitates mGluR5 (left) and mGluR5 co-precipitates β3 integrin (right).

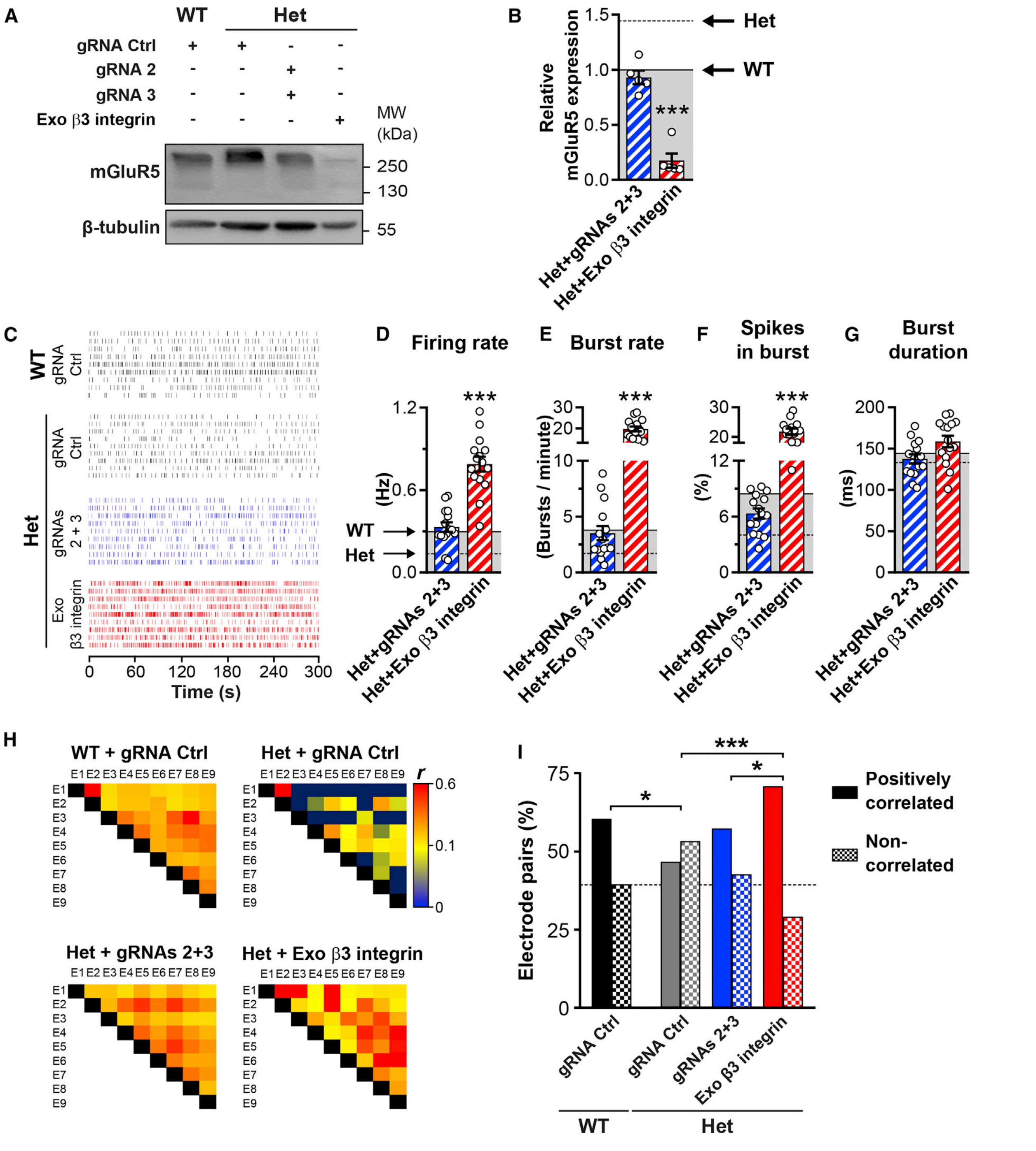

Figure 5. CRISPRa normalizes mGluR5 expression and firing activity in *Itgb3* Het cortical networks

(A) Representative western blots of membrane-enriched fractions from WT and *Itgb3* Het cortical neurons expressing the indicated constructs. (B) Quantification of experiments as in (A) showing that CRISPRa restores mGluR5 expression levels in Het neurons to WT values (***p < 0.001 relative to WT, one-way ANOVA followed by Tukey's post-test; n = 5 independent cultures). (C) Representative raster plots of network activity in MEA experiments for WT and *Itgb3* Het cortical neurons expressing the indicated constructs. (D–G) Quantification of experiments as in (C). Unlike exogenous expression of β3 integrin, CRISPRa normalizes cortical network activity in Het cultures (***p < 0.001 relative to WT, one-way ANOVA followed by Tukey's post-test, n = 15 each from 5 independent cultures). Data are presented as mean ± SEM; dots represent individual values. (H) Representative heatmaps of Pearson's correlation coefficients (*r*) for burst activity from wells containing 9 electrodes (E1–E9). Network synchrony correlates with β3 integrin levels. (I) Quantification of experiment as in (H). All electrode pairs exhibited a positive *r*. The graph shows the percentage of *r* with a p <0.05 (positively correlated) and a p >0.05 (non-correlated; *p < 0.05, ***p < 0.001, chi-square test; n = 447–532 pairs). See also Figures S6 and S7.

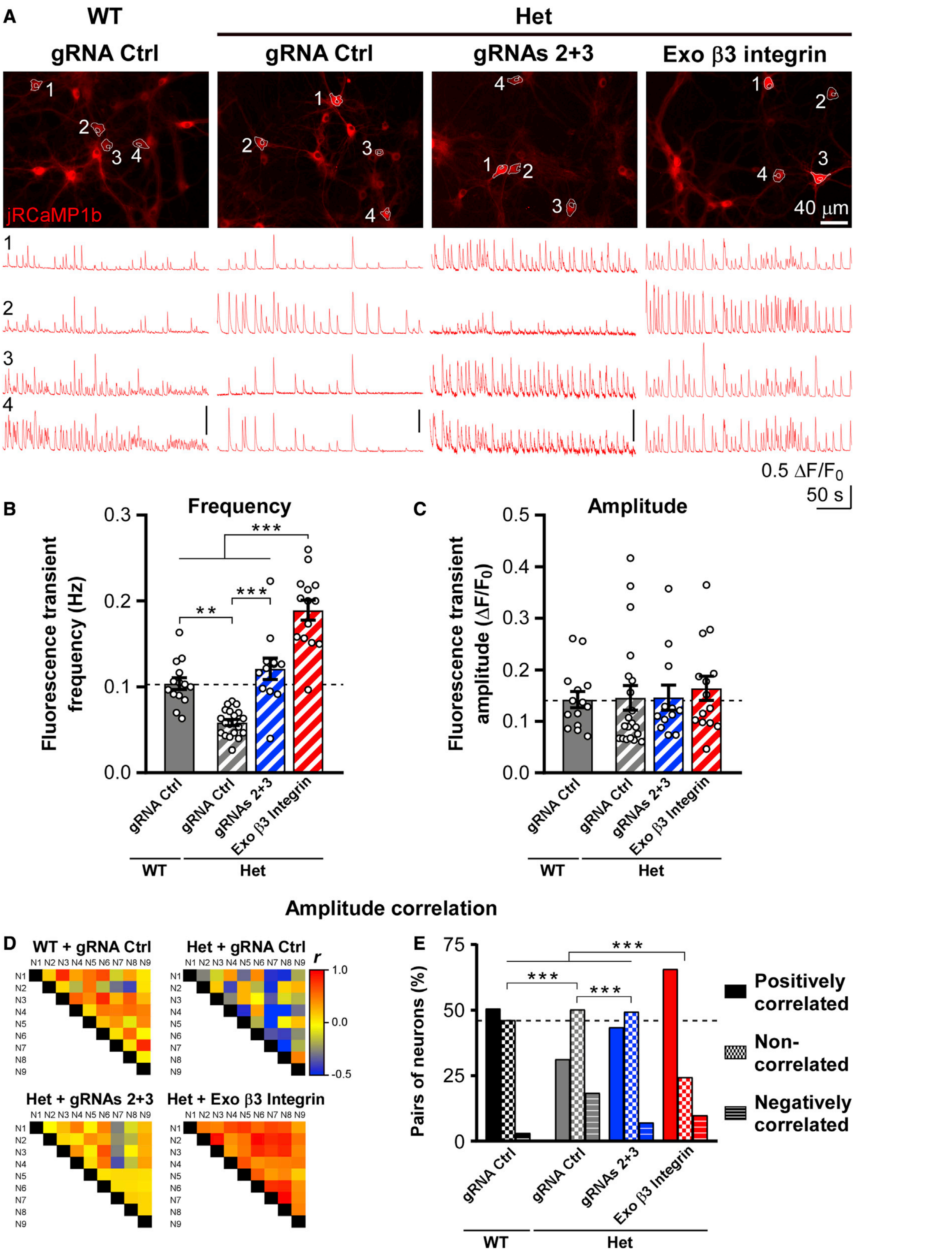

(legend on next page)

synapses (Figures S4E–S4I), interacts with mGluR5 (Figure 4K) and contributes at anchoring this group I mGluR at peri-synaptic locations (Figure 4). Group I mGluRs play a major role in the induction of both a Hebbian (mGluR-dependent long-term depression [mGluR-LTD]) and a homeostatic form of synaptic plasticity (synaptic down-scaling).[42,75] While in mGluR-LTD mGluR1/5 are activated by synaptically released glutamate, in homeostatic synaptic down-scaling, they disperse from the synapse to enter a constitutive, agonist-independent activated state.[42,76] The results from both the acute activation (Figures 3 and 4) and chronic blockade of mGluR1/5 (Figure 7) are in line with a model whereby *Itgb3* haplo-insufficiency favors extra-synaptic, constitutively active mGluR5 over synaptic, agonist-dependent mGluR5. This presumably reduces membrane expression of AMPARs independently of GluA1 phosphorylation (Figure 2, S2B, and S2C), excitatory synaptic strength,[30,45] and network excitability (Figure 1). The effects of β3 integrin are unlikely mediated by Homer1a because the transcript and protein for this gene were reduced and unchanged, respectively, in *Itgb3* Het neurons (Figures 2 and S2); elevated levels of Homer1a would instead be required to support dispersed and constitutively active mGluR5.[42,77] The function that we report here for β3 integrin at excitatory synapses appears similar to that played by other CAMs. For example, extracellular leucine-rich repeat and fibronectin type III domain-containing 1 (ELFN1) clusters and regulates the activity of mGluR7 at synapses on somatostatin interneurons.[78,79] It is therefore likely that CAMs and extracellular interactions cooperate with intracellular scaffolding proteins, such as short and long forms of Homer, in regulating localization and function of mGluRs.[41]

Dysregulation of mGluR5 signaling is a common feature of several neurological disorders, including schizophrenia, addictive disorders, Phelan-McDermid syndrome, and FXS, the most frequent monogenic form of ASD. While many of these disorders are characterized by a general down-regulation of mGluR5 and may benefit from mGluR5-positive modulators, FXS is associated with enhanced mGluR5 activity, and, indeed, various mGluR5 inhibitors can rescue cellular, electrophysiological, and behavioral defects of the $Fmr1^{-/y}$ mouse model of FXS.[34–37] The mGluR5 signaling defects that we identified in *Itgb3* Het neurons are closely reminiscent of those found in the $Fmr1^{-/y}$ mouse model. Like in *Itgb3* Het neurons, mGluR5 signaling in $Fmr1^{-/y}$ mice is not only potentiated but is also skewed toward an extra-synaptic activation mode.[77,80,81] Together with our results that *Fmr1* transcript levels are correlated to those of *Itgb3* (Figure S2) and the recent finding that fragile X mental retardation protein (FMRP; the product of *Fmr1*) binds the *Itgb3* mRNA in the juvenile hippocampus and cerebellum,[82] these data suggest therefore a potential convergence of the integrin and FMRP signaling pathways in ASD.

Finding new genetic approaches to correct metabotropic signaling in ASD is important because current pharmacological approaches based on mGluR antagonists, such as MPEP, albeit effective in preclinical studies, have failed to show significant benefits in humans.[34] These failures may be due to the intrinsic inability of pharmacology to discriminate between synaptic and extra-synaptic mGluR5 and could be overcome by genetic approaches, such as ours, aimed at anchoring mGluR5 to the synapse. More in general, patients with ASD exhibit an increase in copy-number variations.[1–4] Further, many ASD mutations affect a single allele.[5] These observations highlight the importance of gene dosage in ASD and the potential of CRISPRa-based strategies for rebalancing gene deficiency in ASD.

Here, we designed CRISPRa tools to activate *Itgb3*, which is deficient in some cases of ASD,[7,11,12,14,83] and demonstrated that *Itgb3* haplo-insufficiency can be compensated for both *in vitro* and *in vivo* by activating the transcription of the remaining functional allele. By contrast, overexpression led to a ∼50-fold increase in β3 integrin protein levels, aberrant mGluR5 expression, and hyperactive cortical networks (Figures 5, 6, S4, and S10). Thus, whenever possible, CRISPRa should be preferred to overexpression of exogenous genes in rescue experiments and future therapeutic strategies. Although CRISPRa holds great promise for diseases caused by haplo-insufficiency, it is nevertheless not suitable for compensating dominant mutations as it would increase expression of both functional and aberrant alleles.

MATERIALS AND METHODS

Experimental model and subject details

All experiments were performed in accordance with EU and Italian legislation (authorization no. 1168/2020-PR). *Itgb3* KO and Het mice (B6;129S2-Itgb3tm1Hyn/J, Jackson Laboratory) were described previously[30,31] and were backcrossed to the C57Bl/6j background >10 times at the time of experiments. For *in vivo* experiments, male CaMKIIa-CreTg/Tg mice[31] were crossed with female $Itgb3^{+/-}$ mice to obtain CaMKIIa-CreTg/+;$Itgb3^{+/+}$ and CaMKIIa-CreTg/+;$Itgb3^{+/-}$ littermates for rAAV injections.

gRNA design and plasmid construction

We used the web tool http://crispr.mit.edu/, which maximizes the regions with low off-target probability, to design six gRNAs targeting the region from −100 to −200 bp relative to the TSS of the *Itgb3* gene. As negative Ctrl (gRNA Ctrl), we used a non-targeting gRNA

Figure 6. CRISPRa normalizes spontaneous activity in *Itgb3* Het neurons

(A) Top, representative images of jRCaMP1b in WT and *Itgb3* Het primary cortical neurons expressing the indicated constructs. Images are an average over a 5 min recording period. Bottom, spontaneous somatic fluorescence responses from the ROIs shown in the above panels. (B and C) Quantification of experiments as in (A). CRISPRa rescues the reduction in fluorescence transient frequency of Het neurons (**p < 0.01, ***p < 0.001, one-way ANOVA followed by Tukey's post-test, n = 12–20 fields of view from 5 independent cultures). Data are shown as mean ± SEM; dots represent individual values. (D) Heatmaps of Pearson's correlation coefficients (*r*) for fluorescence transient amplitudes from representative fields of view. *r* correlates with β3 integrin levels. (E) Quantification of experiment as in (D). The graph shows the percentage of positive *r* with a p <0.05 (positively correlated), negative *r* with a p <0.05 (negatively correlated), and *r* with a p >0.05 (non-correlated; ***p < 0.001, chi-square test; n = 158–396 pairs). See also Figure S8.

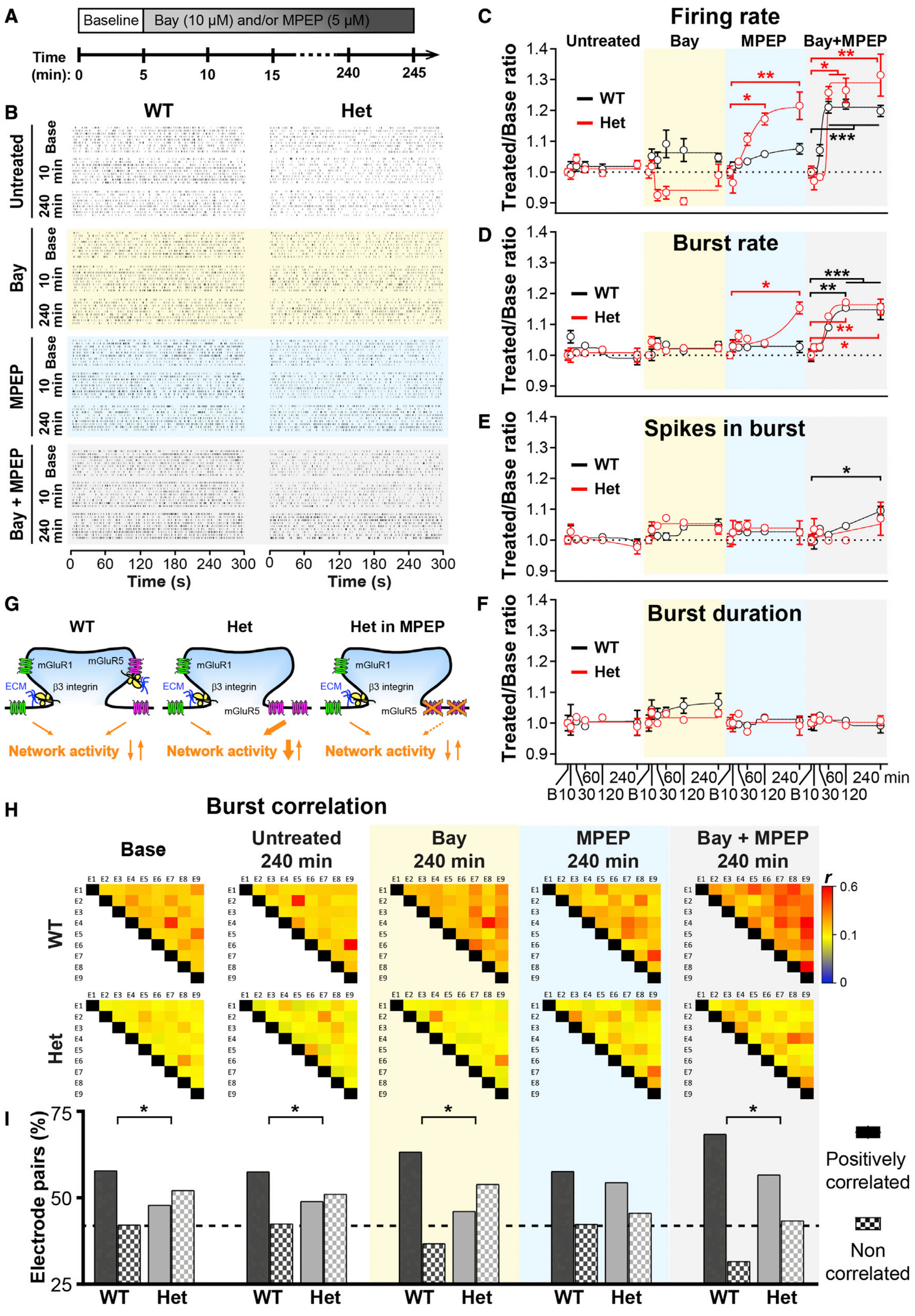

(legend on next page)

sequence (Figure S3A). The pU6-(BbsI)-EF1a-dCas9-VP64-T2A-EGFP plasmid (Figure S3A), used to co-express a gRNA, the dCas9-VP64 fusion protein, and EGFP, was constructed by inserting the EF1a-dCas9-VP64-T2A-EGFP cassette from the dCAS9-VP64-GFP plasmid (gift from Feng Zhang; cat. no. 61422, Addgene)[58] in place of the CBh-Cas9-T2A-mCherry cassette of the pU6-(BbsI)-CBh-Cas9-T2A-mCherry plasmid (gift from Ralf Kuehn; cat. no. 64324, Addgene). The gRNA sequences were inserted downstream of the U6 promoter using the BbsI cloning sites. The lentiviral vectors pLL-U6-(gRNA)-EF1a-dCas9-VP64-T2A-EGFP (Figure S4A) were constructed by inserting the cassette U6-gRNA Ctrl, U6-gRNA 2, or U6-gRNA 3 from the pU6-(gRNA)-EF1a-dCas9-VP64-T2A-EGFP plasmids described above in dCAS9-VP64-GFP (cat. no. 61422, Addgene) using the PacI and AgeI sites. Human β3 integrin was expressed under the control of the short human Synapsin promoter using the lentiviral vector pLL-Syn-EGFP-P2A-ITGB3.[31] The rAAV vector pAAV-Syn-DIO-Sa-dCas9-VPR (Figure 8A) was constructed from pJEP313-pAAV-CMV-Sa-Cas9-DIO-pA (gift from Jonathan Ploski; cat. no. 113690, Addgene) by replacing the CMV promoter with the human Synapsin promoter from pAAV-hSyn-EGFP (gift from Bryan Roth; cat. no. 50465, Addgene) and Sa-Cas9 with Sa-dCas9-VPRmini from pAAV-CMV-dSa-VPRmini-syn-pA (gift from George Church; cat. no. 99689, Addgene).[84] For the rAAV vectors pAAV-U6-gRNA-Syn-DIO-EGFP (Figure 8A), we first inserted the gRNA sequences downstream of the U6 promoter in BPK2660 (gift from Keith Joung; cat. no. 70709, Addgene)[85] using the BsmbI cloning sites. We then inserted the cassette U6-gRNA Ctrl, U6-gRNA 4, or gRNA 5 from the BPK2660-gRNA plasmids into pAAV-hSyn-EGFP-DIO (gift from Bryan Roth; cat. no. 50457, Addgene) using the MluI cloning site. Constructs were generated by standard cloning strategies and verified by sequencing.

N2a cell culture and transfection

N2a mouse neuroblastoma cells were cultured in Dulbecco's modified Eagle medium (DMEM, Gibco) supplemented with 10% FBS, 2 mM glutamine, 100 U/mL penicillin, and 0.1 mg/mL streptomycin (complete culture medium) and were maintained in a 5% CO_2 humidified incubator at 37°C.[86] Transfection was performed in 60%–70% confluent cultures seeded in 6-well plates at 200,000 cells/well in complete culture medium the previous day. Cells were transfected with 3 μg DNA/well using the Ca^{2+} phosphate method[87] and used 24–48 h post-transfection.

Cell adhesion assay

N2a cells were trypsinized 2 days after transfection and seeded in complete culture medium on fibronectin-coated coverslips (5 μg/mL for 16 h; cat. no. F8141, Sigma) in 24-well plates at a density of 100,000 cells/well. After 1 h at 37°C, coverslips were washed 4 times with PBS to remove non-attached cells; remaining adherent cells were fixed in 4% PFA, stained with Hoechst, and mounted with ProLong Gold mounting medium (Thermo Fisher Scientific). To quantify the number of attached cells, three images per coverslip were taken using a Leica SP8 confocal microscope with a 40× oil immersion objective (numerical aperture [NA] 1.30); for each condition, six coverslips from three independent cultures were imaged in total.

Primary cortical culture

Cortical neuronal cultures were prepared from P0 *Itgb3*$^{+/+}$ (WT), *Itgb3*$^{+/-}$ (Het), or *Itgb3*$^{-/-}$ (KO) pups as previously described,[88,89] with minor modifications. Briefly, cortices were dissected in ice-cold HBSS, digested with papain (30 U; cat. no. 3126, Worthington) for 40 min at 37°C, washed, and triturated in attachment medium (BME medium supplemented with 10% FBS, 3 mg/mL glucose, 1 mM sodium pyruvate, and 10 mM HEPES-NaOH [pH 7.40]) with a flame-polished glass Pasteur pipette. For qRT-PCR and western blot experiments, cells were seeded at a concentration of 750,000 cells/well onto 6-well plates coated with 2.5 μg/mL poly-D-lysine (PDL; P7405, Sigma) and 1 μg/mL laminin (L2020, Sigma); for confocal microscopy and Ca^{2+} imaging experiments, cells were seeded at 75,000/well onto 1.2 cm diameter glass coverslips coated with PDL/laminin as above. After 4 h, the attachment medium was replaced with maintenance medium (neurobasal medium supplemented with 2.6% B27, 6 mg/mL glucose, 2 mM GlutaMax, 90 U/mL penicillin, and 0.09 mg/mL streptomycin). To prevent glia overgrowth, 0.5 μM of cytosine β-D-arabinofuranoside (AraC) was added at 4–5 days *in vitro*.

Lentivirus production and infection

HEK293T cells were maintained in Iscove's modified Dulbecco's medium supplemented with 10% FBS, 2 mM glutamine, 100 U/mL penicillin, and 0.1 mg/mL streptomycin in a 5% CO_2 humidified incubator at 37°C. Cells were transfected with the Δ8.9 encapsidation plasmid, the VSVG envelope plasmid, and the pLL-U6-(gRNA)-EF1a-dCas9-VP64-T2A-EGFP or the pLL-Syn-EGFP-P2A-ITGB3 plasmid described above using the Ca^{2+} phosphate method. The transfection medium was replaced by fresh medium after 14 h. Supernatants were collected 36 to 48 h after transfection, centrifuged to remove cell debris, passed through a 0.45 μm filter, and ultra-centrifuged for

Figure 7. Chronic blockade of mGluR5 rescues impaired network activity of *Itgb3* Het neurons

(A) Experimental timeline of MEA experiments with mGluR1 (Bay; 10 μM) and mGluR5 (MPEP; 5 μM) inverse agonists. (B) Representative raster plots of network activity from WT and *Itgb3* Het cultures for the indicated conditions. (C–F) Quantification of experiments as in (A) and (B) after 10, 30, 60, 120, and 240 min long applications of the indicated drugs. Values are normalized to baseline (B) for each recording. Chronic blockade of mGluR5 with MPEP increases firing and burst rate selectively in *Itgb3* Het cultures (*p < 0.05, **p < 0.01, ***p < 0.001, two-way ANOVA followed by Dunnett post-test; n = 4–8 per condition; 3 independent cultures). Data are presented as mean ± SEM. Lines through points are sigmoid fits. (G) Scheme depicting the action of the mGluR5 inverse agonist MPEP on *Itgb3* Het neurons. (H) Representative heatmaps of Pearson's correlation coefficients (*r*) for burst activity from wells containing 9 electrodes (E1–E9). (I) Quantification of experiment as in (H). All electrode pairs exhibited a positive *r*. The graph shows the percentage of *r* with a p <0.05 (positively correlated) and a p >0.05 (non-correlated; *p < 0.05, chi-square test; n = 128–280 pairs). Chronic blockade of mGluR5 with MPEP rescues burst synchrony in *Itgb3* Het cultures.

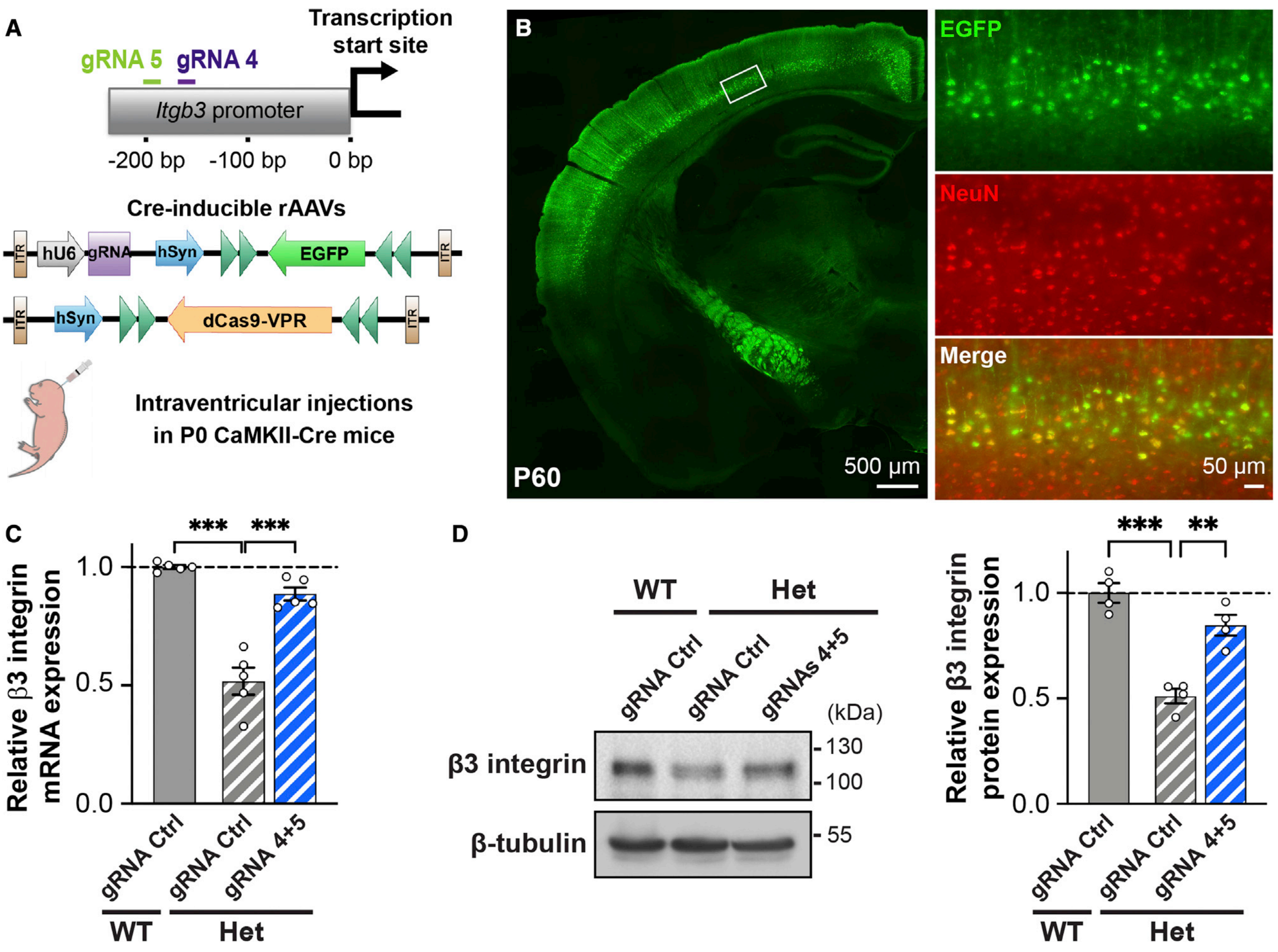

Figure 8. Normalization of β3 integrin expression *in vivo* by CRISPRa

(A) gRNA targets on the *Itgb3* promoter and rAAV constructs for *in vivo* experiments. (B) Coronal section of injected P60 mouse showing EGFP expression in deep layer cortical pyramidal neurons. (C) qRT-PCR quantification of *Itgb3* mRNA levels in cortex of WT and *Itgb3* Het P60 mice expressing the indicated constructs (n = 5 cortices per group). (D) Left, western blots of membrane fractions from cortices of P60 mice. Right, western blot quantification (n = 4 cortices per group). CRISPRa rescues *Itgb3* gene dosage in *Itgb3* Het neurons *in vivo* at both the mRNA and protein level (**p < 0.01, ***p < 0.001, one-way ANOVA followed by Tukey's post-test). Data are presented as mean ± SEM; dots represent individual values. See also Figure S9.

2 h at 20,000 g at 4°C. Viral pellets were re-suspended in PBS, aliquoted, and stored at −80°C until use.[87] Neuronal cultures were infected at 6 DIV with the lowest infectious dose capable of transducing ≥95% of neurons (dilution range: 1:300 to 1:700) and used for experiments after ≥10 days (Figure S4A).

Western blotting

Membrane protein-enriched fractions were prepared from cortical neurons at 16 DIV as previously described.[87] Briefly, cells were washed once in ice-cold PBS and scraped in 100 µL buffer A (25 mM Tris-HCl [pH 7.4], 150 mM NaCl, 2 mM KCl, 2.5 mM EDTA) supplemented with protease and phosphatase inhibitors (complete EDTA-free protease inhibitors [cat. no. 1187358001, Roche]; serine/threonine and tyrosine phosphatase inhibitors [cat. nos. P0044 and P5726, Sigma]). After removal of the cell debris at 1,000 × *g*, 4°C, for 10 min, the supernatant was centrifuged at 15,000 × *g*, 4°C, for 15 min. The resulting pellet was dissolved in 100 µL RIPA buffer (50 mM Tris [pH 8.0], 150 mM NaCl, 1% NP-40, 0.5% sodium deoxycholate, 0.1% SDS) and centrifuged at 15,000 × *g*, 4°C, for 15 min. The resulting supernatant was used for western blot analysis. Protein concentration was quantified with the BCA Protein Assay kit (cat. no. 23227, Thermo Fisher Scientific). Proteins were separated by SDS-PAGE using 7.5% acrylamide gels and transferred on polyvinylidene fluoride (PVDF) membranes. After incubation with primary rabbit anti-β3 integrin (1:200; cat. no. 4702, Cell Signaling); rabbit anti-mGluR1 (1:1,000; cat. no. 191 002, Synaptic Systems); rabbit anti-mGluR5 (1:200; cat. no. AB5675, Millipore), mouse anti-GluA2 (1:500; cat. no. MAB397, Millipore); rabbit anti-GluA2/3 (1:200; cat. no. 07-598, Millipore); rabbit anti-GluA1 (1:500; cat. no. AB1504, Millipore);

rabbit anti-GluA1 phosphoSer831 (1:500; cat. no. AB5847, Millipore); rabbit anti-GluA1 phosphoSer845 (1:500; cat. no. AB5849, Millipore); rabbit anti-Homer1a (1:1,000; cat. no. 160 013, Synaptic Systems); or rabbit anti-β-tubulin III (1:1,000; cat. no. T2200, Sigma) antibodies, membranes were incubated with secondary HRP-conjugated goat anti-rabbit antibody (1: 5,000; cat. no. 31460, Thermo Fisher Scientific) or goat anti-mouse antibody (1: 5,000; cat. no. 31430, Thermo Fisher Scientific), and immunocomplexes were detected with the chemiluminescent substrate (cat. no. RPN2106, ECL Prime Western Blotting System, GE Healthcare). We acquired chemiluminescent signals using a ChemiDoc imaging system (Biorad) and quantified immunoreactive bands using ImageJ (http://rsb.info.nih.gov/ij). Band intensity from different samples was normalized to that of WT Ctrl within the same membrane. Intensity of phosphorylated proteins was normalized to the total amount of the corresponding proteins.

Immunoprecipitation

Adult male C57BL/6j mice (3–5 months) were deeply anesthetized and transcardially perfused with ice-cold phosphate buffer to remove β3-integrin-rich platelets from brain tissue. Cortices were immediately dissected on ice, flash frozen in liquid N_2, and stored at $-80^{\circ}C$ until use. On the day of the experiment, cortices were defrosted on ice, homogenized with 15 strokes in buffer A (in mM: sucrose, 320; HEPES, 10; $CaCl_2$, 0.2; $MgCl_2$, 1; supplemented with protease inhibitors [complete EDTA-free protease inhibitors]) using a Douncer at 300 RPM. The homogenate was centrifuged at 1,000 × *g*, 4°C, for 10 min, and the supernatant collected and further centrifuged at 50,000 × *g*, 4°C, for 15 min. The pellet was then re-suspended in 0.5 mL buffer B (in mM: NaCl, 100; HEPES, 20; $CaCl_2$, 0.2; $MgCl_2$, 1; containing 1% Triton X-100, supplemented with complete EDTA-free protease inhibitors and serine/threonine and tyrosine phosphatase inhibitors) per mouse brain and incubated on ice for 15 min, before clearing the lysate from undissolved particles by centrifugation at 100,000 × *g*, 4°C, for 30 min. The so-obtained lysate was incubated overnight with Sepharose-ProteinG (Dynabeads, Thermo Fisher Scientific) coupled with hamster anti-CD61 (cat. no. 553343, BD Pharmingen) or rabbit anti-mGluR5 (cat. no. AB5675, Merck) in a total volume of 400 μL per sample. As negative controls served non-immune hamster IgG (cat. no. PA5-33219, Pierce) or rabbit anti-RFP (cat. no. 600-401-379, Rockland), respectively. The depleted lysate (flow trough) was removed, and the beads were washed 3 times in buffer B before elution of the immunoprecipitate with gel loading dye for 10 min at 70°C. Fractions were analyzed by western blot.

RNA extraction and qRT-PCR

Total RNA was extracted with QIAzol lysis reagent (cat. no. 79306, Qiagen) from primary cortical cultures at 16 DIV or cortex of P60 mice, as previously described.[87] We prepared cDNAs by reverse transcription of 1 μg of RNA using the QuantiTect Reverse Transcription Kit (cat. no. 205311, Qiagen). qRT-PCR was performed in triplicate with 10 ng of template cDNA using iQ SYBR Green Supermix (cat. no. 1708886, Biorad) on a CFX96 Real-Time PCR Detection System (Biorad) with the following universal conditions: 5 min at 95°C, 45 cycles of denaturation at 95°C for 15 s, and annealing/extension at 60°C for 45 s. Primers were designed with Primer-BLAST (www.ncbi.nlm.nih.gov/tools/primer-blast) to avoid significant crosshomology regions with other genes. Product specificity and absence of primer dimers was verified by melting curve analysis and agarose gel electrophoresis. qPCR reaction efficiency for each primer pair was calculated by the standard curve method with a five points serial dilution of cDNA. Calculated qPCR efficiency for each primer set was used for subsequent analysis. The relative quantification of gene expression was determined using the ΔΔCt method. Data were normalized to glyceraldehyde-3-phosphate dehydrogenase (GAPDH), β-actin (ACTB), and hypoxanthine phosphoribosyltransferase 1 (HPRT1) by the multiple internal Ctrl gene method with GeNorm algorithm. mRNA expression was normalized to WT Ctrl samples within the same qRT-PCR plate. Sequences of all the primers used are listed in Table S1.

ChIP in primary neurons

After crosslinking with 1% formaldehyde for 10 min and quenching with 125 mM glycine for 5 min, we extracted the chromatin from transduced primary cortical neurons using the Chromatin Extraction kit (cat. no. ab117152, Abcam) according to the manufacturer's instructions. After sonication, samples were further processed for ChIP using the ChIP Kit-One step (cat. no. ab117138, Abcam) and a rabbit polyclonal anti-Cas9 antibody (cat. no. C15310258, Diagenode) or a Ctrl non-immune immunoglobulin G (IgG) (cat. no. ab117138, Abcam; Figure S5B). Enrichment of target regions was assessed by qRT-PCR as detailed in previous section using the primers listed in Table S1.

In vivo genome-wide ChIP-seq

Chromatin was extracted from cortices of P60 mice (two biological replicates per condition) injected with pAAV-Syn-DIO-Sa-dCas9-VPR and pAAV-U6-gRNA-Ctrl-Syn-DIO-EGFP (gRNA Ctrl condition; 1:2 rAAV ratio) or pAAV-Syn-DIO-Sa-dCas9-VPR, pAAV-U6-gRNA-4-Syn-DIO-EGFP, and pAAV-U6-gRNA-5-Syn-DIO-EGFP (gRNA 4+5 condition; 1:1:1 rAAV ratio) and prepared using the SimpleChIP Plus Enzymatic Chromatin IP Kit (cat. no. 9005, Cell Signaling) according to the manufacturer's instructions. After chromatin shearing, immunoprecipitation was performed using an anti *S. aureus* Cas9 antibody (cat. no. C15200230, Diagenode). Library preparation and sequencing on the Illumina HiSeq/NovaSeq platform were performed by Novogene (Cambridge, UK). Sequencing reads were mapped to the genome using BWA.[90] Mapping was restricted to reads that were uniquely assigned to the mouse genome (GRCm38.p6). Biological replicates were pooled to call peaks of gRNAs 4 + 5 versus gRNA Ctrl using MACS2.[91]

Confocal microscopy and image analysis

Ten days post-infection, cultures were fixed for 8 min with 4% PFA/4% sucrose at room temperature (RT), treated for 10 min at 50°C with a sodium citrate solution (10 mM tri-sodium citrate dihydrate [pH 6.0], 0.05% Tween-20) to retrieve the β3 integrin antigen,[92] and permeabilized for 10 min at RT with 0.1% TritonX-100. β3 integrin staining was revealed using a rabbit monoclonal anti-integrin β3 (1:200; cat. no. 13166, Cell Signaling) and the Tyramide SuperBoost

kit (cat. no. B40922, Thermo Fisher Scientific) with the Alexa Fluor 568 Tyramide Reagent (10 min, 1:10 dilution; cat. no. B40956, Thermo Fisher Scientific) before counterstaining for GFP and vGlut1 using chicken anti-GFP (1:1,000; cat. no. AB13970, Abcam) and guinea pig anti-vGlut1 (1:500; cat. no. 135304, Synaptic System), respectively. For mGluR5, we used rabbit anti-mGluR5 (1:200; cat. no. AB5675, Millipore) at 16 DIV without antigen retrieval treatment. Secondary antibodies were Alexa Fluor 488-conjugated anti-chicken (1:1,000; cat. no. A11039, Thermo Fisher Scientific); Dylight405-conjugated donkey anti-guinea pig (1:150; cat. no. 706-475-148, Jackson ImmunoResearch); Alexa Fluor 488-conjugated anti-rabbit (1:1,000; cat. no. A11034, Thermo Fisher Scientific); and Alexa Fluor 647-conjugated anti-guinea pig (1:1,000; cat. no. A21450, Thermo Fisher Scientific). Confocal stacks were acquired at 200 Hz with a Leica SP8 using a 63× oil immersion objective (NA 1.40), 1.2× digital zoom, 0.15 μm pixel size, 1 AU pinhole, 0.3 μm between optical sections, with a sequential line-scan mode and 3× scan averaging. For all experimental conditions compared, we used the same settings for laser intensity, offset, and PMT gain. Confocal images were analyzed using ImageJ. Each stack was filtered using a Gaussian filter (radius: 0.5 pixels), and the maximal fluorescence intensities of in-focus stacks were Z-projected. For Figure S4, the images were automatically thresholded using the Robust Automatic Threshold Selection plugin followed by the watershed algorithm. Dendritic analysis was performed on dendritic regions of interest (ROIs) of 40–120 μm in length, manually selected in the GFP channel blind to the experimental condition. Co-localization was estimated for the thresholded ROIs with the Coloc2 plugin using the Manders' coefficients ($M_A = \sum_i A_{i,coloc}/\sum_i A_i$, where $\sum_i A_i$ is the sum of intensities of all pixels above threshold for channel A and $\sum_i A_{i,coloc}$ is calculated as $\sum_i A_i$ but only for pixels where also the second channel B is above threshold). For Figure 4, the images were automatically thresholded using the Auto Local Threshold Otsu with a radius of 15 pixels. Blind to the genotype, we used the Time Series Analyzer v.3 plugin to position circular ROIs (Ø = 2.17 μm) on vGlut1-positive dendritic protrusions (operationally defined as dendritic spines). These were used to obtain the synaptic signal (S) in the mGluR5 channel. For the dendritic mGluR5 signal (D), we positioned a second ROI adjacent to the first on the parental dendritic shaft. The intensity of a third ROI positioned within 10 μm from the first two was used to subtract the local background noise. Background subtracted S and D signals were quantified as relative difference ((S−D)/(S+D)) for each spine/dendritic shaft pair. P60 mice were anesthetized and intracardially perfused with 4% PFA. The brain was postfixed, cryoprotected in 30% sucrose, and embedded in optical cutting temperature (OCT) compound; frozen sagittal sections (40 μm) were cut with a cryostat. Sections were permeabilized with 0.3% Triton X-100 for 10 min, blocked for 30 min with 5% normal goat serum (NGS), and then incubated with primary antibodies (chicken anti-GFP and mouse anti-NeuN; 1:500; cat. no. MAB377, Merck-Millipore) for 2 h and with secondary antibodies (Alexa Fluor 488-conjugated anti-chicken and Alexa Fluor 568-conjugated anti-mouse; 1:500; cat. no. A11004, Thermo Fisher Scientific) for 1 h. Sections were imaged using a Nikon Eclipse E800 epifluorescence microscope with a 20× objective.

MEA recordings

Cortical neurons were seeded at 150,000 cells/well on 6-well MEAs (Multichannel Systems) coated with PDL/laminin. Each well contained nine electrodes (30 μm diameter; 200 μm center-to-center spacing). Neurons were transduced at 6 DIV, and network activity was recorded at 16–17 DIV using a MEA1060INV amplifier (Multichannel Systems). Neurons were kept in maintenance medium at 37°C throughout the recordings. To ensure stabilization of the electrical signal, experiments were initiated 10 min after transferring the MEAs from the incubator to the setup. Network activity was recorded for 5 min under basal conditions and for a further 5 min at the indicated time points upon drug application.

Spike detection and spike train analysis were performed with the MC-Rack software (Multichannel Systems). Spike threshold was set for each electrode at 5 times the standard deviation of the baseline noise level. Bursts were operationally defined as a collection of a minimum number of spikes (N_{min} = 5) separated by a maximum interspike interval (ISI_{max} = 100 ms). Following spike and burst detection, we computed mean firing rate, burst rate, mean burst duration, percentage of spike in burst, and intraburst spike frequency. To evaluate network synchrony, we computed the Pearson's correlation coefficient (r) of burst activity for all electrode pairs from each MEA well. Electrodes were not included in the analysis if they recorded less than 5 bursts over 5 min recording period.

Ca^{2+} imaging

Imaging was performed in primary cortical cultures at 30°C ± 2°C in aCSF containing (in mM): 140 NaCl, 3.5 KCl, 2.2 $CaCl_2$, 1.5 $MgCl_2$, 10 D-glucose, and 10 HEPES-NaOH (pH 7.38; osmolarity adjusted to 290 mOsm). In initial experiments, we tested the red-shifted genetically encoded Ca^{2+} indicators (GECIs) jRGECO1a, jRCaMP1a, and jRCaMP1b[38] and chose jRCaMP1b for subsequent experiments as it provided overall the best signal-to-noise ratio, the largest dynamic range, and the best temporal resolution of the three GECIs in our experimental conditions. Cultures were infected with the appropriate lentivirus and pAAV.Syn.NES-jRCaMP1b.WPRE.SV40 (cat. no. 100851-AAV1, Addgene; titer: $1.7*10^{13}$ GC/mL; dilution 1: 50,000) 9–12 and 4–6 days prior to experiments, respectively, and recorded at 15–18 DIV. Imaging was performed with a cooled charge-coupled device (CCD) camera (ORCA-R2, Hamamatsu) mounted on an inverted microscope (DMI6000B, Leica) with a 20×, 0.75 NA glycerol immersion objective. A 200 W metal halide lamp (Lumen200Pro, Prior Scientific) and a filter set comprising a BP 515–560 nm excitation filter, a 580 nm dichroic mirror, and an LP 590 emission filter (filter set N2.1, Leica) were used for illumination. Images were captured at 15.3 Hz with 50 ms integration times at a depth of 8 bits. Network activity was recorded for 5 min. Images were analyzed in ImageJ with the plugin Time Series Analyzer v.3.0. ROIs were manually drawn on the soma (excluding the nuclear region; Figure 6A) of each neuron exhibiting at least one spontaneous fluorescence transient above two SD of the background noise during 5 min recording period. The intensity of twin ROIs positioned within 50 μm were

used to subtract the background noise. Signals were quantified as $\Delta F/F_0$, where $\Delta F = F - F_0$, with F_0 measured over 1 s period preceding the fluorescence transient. The Pearson's correlation coefficient for fluorescence transient amplitude was computed for all neuron pairs in each field of view.

rAAV production and injection

rAAVs (AAV-PHP.eB serotype) were prepared by the AAV Vector Unit at the International Centre for Genetic Engineering and Biotechnology Trieste, as previously described.[93] Injections were performed into the lateral ventricles (two-fifths of the distance from the lambda suture to each eye) at P0 (1.5 μL per ventricle), as previously described.[94]

Statistical analysis

Statistical differences were assessed using unpaired two-tailed Student's t test and one-way analysis of variance (ANOVA) test followed by Tukey-Kramer post-test and two-way ANOVA, as required. The chi-square test was used for Figures 3I, 5I, 6E, 7I, S6F, and S8C (Prism 7, GraphPad Software). Average data are expressed as mean ± SEM.

Data availability statement

All data generated or analyzed during this study are included in this published article and its supplemental information files.

SUPPLEMENTAL INFORMATION

Supplemental information can be found online at https://doi.org/10.1016/j.omtn.2022.07.006.

ACKNOWLEDGMENTS

We thank F. Benfenati (IIT) for support, D. Moruzzo and E. Colombo (IIT) for technical help, and F. Benfenati (IIT), M. Letellier (IIN), P. Baldelli (UniGe), and members of the Cingolani lab for critical reading of the manuscript. This work was supported by the Compagnia San Paolo (proposal ID: 2015 0702 to L.A.C.), the Cariplo Foundation (proposal ID: 2019-3438 to L.A.C.), and Italian Insitute of Technology/Istituto Italiano di Tecnologia (IIT). Part of the data appeared in preprint at https://www.biorxiv.org/content/10.1101/664706v1.

AUTHOR CONTRIBUTIONS

F.J., A.T., and L.A.C. designed and performed experiments, analyzed data, and wrote the manuscript. L.Z. produced rAAVs.

DECLARATION OF INTERESTS

The authors declare no competing interests.

REFERENCES

1. Glessner, J.T., Wang, K., Cai, G., Korvatska, O., Kim, C.E., Wood, S., Zhang, H., Estes, A., Brune, C.W., Bradfield, J.P., et al. (2009). Autism genome-wide copy number variation reveals ubiquitin and neuronal genes. Nature *459*, 569–573.

2. Levy, D., Ronemus, M., Yamrom, B., Lee, Y.H., Leotta, A., Kendall, J., Marks, S., Lakshmi, B., Pai, D., Ye, K., et al. (2011). Rare de novo and transmitted copy-number variation in autistic spectrum disorders. Neuron *70*, 886–897.

3. Pinto, D., Pagnamenta, A.T., Klei, L., Anney, R., Merico, D., Regan, R., Conroy, J., Magalhaes, T.R., Correia, C., Abrahams, B.S., et al. (2010). Functional impact of global rare copy number variation in autism spectrum disorders. Nature *466*, 368–372.

4. Sebat, J., Lakshmi, B., Malhotra, D., Troge, J., Lese-Martin, C., Walsh, T., Yamrom, B., Yoon, S., Krasnitz, A., Kendall, J., et al. (2007). Strong association of de novo copy number mutations with autism. Science *316*, 445–449.

5. de la Torre-Ubieta, L., Won, H., Stein, J.L., and Geschwind, D.H. (2016). Advancing the understanding of autism disease mechanisms through genetics. Nat. Med. *22*, 345–361.

6. Sherman, M.A., Rodin, R.E., Genovese, G., Dias, C., Barton, A.R., Mukamel, R.E., Berger, B., Park, P.J., Walsh, C.A., and Loh, P.R. (2021). Large mosaic copy number variations confer autism risk. Nat. Neurosci. *24*, 197–203.

7. O'Roak, B.J., Vives, L., Girirajan, S., Karakoc, E., Krumm, N., Coe, B.P., Levy, R., Ko, A., Lee, C., Smith, J.D., et al. (2012). Sporadic autism exomes reveal a highly interconnected protein network of de novo mutations. Nature *485*, 246–250.

8. Ruzzo, E.K., Pérez-Cano, L., Jung, J.Y., Wang, L.K., Kashef-Haghighi, D., Hartl, C., Singh, C., Xu, J., Hoekstra, J.N., Leventhal, O., et al. (2019). Inherited and de novo genetic risk for autism impacts shared networks. Cell *178*, 850–866.e26.

9. Satterstrom, F.K., Kosmicki, J.A., Wang, J., Breen, M.S., De Rubeis, S., An, J.Y., Peng, M., Collins, R., Grove, J., Klei, L., et al. (2020). Large-Scale exome sequencing study implicates both developmental and functional changes in the neurobiology of autism. Cell *180*, 568–584.e23.

10. Alonso-Gonzalez, A., Calaza, M., Amigo, J., González-Peñas, J., Martínez-Regueiro, R., Fernández-Prieto, M., Parellada, M., Arango, C., Rodriguez-Fontenla, C., and Carracedo, A. (2021). Exploring the biological role of postzygotic and germinal de novo mutations in ASD. Sci. Rep. *11*, 319.

11. Cantor, R.M., Kono, N., Duvall, J.A., Alvarez-Retuerto, A., Stone, J.L., Alarcón, M., Nelson, S.F., and Geschwind, D.H. (2005). Replication of autism linkage: fine-mapping peak at 17q21. Am. J. Hum. Genet. *76*, 1050–1056.

12. Weiss, L.A., Kosova, G., Delahanty, R.J., Jiang, L., Cook, E.H., Ober, C., and Sutcliffe, J.S. (2006). Variation in ITGB3 is associated with whole-blood serotonin level and autism susceptibility. Eur. J. Hum. Genet. *14*, 923–931.

13. Jaudon, F., Thalhammer, A., and Cingolani, L.A. (2021). Integrin adhesion in brain assembly: from molecular structure to neuropsychiatric disorders. Eur. J. Neurosci. *53*, 3831–3850.

14. Carter, M.D., Shah, C.R., Muller, C.L., Crawley, J.N., Carneiro, A.M.D., and Veenstra-Vanderweele, J. (2011). Absence of preference for social novelty and increased grooming in integrin beta3 knockout mice: initial studies and future directions. Autism Res. *4*, 57–67.

15. American-Psychiatric-Association (2013). Diagnostic and Statistical Manual of Mental Disorders, 5th ed. (Arlington, VA: American Psychiatric Publishing).

16. Kerrisk, M.E., Cingolani, L.A., and Koleske, A.J. (2014). ECM receptors in neuronal structure, synaptic plasticity, and behavior. Prog. Brain. Res. *214*, 101–131.

17. Bachmann, M., Kukkurainen, S., Hytönen, V.P., and Wehrle-Haller, B. (2019). Cell adhesion by integrins. Physiol. Rev. *99*, 1655–1699.

18. Kechagia, J.Z., Ivaska, J., and Roca-Cusachs, P. (2019). Integrins as biomechanical sensors of the microenvironment. Nat. Rev. Mol. Cell. Biol. *20*, 457–473.

19. Cully, M. (2020). Integrin-targeted therapies branch out. Nat. Rev. Drug. Discov. *19*, 739–741.

20. Ferrer-Ferrer, M., and Dityatev, A. (2018). Shaping synapses by the neural extracellular matrix. Front. Neuroanat. *12*, 40.

21. Lilja, J., and Ivaska, J. (2018). Integrin activity in neuronal connectivity. J. Cell. Sci. *131*, jcs212803.

22. Park, Y.K., and Goda, Y. (2016). Integrins in synapse regulation. Nat. Rev. Neurosci. *17*, 745–756.

23. Thalhammer, A., and Cingolani, L.A. (2014). Cell adhesion and homeostatic synaptic plasticity. Neuropharmacology *78*, 23–30.

24. Babayan, A.H., Kramár, E.A., Barrett, R.M., Jafari, M., Häettig, J., Chen, L.Y., Rex, C.S., Lauterborn, J.C., Wood, M.A., Gall, C.M., and Lynch, G. (2012). Integrin dynamics produce a delayed stage of long-term potentiation and memory consolidation. J. Neurosci. *32*, 12854–12861.

25. Chan, C.S., Weeber, E.J., Zong, L., Fuchs, E., Sweatt, J.D., and Davis, R.L. (2006). Beta 1-integrins are required for hippocampal AMPA receptor-dependent synaptic transmission, synaptic plasticity, and working memory. J. Neurosci. *26*, 223–232.

26. Huang, Z., Shimazu, K., Woo, N.H., Zang, K., Müller, U., Lu, B., and Reichardt, L.F. (2006). Distinct roles of the beta 1-class integrins at the developing and the mature hippocampal excitatory synapse. J. Neurosci. *26*, 11208–11219.

27. Liu, X., Huai, J., Endle, H., Schlüter, L., Fan, W., Li, Y., Richers, S., Yurugi, H., Rajalingam, K., Ji, H., et al. (2016). PRG-1 regulates synaptic plasticity via intracellular PP2A/beta1-Integrin signaling. Dev. Cell. *38*, 275–290.

28. Warren, M.S., Bradley, W.D., Gourley, S.L., Lin, Y.C., Simpson, M.A., Reichardt, L.F., Greer, C.A., Taylor, J.R., and Koleske, A.J. (2012). Integrin beta1 signals through Arg to regulate postnatal dendritic arborization, synapse density, and behavior. J. Neurosci. *32*, 2824–2834.

29. Cingolani, L.A., and Goda, Y. (2008). Differential involvement of beta3 integrin in pre- and postsynaptic forms of adaptation to chronic activity deprivation. Neuron Glia Biol. *4*, 179–187.

30. Cingolani, L.A., Thalhammer, A., Yu, L.M.Y., Catalano, M., Ramos, T., Colicos, M.A., and Goda, Y. (2008). Activity-dependent regulation of synaptic AMPA receptor composition and abundance by beta3 integrins. Neuron *58*, 749–762.

31. McGeachie, A.B., Skrzypiec, A.E., Cingolani, L.A., Letellier, M., Pawlak, R., and Goda, Y. (2012). beta3 integrin is dispensable for conditioned fear and Hebbian forms of plasticity in the hippocampus. Eur. J. Neurosci. *36*, 2461–2469.

32. Nelson, S.B., and Valakh, V. (2015). Excitatory/inhibitory balance and circuit homeostasis in autism spectrum disorders. Neuron *87*, 684–698.

33. Uhlhaas, P.J., Pipa, G., Lima, B., Melloni, L., Neuenschwander, S., Nikolić, D., and Singer, W. (2009). Neural synchrony in cortical networks: history, concept and current status. Front. Integr. Neurosci. *3*, 17.

34. Bagni, C., and Zukin, R.S. (2019). A synaptic perspective of fragile X syndrome and autism spectrum disorders. Neuron *101*, 1070–1088.

35. Berry-Kravis, E.M., Lindemann, L., Jønch, A.E., Apostol, G., Bear, M.F., Carpenter, R.L., Crawley, J.N., Curie, A., Des Portes, V., Hossain, F., et al. (2018). Drug development for neurodevelopmental disorders: lessons learned from fragile X syndrome. Nat. Rev. Drug. Discov. *17*, 280–299.

36. Hagerman, R.J., Berry-Kravis, E., Hazlett, H.C., Bailey, D.B., Jr., Moine, H., Kooy, R.F., Tassone, F., Gantois, I., Sonenberg, N., Mandel, J.L., and Hagerman, P.J. (2017). Fragile X syndrome. Nat. Rev. Dis. Prim. *3*, 17065.

37. O'Connor, E.C., Bariselli, S., and Bellone, C. (2014). Synaptic basis of social dysfunction: a focus on postsynaptic proteins linking group-I mGluRs with AMPARs and NMDARs. Eur. J. Neurosci. *39*, 1114–1129.

38. Dana, H., Mohar, B., Sun, Y., Narayan, S., Gordus, A., Hasseman, J.P., Tsegaye, G., Holt, G.T., Hu, A., Walpita, D., et al. (2016). Sensitive red protein calcium indicators for imaging neural activity. Elife *5*, e12727.

39. Inoue, M., Takeuchi, A., Manita, S., Horigane, S.I., Sakamoto, M., Kawakami, R., Yamaguchi, K., Otomo, K., Yokoyama, H., Kim, R., et al. (2019). Rational engineering of XCaMPs, a multicolor GECI suite for in vivo imaging of complex brain circuit dynamics. Cell *177*, 1346–1360.e24.

40. Bianchi, R., Wong, R.K.S., and Merlin, L.R. (2012). Glutamate receptors in epilepsy: group I mGluR-mediated epileptogenesis. In Jasper's Basic Mechanisms of the Epilepsies, J.L. Noebels, M. Avoli, M.A. Rogawski, R.W. Olsen, and A.V. Delgado-Escueta, eds..

41. Cingolani, L.A., Vitale, C., and Dityatev, A. (2019). Intra- and extracellular pillars of a unifying framework for homeostatic plasticity: a crosstalk between metabotropic receptors and extracellular matrix. Front. Cell. Neurosci. *13*, 513.

42. Hu, J.H., Park, J.M., Park, S., Xiao, B., Dehoff, M.H., Kim, S., Hayashi, T., Schwarz, M.K., Huganir, R.L., Seeburg, P.H., et al. (2010). Homeostatic scaling requires group I mGluR activation mediated by Homer1a. Neuron *68*, 1128–1142.

43. Reiner, A., and Levitz, J. (2018). Glutamatergic signaling in the central nervous system: ionotropic and metabotropic receptors in concert. Neuron *98*, 1080–1098.

44. Jovanovic, M., Rooney, M.S., Mertins, P., Przybylski, D., Chevrier, N., Satija, R., Rodriguez, E.H., Fields, A.P., Schwartz, S., Raychowdhury, R., et al. (2015). Immunogenetics. Dynamic profiling of the protein life cycle in response to pathogens. Science *347*, 1259038.

45. Pozo, K., Cingolani, L.A., Bassani, S., Laurent, F., Passafaro, M., and Goda, Y. (2012). beta3 integrin interacts directly with GluA2 AMPA receptor subunit and regulates AMPA receptor expression in hippocampal neurons. Proc. Natl. Acad. Sci. USA *109*, 1323–1328.

46. Chavis, P., and Westbrook, G. (2001). Integrins mediate functional pre- and postsynaptic maturation at a hippocampal synapse. Nature *411*, 317–321.

47. Hennekinne, L., Colasse, S., Triller, A., and Renner, M. (2013). Differential control of thrombospondin over synaptic glycine and AMPA receptors in spinal cord neurons. J. Neurosci. *33*, 11432–11439.

48. Jones, E.V., Bernardinelli, Y., Tse, Y.C., Chierzi, S., Wong, T.P., and Murai, K.K. (2011). Astrocytes control glutamate receptor levels at developing synapses through SPARC-beta-integrin interactions. J. Neurosci. *31*, 4154–4165.

49. Shi, Y., and Ethell, I.M. (2006). Integrins control dendritic spine plasticity in hippocampal neurons through NMDA receptor and Ca2+/calmodulin-dependent protein kinase II-mediated actin reorganization. J. Neurosci. *26*, 1813–1822.

50. Xiao, X., Levy, A.D., Rosenberg, B.J., Higley, M.J., and Koleske, A.J. (2016). Disruption of coordinated presynaptic and postsynaptic maturation underlies the defects in hippocampal synapse stability and plasticity in Abl2/Arg-deficient mice. J. Neurosci. *36*, 6778–6791.

51. Bianchi, R., Chuang, S.C., Zhao, W., Young, S.R., and Wong, R.K.S. (2009). Cellular plasticity for group I mGluR-mediated epileptogenesis. J. Neurosci. *29*, 3497–3507.

52. Young, S.R., Chuang, S.C., Zhao, W., Wong, R.K.S., and Bianchi, R. (2013). Persistent receptor activity underlies group I mGluR-mediated cellular plasticity in CA3 neuron. J. Neurosci. *33*, 2526–2540.

53. Merlin, L.R. (2002). Differential roles for mGluR1 and mGluR5 in the persistent prolongation of epileptiform bursts. J. Neurophysiol. *87*, 621–625.

54. Mannaioni, G., Marino, M.J., Valenti, O., Traynelis, S.F., and Conn, P.J. (2001). Metabotropic glutamate receptors 1 and 5 differentially regulate CA1 pyramidal cell function. J. Neurosci. *21*, 5925–5934.

55. Chuang, S.C., Zhao, W., Bauchwitz, R., Yan, Q., Bianchi, R., and Wong, R.K.S. (2005). Prolonged epileptiform discharges induced by altered group I metabotropic glutamate receptor-mediated synaptic responses in hippocampal slices of a fragile X mouse model. J. Neurosci. *25*, 8048–8055.

56. Contractor, A., Klyachko, V.A., and Portera-Cailliau, C. (2015). Altered neuronal and circuit excitability in fragile X syndrome. Neuron *87*, 699–715.

57. Chavez, A., Scheiman, J., Vora, S., Pruitt, B.W., Tuttle, M., P R Iyer, E., Lin, S., Kiani, S., Guzman, C.D., Wiegand, D.J., et al. (2015). Highly efficient Cas9-mediated transcriptional programming. Nat. Methods *12*, 326–328.

58. Konermann, S., Brigham, M.D., Trevino, A.E., Joung, J., Abudayyeh, O.O., Barcena, C., Hsu, P.D., Habib, N., Gootenberg, J.S., Nishimasu, H., et al. (2015). Genome-scale transcriptional activation by an engineered CRISPR-Cas9 complex. Nature *517*, 583–588.

59. Matharu, N., Rattanasopha, S., Tamura, S., Maliskova, L., Wang, Y., Bernard, A., Hardin, A., Eckalbar, W.L., Vaisse, C., and Ahituv, N. (2019). CRISPR-mediated activation of a promoter or enhancer rescues obesity caused by haploinsufficiency. Science *363*, eaau0629.

60. Savell, K.E., Bach, S.V., Zipperly, M.E., Revanna, J.S., Goska, N.A., Tuscher, J.J., Duke, C.G., Sultan, F.A., Burke, J.N., Williams, D., et al. (2019). A neuron-optimized CRISPR/dCas9 activation system for Robust and specific gene regulation. eNeuro *6*. ENEURO.0495, 18.2019.

61. Tanenbaum, M.E., Gilbert, L.A., Qi, L.S., Weissman, J.S., and Vale, R.D. (2014). A protein-tagging system for signal amplification in gene expression and fluorescence imaging. Cell *159*, 635–646.

62. Zhou, H., Liu, J., Zhou, C., Gao, N., Rao, Z., Li, H., Hu, X., Li, C., Yao, X., Shen, X., et al. (2018). In vivo simultaneous transcriptional activation of multiple genes in the brain using CRISPR-dCas9-activator transgenic mice. Nat. Neurosci. *21*, 440–446.

63. Perez-Pinera, P., Kocak, D.D., Vockley, C.M., Adler, A.F., Kabadi, A.M., Polstein, L.R., Thakore, P.I., Glass, K.A., Ousterout, D.G., Leong, K.W., et al. (2013). RNA-guided gene activation by CRISPR-Cas9-based transcription factors. Nat. Methods *10*, 973–976.

64. Wu, X., Kriz, A.J., and Sharp, P.A. (2014). Target specificity of the CRISPR-Cas9 system. Quant. Biol. *2*, 59–70.

65. Kuscu, C., Arslan, S., Singh, R., Thorpe, J., and Adli, M. (2014). Genome-wide analysis reveals characteristics of off-target sites bound by the Cas9 endonuclease. Nat. Biotechnol. *32*, 677–683.

66. Belgard, T.G., Marques, A.C., Oliver, P.L., Abaan, H.O., Sirey, T.M., Hoerder-Suabedissen, A., García-Moreno, F., Molnár, Z., Margulies, E.H., and Ponting, C.P. (2011). A transcriptomic atlas of mouse neocortical layers. Neuron *71*, 605–616.

67. Willsey, A.J., Sanders, S.J., Li, M., Dong, S., Tebbenkamp, A.T., Muhle, R.A., Reilly, S.K., Lin, L., Fertuzinhos, S., Miller, J.A., et al. (2013). Coexpression networks implicate human midfetal deep cortical projection neurons in the pathogenesis of autism. Cell *155*, 997–1007.

68. Friedland, A.E., Baral, R., Singhal, P., Loveluck, K., Shen, S., Sanchez, M., Marco, E., Gotta, G.M., Maeder, M.L., Kennedy, E.M., et al. (2015). Characterization of Staphylococcus aureus Cas9: a smaller Cas9 for all-in-one adeno-associated virus delivery and paired nickase applications. Genome. Biol. *16*, 257.

69. Bikbaev, A., Frischknecht, R., and Heine, M. (2015). Brain extracellular matrix retains connectivity in neuronal networks. Sci. Rep. *5*, 14527.

70. Korotchenko, S., Cingolani, L.A., Kuznetsova, T., Bologna, L.L., Chiappalone, M., and Dityatev, A. (2014). Modulation of network activity and induction of homeostatic synaptic plasticity by enzymatic removal of heparan sulfates. Philos. Trans. R. Soc. Lond. B, Biol. Sci. *369*, 20140134.

71. Vedunova, M., Sakharnova, T., Mitroshina, E., Perminova, M., Pimashkin, A., Zakharov, Y., Dityatev, A., and Mukhina, I. (2013). Seizure-like activity in hyaluronidase-treated dissociated hippocampal cultures. Front. Cell. Neurosci. *7*, 149.

72. Stellwagen, D., and Malenka, R.C. (2006). Synaptic scaling mediated by glial TNF-alpha. Nature *440*, 1054–1059.

73. Hahn, G., Ponce-Alvarez, A., Deco, G., Aertsen, A., and Kumar, A. (2019). Portraits of communication in neuronal networks. Nat. Rev. Neurosci. *20*, 117–127.

74. Silberberg, G., Grillner, S., LeBeau, F.E.N., Maex, R., and Markram, H. (2005). Synaptic pathways in neural microcircuits. Trends. Neurosci. *28*, 541–551.

75. Oliet, S.H., Malenka, R.C., and Nicoll, R.A. (1997). Two distinct forms of long-term depression coexist in CA1 hippocampal pyramidal cells. Neuron *18*, 969–982.

76. Ango, F., Prézeau, L., Muller, T., Tu, J.C., Xiao, B., Worley, P.F., Pin, J.P., Bockaert, J., and Fagni, L. (2001). Agonist-independent activation of metabotropic glutamate receptors by the intracellular protein Homer. Nature *411*, 962–965.

77. Ronesi, J.A., Collins, K.A., Hays, S.A., Tsai, N.P., Guo, W., Birnbaum, S.G., Hu, J.H., Worley, P.F., Gibson, J.R., and Huber, K.M. (2012). Disrupted Homer scaffolds mediate abnormal mGluR5 function in a mouse model of fragile X syndrome. Nat. Neurosci. *15*, 431–440.

78. Stachniak, T.J., Sylwestrak, E.L., Scheiffele, P., Hall, B.J., and Ghosh, A. (2019). Elfn1-Induced constitutive activation of mGluR7 determines frequency-dependent recruitment of somatostatin interneurons. J. Neurosci. *39*, 4461–4474.

79. Tomioka, N.H., Yasuda, H., Miyamoto, H., Hatayama, M., Morimura, N., Matsumoto, Y., Suzuki, T., Odagawa, M., Odaka, Y.S., Iwayama, Y., et al. (2014). Elfn1 recruits presynaptic mGluR7 in trans and its loss results in seizures. Nat. Commun. *5*, 4501.

80. Giuffrida, R., Musumeci, S., D'Antoni, S., Bonaccorso, C.M., Giuffrida-Stella, A.M., Oostra, B.A., and Catania, M.V. (2005). A reduced number of metabotropic glutamate subtype 5 receptors are associated with constitutive homer proteins in a mouse model of fragile X syndrome. J. Neurosci. *25*, 8908–8916.

81. Guo, W., Molinaro, G., Collins, K.A., Hays, S.A., Paylor, R., Worley, P.F., Szumlinski, K.K., and Huber, K.M. (2016). Selective disruption of metabotropic glutamate receptor 5-homer interactions mimics phenotypes of fragile X syndrome in mice. J. Neurosci. *36*, 2131–2147.

82. Maurin, T., Lebrigand, K., Castagnola, S., Paquet, A., Jarjat, M., Popa, A., Grossi, M., Rage, F., and Bardoni, B. (2018). HITS-CLIP in various brain areas reveals new targets and new modalities of RNA binding by fragile X mental retardation protein. Nucleic. Acids. Res. *46*, 6344–6355.

83. Dohn, M.R., Kooker, C.G., Bastarache, L., Jessen, T., Rinaldi, C., Varney, S., Mazalouskas, M.D., Pan, H., Oliver, K.H., Velez Edwards, D.R., et al. (2017). The gain-of-function integrin beta3 Pro33 variant Alters the serotonin system in the mouse brain. J. Neurosci. *37*, 11271–11284.

84. Kumar, N., Stanford, W., de Solis, C., Aradhana, Abraham, N.D., Dao, T.M.J., Thaseen, S., Sairavi, A., Gonzalez, C.U., and Ploski, J.E. (2018). The development of an AAV-based CRISPR SaCas9 genome editing system that can Be delivered to neurons. Front. Mol. Neurosci. *11*, 413.

85. Kleinstiver, B.P., Prew, M.S., Tsai, S.Q., Nguyen, N.T., Topkar, V.V., Zheng, Z., and Joung, J.K. (2015). Broadening the targeting range of Staphylococcus aureus CRISPR-Cas9 by modifying PAM recognition. Nat. Biotechnol. *33*, 1293–1298.

86. Riccardi, S., Cingolani, L.A., and Jaudon, F. (2022). CRISPR-mediated activation of alphaV integrin subtypes promotes neuronal differentiation of neuroblastoma Neuro2a cells. Front. Genome Ed. *4*, 846669.

87. Thalhammer, A., Jaudon, F., and Cingolani, L.A. (2018). Combining optogenetics with Artificial microRNAs to characterize the effects of gene knockdown on presynaptic function within intact neuronal circuits. J. Vector. Ecol. 57223.

88. Thalhammer, A., Contestabile, A., Ermolyuk, Y.S., Ng, T., Volynski, K.E., Soong, T.W., Goda, Y., and Cingolani, L.A. (2017). Alternative splicing of P/Q-Type Ca2+ channels shapes presynaptic plasticity. Cell Rep. *20*, 333–343.

89. Ferrante, D., Sterlini, B., Prestigio, C., Marte, A., Corradi, A., Onofri, F., Tortarolo, G., Vicidomini, G., Petretto, A., Muià, J., et al. (2021). PRRT2 modulates presynaptic Ca(2+) influx by interacting with P/Q-type channels. Cell Rep. *35*, 109248.

90. Li, H., and Durbin, R. (2009). Fast and accurate short read alignment with Burrows-Wheeler transform. Bioinformatics *25*, 1754–1760.

91. Zhang, Y., Liu, T., Meyer, C.A., Eeckhoute, J., Johnson, D.S., Bernstein, B.E., Nusbaum, C., Myers, R.M., Brown, M., Li, W., and Liu, X.S. (2008). Model-based analysis of ChIP-seq (MACS). Genome. Biol. *9*, R137.

92. Mazalouskas, M., Jessen, T., Varney, S., Sutcliffe, J.S., Veenstra-VanderWeele, J., Cook, E.H., Jr., and Carneiro, A.M.D. (2015). Integrin beta3 haploinsufficiency modulates serotonin transport and Antidepressant-sensitive behavior in mice. Neuropsychopharmacology *40*, 2015–2024.

93. Arsic, N., Zacchigna, S., Zentilin, L., Ramirez-Correa, G., Pattarini, L., Salvi, A., Sinagra, G., and Giacca, M. (2004). Vascular endothelial growth factor stimulates skeletal muscle regeneration in vivo. Mol. Ther. *10*, 844–854.

94. Kim, J.Y., Ash, R.T., Ceballos-Diaz, C., Levites, Y., Golde, T.E., Smirnakis, S.M., and Jankowsky, J.L. (2013). Viral transduction of the neonatal brain delivers controllable genetic mosaicism for visualising and manipulating neuronal circuits in vivo. Eur. J. Neurosci. *37*, 1203–1220.

OMTN, Volume *29*

Supplemental information

CRISPR-mediated activation of autism gene *Itgb3* restores cortical network excitability via mGluR5 signaling

Fanny Jaudon, Agnes Thalhammer, Lorena Zentilin, and Lorenzo A. Cingolani

SUPPLEMENTAL MATERIAL

SUPPLEMENTAL FIGURES AND TABLES

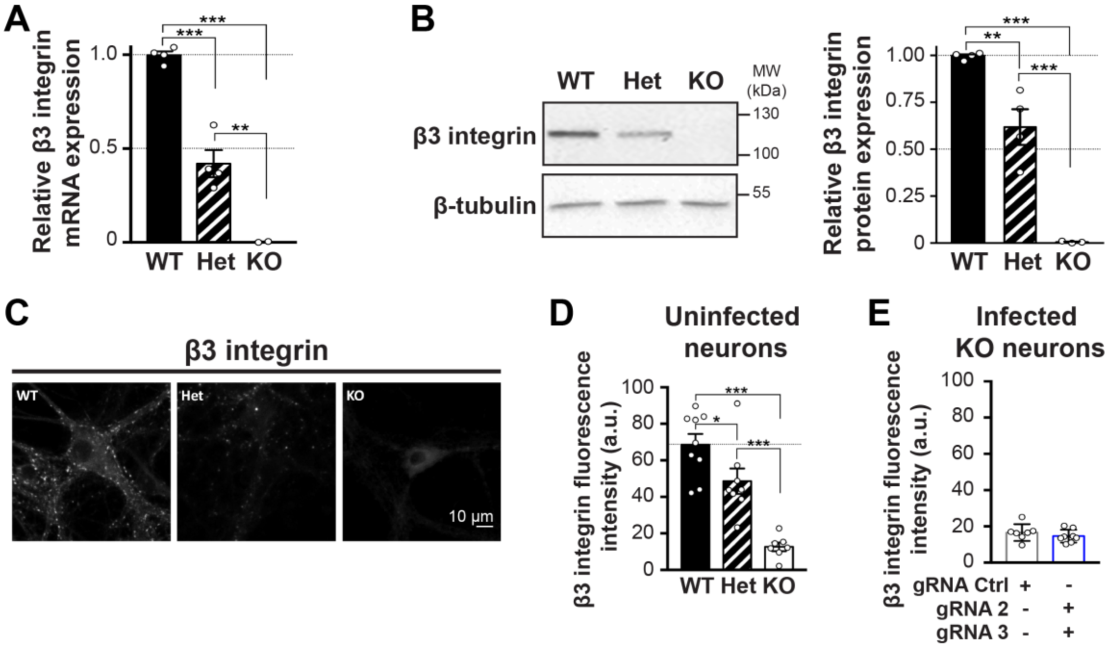

Figure S1. β3 integrin expression in primary cortical neurons. (A) Expression of β3 integrin mRNA in mouse primary cortical neurons at 16 DIV. Values are normalized to WT samples within the same RT-qPCR plate (n=4, 4 and 2 independent cultures for WT, *Itgb3* Het and KO, respectively). **(B)** Membrane protein fractions from mouse primary cortical neurons were analysed by Western blotting at 16 DIV. Left, representative immunoblots for β3 integrin; β-tubulin was used as a loading control. Right, quantification of immuno-reactive bands; band intensities were normalized to WT within the same membrane (n=4, 4 and 3 independent cultures for WT, Het and KO, respectively). **(C)** Representative confocal images of WT, *Itgb3* Het and KO primary cortical neurons stained for β3 integrin at 16 DIV. **(D)** Quantification of experiments as in (C; n=9, 8 and 7 images for WT, Het and KO, respectively). **(E)** Infection with CRISPRa and gRNAs 2+3 does not increase β3 integrin expression in *Itgb3* KO cultures (n=7 and 8 images for gRNA Control and gRNAs 2+3, respectively). Data are presented as mean±SEM; dots represent individual values (*p<0.05, **p<0.01, ***p<0.001, one-way ANOVA followed by Tukey post-test for panels A, B and D; p=0.37, unpaired Student's t-test for panel E).

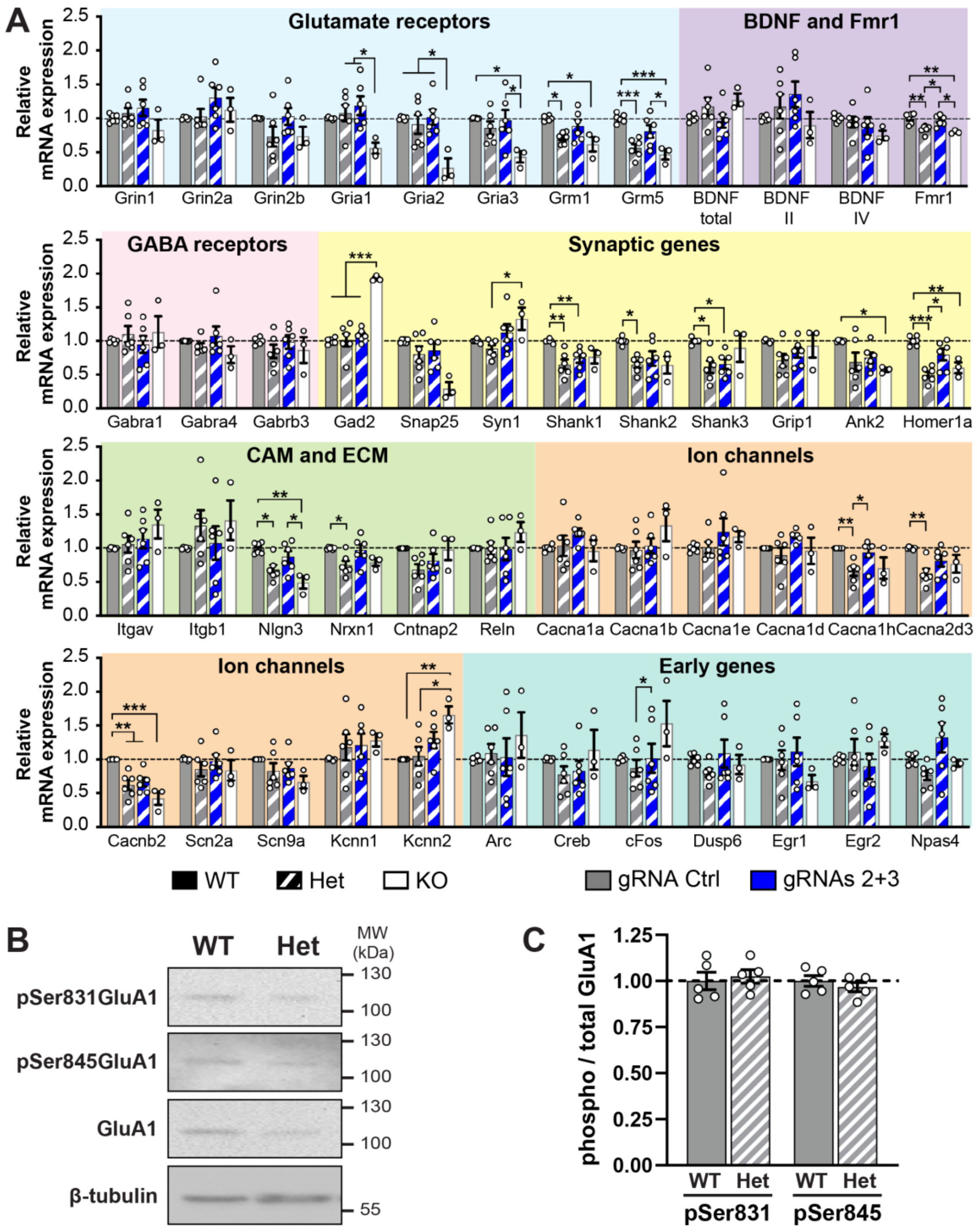

Figure S2. Regulation of neuronal gene expression by β3 integrin. (A) RT-qPCR quantification of mRNA expression for 48 neuronal genes (grouped into functional categories as labelled) in WT, *Itgb3* Het and KO cortical neurons expressing the indicated constructs (*p<0.05, **p<0.01, ***p<0.001, one-way ANOVA followed by Tukey post-test; n=6, 6 and 3 independent cultures for WT, Het and KO, respectively; 2 technical replicates each). **(B)** Representative Western blots of membrane-enriched fractions from WT and *Itgb3* Het cortical neurons. **(C)** Quantification of experiments as in (B) showing that phosphorylation levels of GluA1 are not altered in Het neurons (unpaired Student's t-test; n=5 independent cultures). Data are shown as mean±SEM; dots represent individual values.

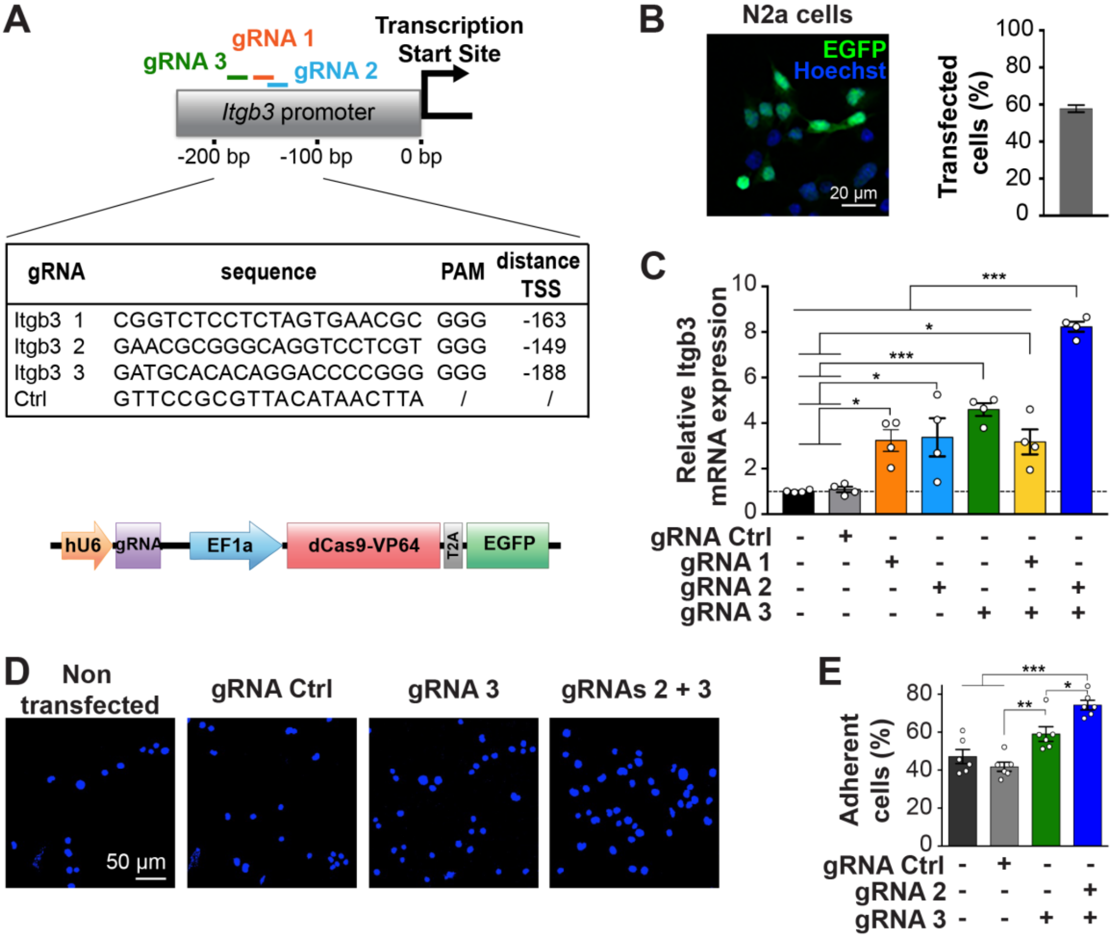

Figure S3. CRISPR/dCas9-mediated enhancement of β3 integrin expression in N2a cells. (A) Top, gRNA sequences and position of their targets on the *Itgb3* promoter. Bottom, construct used for transfecting murine N2a cells, containing a cassette for expressing gRNA and one for expressing dCas9-VP64 and EGFP. **(B)** Representative image of transfected N2a cells (left) and quantification of transfection efficiency (right). **(C)** Quantification of β3 integrin mRNA levels in N2a cells 24 hours after transfection with the indicated constructs. mRNA expression values were normalized to those of non-transfected samples within the same RT-qPCR plate (n=4 independent cultures each; 2 technical replicates per culture). **(D-E)** Cell adhesion assay for N2a cells transfected with the indicated constructs and plated onto fibronectin-coated coverslips. Representative images of adherent cells stained with Hoechst (D) and quantification of the percentage of adherent cells (E; n=6 each from 3 independent cultures). Data are presented as mean±SEM; dots represent individual values (*p<0.05, **p<0.01, ***p<0.001, one-way ANOVA followed by Tukey post-test).

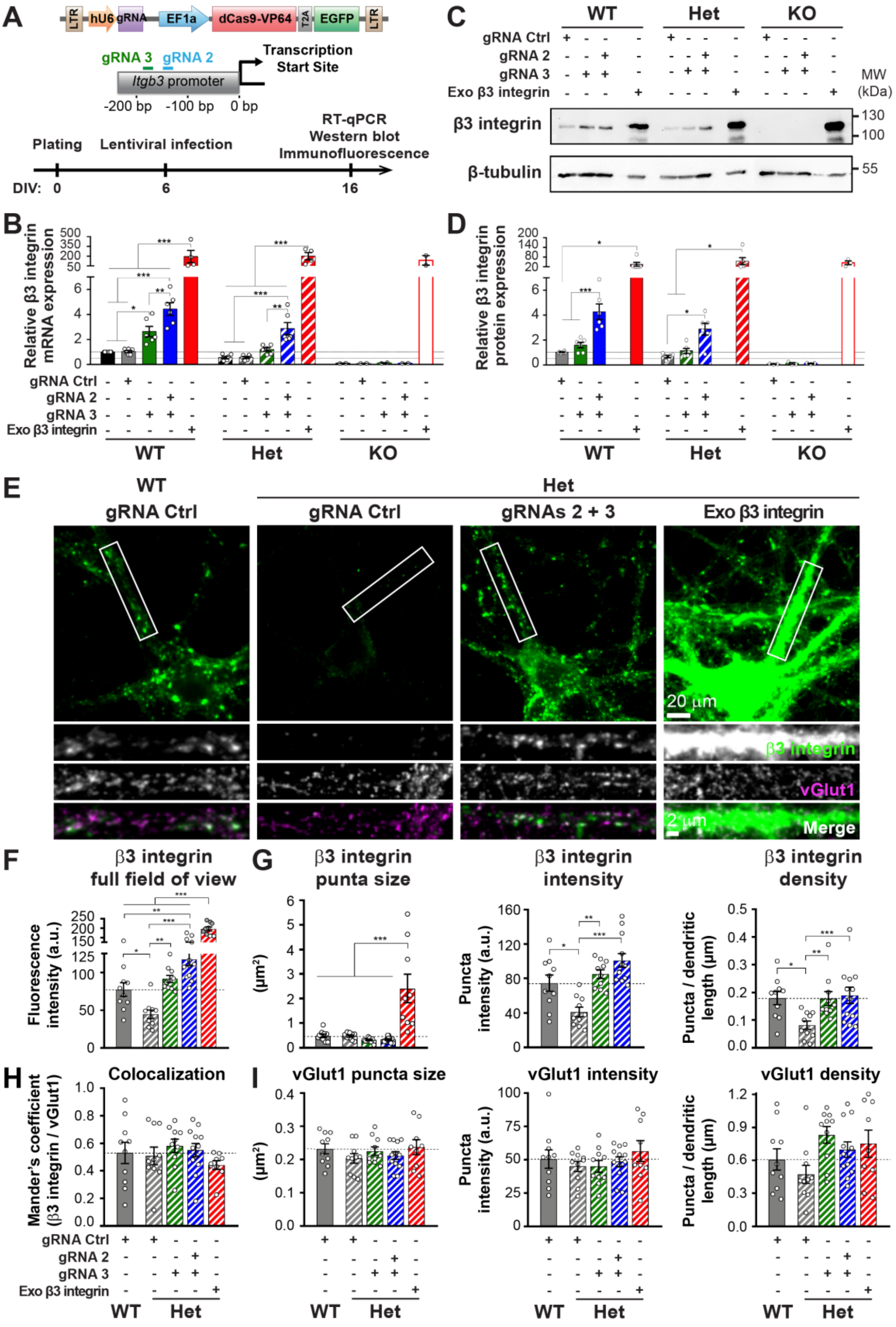

Figure S4. Rescue of β3 integrin expression by CRISPRa. (A) Scheme of lentiviral construct, gRNA targets on the *Itgb3* promoter and experimental timeline. **(B)** RT-qPCR quantification of β3 integrin mRNA expression in WT, *Itgb3* Het and KO cortical neurons transduced with the indicated constructs. (n=6, 6 and

2 independent cultures for WT, Het and KO, respectively; 2 technical replicates each). **(C)** Representative Western blots of membrane fractions. **(D)** Quantification of experiments as in (C; n=6, 6 and 3 independent cultures for WT, Het and KO, respectively; 2 technical replicates each). CRISPRa, but not over-expression of exogenous β3 integrin, rescues *Itgb3* gene dosage in *Itgb3* het neurons at both the mRNA and protein level. **(E)** Representative confocal images of primary cortical neurons from WT and Het cultures expressing the indicated constructs. β3 integrin and the presynaptic marker vGlut1 are shown in false colors; infection was confirmed by EGFP. **(F)** Quantification of β3 integrin fluorescence intensity for the full field of view for experiments as in (E), indicating that CRISPRa elevates β3 integrin expression in Het to WT values while exogenous expression of β3 integrin increases several-fold the signal for this protein (the fluorescence intensity for Het+exogenous β3 integrin is a lower estimate because of pixel saturation; gray filled circles indicate pixel saturation >30%). **(G)** Left panel, dendritic puncta size for β3 integrin; expression of exogenous β3 integrin resulted in large dendritic areas of saturated signal, which were no further analyzed. Middle and right panels, quantification of the effects of the indicated gRNAs on dendritic puncta intensity and number for β3 integrin. **(H)** Mander's co-localization coefficient of β3 integrin with vGlut1. **(I)** Quantification of dendritic puncta size, intensity and number for vGlut1 (n=10, 10, 10, 12 and 9 from 3 independent cultures each for WT+gRNA Ctrl, Het+gRNA Ctrl, Het+gRNA 3, Het+gRNAs 2+3 and Het+exogenous β3 integrin, respectively). Data are presented as mean±SEM; dots represent individual values (*$p<0.05$, **$p<0.01$, ***$p<0.001$, one-way ANOVA followed by Tukey post-test).

A

	Off-target sequences	Number of mismatches	Genomic coordinates (GRCm38.p4 C57BL/6J)	Position	Gene
**gRNA 2**	**GAACGCGGGCAGGTCCTCGT**				
#1	GAGCGCTGGCAGGTCCTCGG	3	Chr5: 128607080 - 128607099	Intergenic	
#2	GGATGGGGGAAGGTCCTCGT	4	Chr14: 65726265 - 65726284	Intronic	Scara5
#3	GACCGCAGAGAGGTCCTCGT	4	Chr7: 135803303 - 135803322	Intergenic	
#4	GATGGCAGGCAGTTCCTCGT	4	Chr6: 86380681 - 86380700	Intergenic	
#5	GGAGGGGGGCAGTTCCTCGT	4	Chr4: 44576073 - 44576092	Intronic	Pax5
#6	GAACTCCGACCGGTCCTCGT	4	Chr13: 34002574 - 34002593	Exonic	Serpinb6a
#7	AAACCCGGGAAGGTCCTAGT	4	Chr16: 89832908 - 89832927	Intronic	Tiam1
#8	GAAAGAGTGCAGGTCCTAGT	4	Chr11: 60178341 - 60178360	Intergenic	
#9	GAACACAGGCAGTTCCTCGG	4	Chr14: 64286180 - 64286199	Intronic	Msra
#10	GACCGAGGGCTGGTCCTCAT	4	Chr2: 58176765 - 58176784	Exonic	Gm13546
**gRNA 3**	**GATGCACACAGGACCCCGGG**				
#1	GATGCCCACAGGGCCCCGGG	2	Chr4: 108941267 - 108941286	Exonic	Rab3B
#2	CCTGAACGCAGGACCCCGGG	4	Chr12: 105964060 - 105964079	Intergenic	
#3	CTTGCACACAGGACCCTGGG	3	Chr6: 146208088 - 146208107	Intronic	Itpr2
#4	GGAGAAGACAGGACCCCGGG	4	Chr17: 68274174 - 68274193	Intergenic	
#5	CCTGTACACAGCACCCCGGG	4	Chr8: 122480377 - 122480396	Exonic	Ctu2
#6	GCTGAACTCAGCACCCCGGG	4	Chr6: 39621848 - 39621867	Intronic	Braf
#7	GATGAACAGAGGACCCGGGG	3	Chr17: 45669894 - 45669913	Exonic	Tmem63B
#8	GACACACCCAGGACCCCGGA	4	Chr1: 91602261 - 91602280	Intergenic	
#9	GAGGTACTCAGGACCCTGGG	4	Chr3: 134181351 - 134181370	Intergenic	
#10	CCTACACACAGGCCCCCGGG	4	Chr12: 117153148 - 117153167	Intronic	Ptprn2

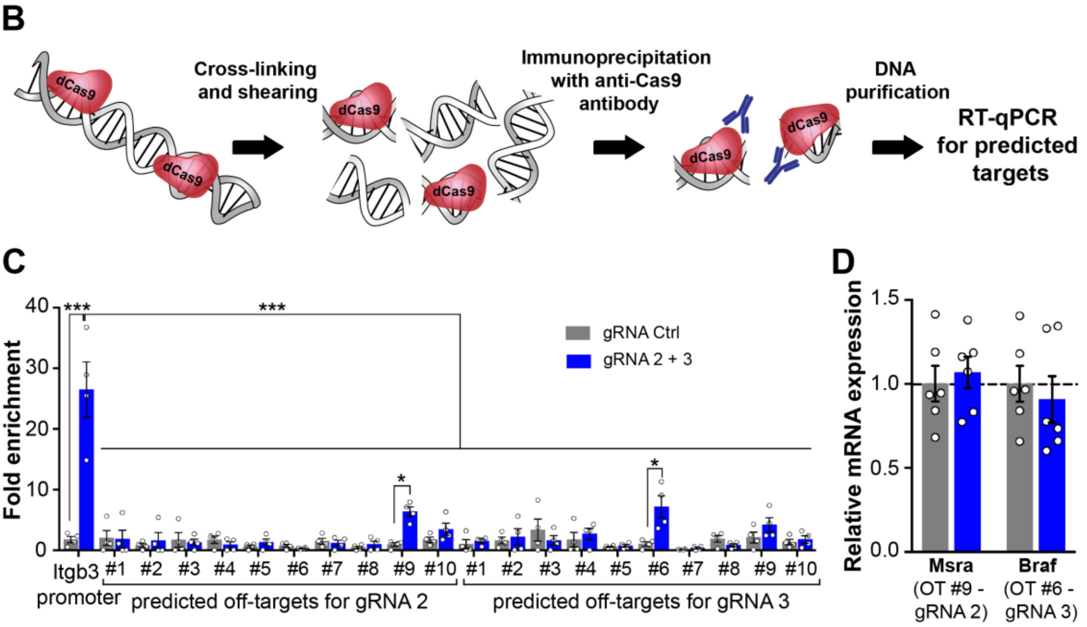

Figure S5. Target specificity of CRISPRa for β3 integrin in primary cortical neurons. (A) List of top-ten predicted off-targets for gRNAs 2 and 3 (http://crispr.mit.edu and https://crispr.cos.uni-heidelberg.de). The mismatches between the predicted off-targets and the on-target sequence are highlighted in red. Number of mismatches, genomic coordinates, position and name of potentially targeted genes are indicated. Scara5, scavenger receptor class A, member 5; Pax5, paired box protein 5; Serpinb6a, serine/cysteine peptidase inhibitor, clade B, member 6a; Tiam1, T cell lymphoma invasion and metastasis 1; Msra, mitochondrial peptide methionine sulfoxide reductase; Gm13546, predicted gene 13546, long non-coding RNA; Rab3B, member RAS oncogene family Rab3b; Itpr2, inositol 1,4,5-triphosphate receptor 2, transcript variant 2; Ctu2, cytosolic thiouridylase subunit 2; Braf, Braf transforming gene; Tmem63B,

transmembrane protein 63b; Ptprn2, protein tyrosine phosphatase, receptor type, N polypeptide 2. **(B)** ChIP-qPCR workflow. dCas9 co-expressed with gRNA Ctrl or gRNAs 2+3 is allowed to bind to chromatin. After cross-linking, the chromatin is sheared, immune-precipitated with an anti-Cas9 antibody and subjected to RT-qPCR. **(C)** Fold enrichment of dCas9 at on- and predicted off-target sites was calculated over an IgG control IP (*$p<0.05$, ***$p<0.001$, two-way ANOVA followed by Tukey post-test, n=4 independent cultures, 2 technical replicates each). **(D)** RT-qPCR quantification of mRNA expression for the two predicted off-target genes displaying significant dCas9 binding. Expression of both genes is not altered by dCas9-VP64 binding ($p\geq0.60$, unpaired Student's t-test, n=6 independent cultures, 2 technical replicates each). Data are presented as mean±SEM; dots represent individual values.

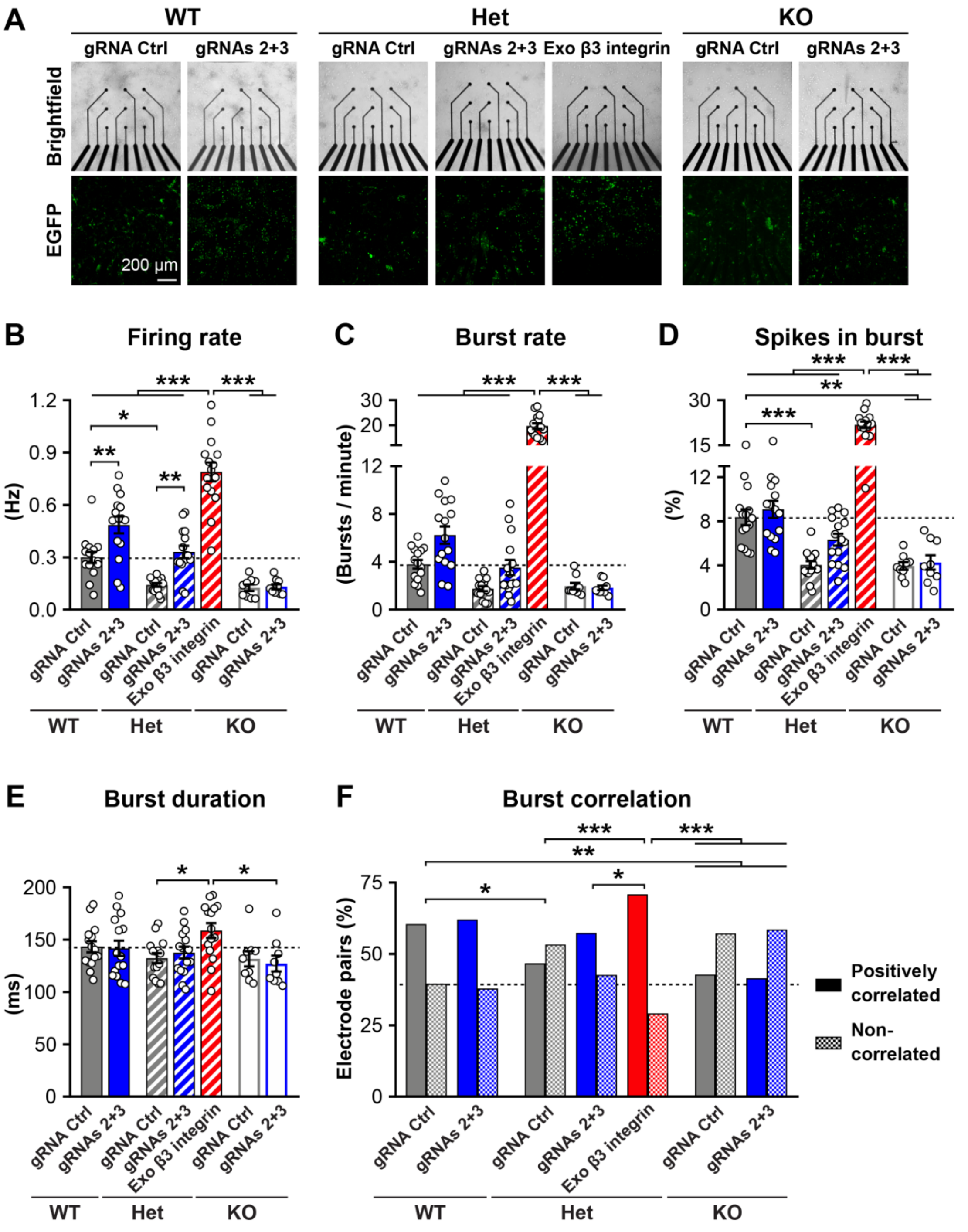

Figure S6. Further characterization of the effects of β3 integrin on network excitability. (A) Top, WT, *Itgb3* Het and KO cortical neurons expressing the indicated constructs plated on MEAs. Bottom, transduction efficiency was confirmed by EGFP expression. **(B-E)** Quantification of experiments as in Fig 5C-G. CRISPRa is effective in WT and *Itgb3* Het cultures but not *Itgb3* KO cultures (*$p<0.05$, **$p<0.01$, ***$p<0.001$, one-way ANOVA followed by Tukey post-test, n=15 each from 5 independent cultures). Data are presented as mean±SEM; dots represent individual values. **(F)** Quantification of Pearson's correlation coefficients (r) for burst activity as in Fig 5H, I. All electrode pairs exhibited a positive r. The graph shows the percentage of r with a p-value <0.05 (Positively

correlated) and a p-value >0.05 (Non-correlated; *$p<0.05$, **$p<0.01$, ***$p<0.001$, Chi-square test; n=473, 493, 447, 455, 532, 257 and 279 pairs for WT+gRNA Ctrl, WT+gRNAs 2+3, Het+gRNA Ctrl, Het+gRNAs 2+3, Het+exogenous β3 integrin, KO+gRNA Ctrl and KO+gRNAs 2+3, respectively).

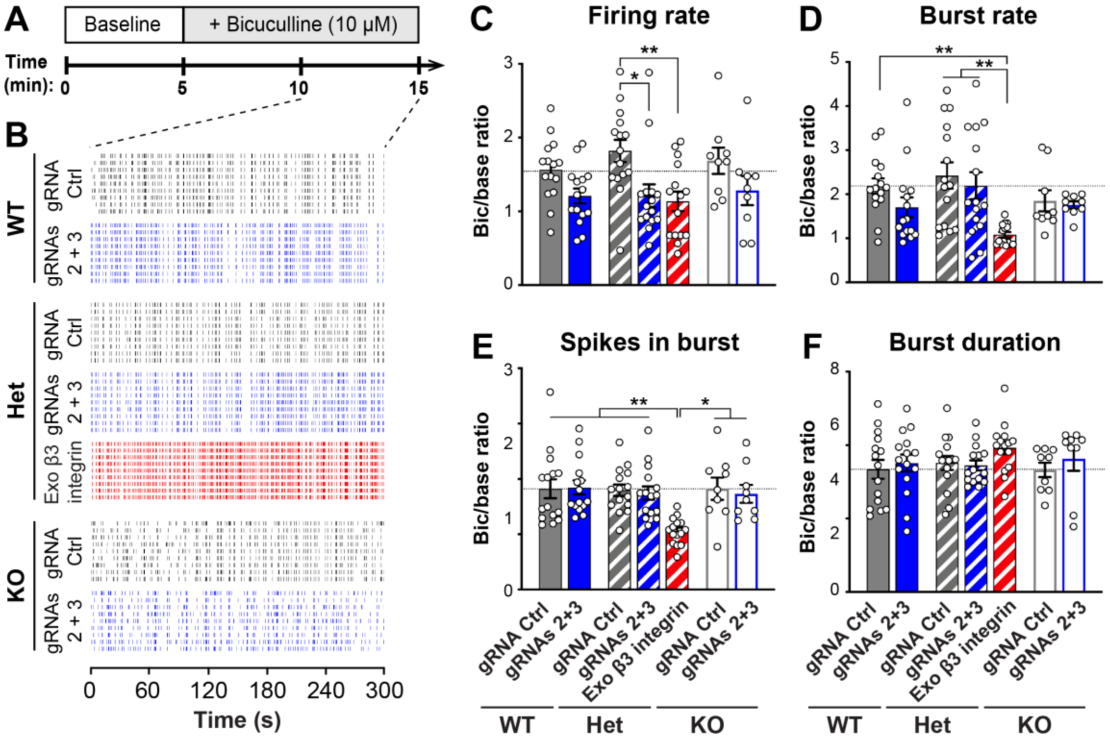

Figure S7. Effects of *Itgb3* expression levels on the bicuculline-dependent increase in network excitability. (A) Experimental timeline for bicuculline application (10 μM) in MEA experiments. **(B)** Representative raster plots of network activity after bicuculline application in WT, *Itgb3* Het and KO cultures expressing the indicated constructs. **(C-F)** Quantification of the bicuculline effects in experiments as in (A-B). Values are normalized to baseline for each recording (*$p<0.05$, **$p<0.01$, one-way ANOVA followed by Tukey post-test; n=15, 15 and 9 for WT, Het and KO, respectively; 5 independent cultures). Data are presented as mean±SEM; dots represent individual values.

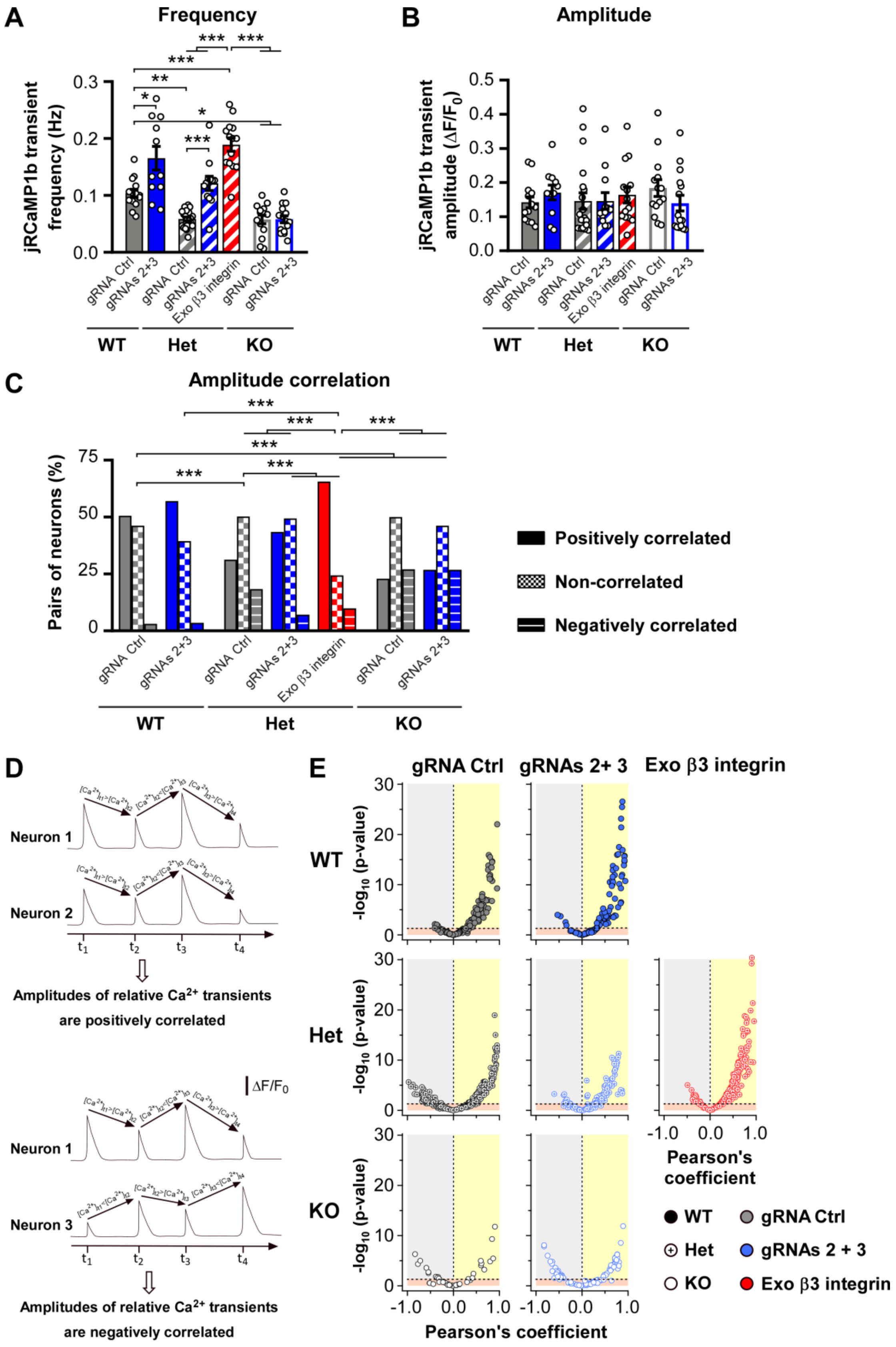

Figure S8. Further characterization of the effects of β3 integrin on jRCaMP1b fluorescence transients. (A-B) Quantification of experiments as in Fig 6A-C. CRISPRa is effective in WT and *Itgb3* Het cultures but not *Itgb3* KO cultures

(*p<0.05, **p<0.01, ***p<0.001, one-way ANOVA followed by Tukey post-test, n=14, 11, 20, 12, 14, 14 and 15 fields of view for WT+gRNA Ctrl, WT+gRNAs 2+3, Het+gRNA Ctrl, Het+gRNAs 2+3, Het+exogenous β3 integrin, KO+gRNA Ctrl and KO+gRNAs 2+3, respectively; 4-5 independent cultures). Data are shown as mean±SEM; dots represent individual values. **(C)** Quantification of Pearson's correlation coefficients (*r*) for fluorescence transient amplitudes as in Fig 6D, E. The graph shows the percentage of positive *r* with a p-value <0.05 (Positively correlated), negative *r* with a p-value <0.05 (Negatively correlated) and *r* with a p-value >0.05 (Non-correlated;, ***p<0.001, Chi-square test; n=158, 114, 396, 168, 233, 48 and 119 pairs for WT+gRNA Ctrl, WT+gRNAs 2+3, Het+gRNA Ctrl, Het+gRNAs 2+3, Het+exogenous β3 integrin, KO+gRNA Ctrl and KO+gRNAs 2+3, respectively). **(D)** Graphical illustration of the correlation analysis for fluorescence transient amplitudes. Assuming steady state conditions in the jRCaMP1b experiments, differences in amplitude of fluorescence signals are indicative of relative differences in Ca^{2+} transients at different time points within one neuron. Although it is not possible to compare directly differences in amplitude of fluorescence transients across neurons (e.g. because of differences in jRCaMP1b expression), fluorescence amplitude profiles between pairs of neurons can be compared to reveal positive (top) or negative (bottom) correlation in Ca^{2+} transients. **(E)** Volcano plots of all data points used for panel (C) and Fig 6E. The Pearson's correlation coefficient of fluorescence transient amplitudes for pairs of neurons is plotted against the $-\log_{10}$ of its p-value. Grey, pink and yellow backgrounds indicate negative, non-significant and positive correlation, respectively.

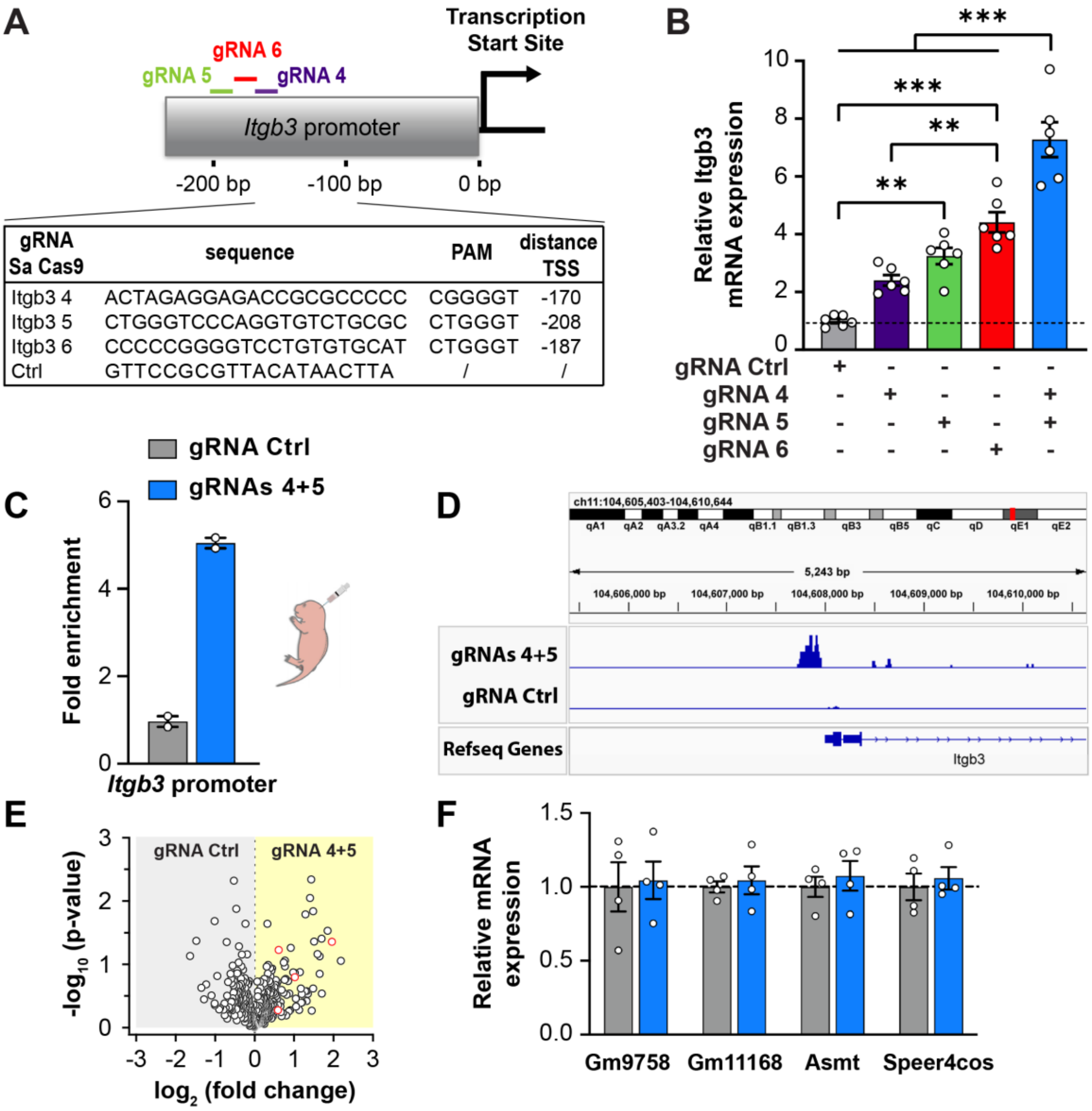

Figure S9. Efficacy and specificity of *S. aureus* Cas9-mediated CRISPRa for β3 integrin. (A) gRNA sequences for Sa-Cas9 and position of their targets on the *Itgb3* promoter. **(B)** Quantification of *Itgb3* mRNA levels in N2a cells 72 hours after transfection with Sa-dCas9-VPR and the indicated constructs. mRNA expression values were normalized to those of gRNA Ctrl-expressing samples within the same RT-qPCR plate (**p<0.01, ***p<0.001, one-way ANOVA followed by Tukey post-test; n=3 independent cultures each; 2 technical replicates per culture). **(C)** *In vivo* fold enrichment of Sa-dCas9-VPR at the *Itgb3* promoter calculated over an IgG control IP (n=2 cortices per condition). **(D)** Integrative genomics viewer snapshot of the peak at the *Itgb3* promoter in *in vivo* ChIP-seq experiments. **(E)** Volcano plot of the genome-wide off-target peaks for *in vivo* ChIP-seq experiments. The $\log_2$ fold change of gRNAs 4 + 5 over gRNA Ctrl (n = 2 cortices per condition) is plotted against the $-\log_{10}$ of the p-value for each peak (n = 494). None of the peaks is significantly enriched in either gRNA Ctrl (grey background) or gRNAs 4 + 5 condition (yellow background; no p-value was recognized as 'discovery' using the two-stage linear step-up procedure of

Benjamini, Krieger and Yekutieli for the false discovery rate with Q = 1 or 5%). Red circles indicate genes with a peak within 1 kb of their TSS. **(F)** RT-qPCR quantification of mRNA expression for the four genes marked in red in (E) indicates no change in their expression ($p \geq 0.56$, unpaired Student's t-test, n=4 cortices per group). *Gm9758*, predicted gene 9758; *Gm11168*, predicted gene 11168; *Asmt*, acetylserotonin O-methyltransferase; *Speer4cos*, spermatogenesis associated glutamate (E)-rich protein 4C. Data are presented as mean±SEM; dots represent individual values.

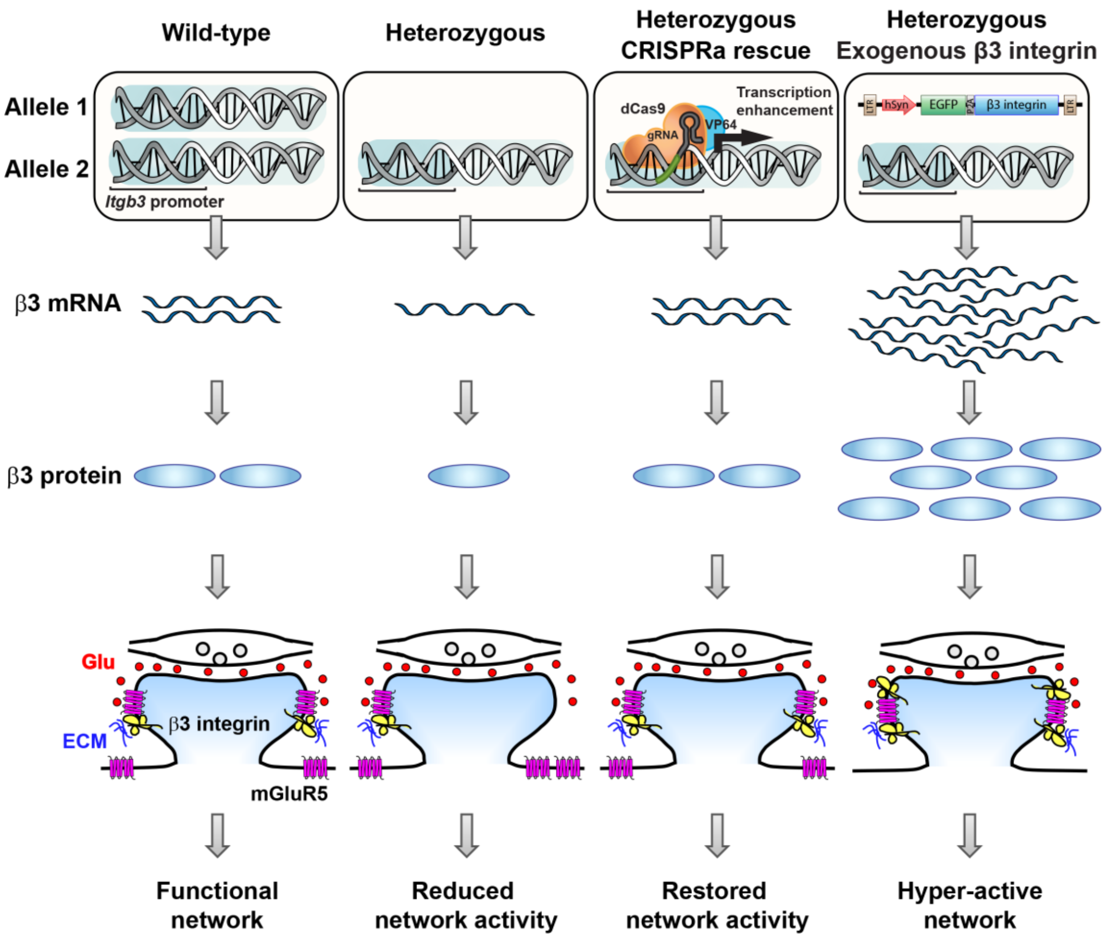

Figure S10. Working model comparing the effects of CRISPRa and over-expression on β3 integrin signalling. In Het neurons, the remaining allele of *Itgb3* produces an amount of mRNA and protein for β3 integrin that is 50% of that in WT neurons, with a consequent reduction in synaptic mGluR5 signalling and network excitability. By enhancing expression of the remaining allele, CRISPRa restores β3 integrin levels back to WT values, thus precisely rebalancing mGluR5 expression and network activity. Exogenous β3 integrin fails to mimic WT conditions, leading to un-physiologically high mRNA and protein levels for β3 integrin that result in hyperactive networks.

Table S1. List of primers used

RT-qPCR primers Gene	GenBank Accession	Forward sequence (5' → 3')	Reverse sequence (5' → 3')
m.Itgb3	NM_016780.2	GGGCGTTGTTGTTGGAGAG	ACAAAGTCTCATCTGAGCACCAG
h.ITGB3	NM_000212.2	CATCTCTGGGGCTGATGACT	GAGCGGATTTTCCCGTAAGC
Grin1	NM_008169.3	AAACCAGGCCAATAAGCGAC	GCGTAGACCTGGCTAGAGAT
Grin2a	NM_008170.2	GGTCAGCTTGAAAACTGGGAAG	AGATGTACCCGCTCCCAATG
Grin2b	NM_008171.3	CCTCCTGTGTGAGAGGAAATCT	CTCCTGGGTTGGGAAGTTCA
Gria1	NM_001113325.2	TGTGTTTGTTCGGACCACAG	GAGCACTGGTCTTGTCCTTAC
Gria2	NM_001083806.2	ATGGTTGTCACCCTAACCGA	AACGCTCATTCCCTTCAAGC
Gria3	NM_016886.4	CTCAGCATTAGGAACGCCTG	TTCCCCCTTATCGTACCACC
Grm1	NM_016976.3	CTGATTCACACACCTTCGGG	CCAAACCCTAGGGGTGTTCT
Grm5	NM_001081414.2	AGCGCACCTGGTGATTTTAC	ATGGGAGGCTTCAGCATACA
Gabra1	NM_010250.5	AAAAGCGTGGTTCCAGAAAA	GCTGGTTGCTGTAGGAGCAT
Gabra4	NM_010251.2	AGAACTCAAAGGACGAGAAATTGT	TTCACTTCTGTAACAGGACCCC
Gabrb3	NM_008071.3	TGCATTGAAAGGTGCCATGT	TATGGTGCATGAGCCACTCT
Gad2	NM_008078.2	GGAATCTTTTCTCCTGGTGGC	ATCAAAAGCCCCATACACGG
Snap25	NM_011428.3	CCTAGTAGGTCTTGCACATACAC	GACAGAGCACACAGGACATTT
Syn1	NM_013680.4	AGCTCAACAAATCCCAGTCTCT	CGGATGGTCTCAGCTTTCAC
Shank1	NM_001034115.1	GCACCCTTTCTTTCTCTAGCC	TATGGGAGTATGCCTGGGTC
Shank2	NM_001081370.3	GAGGAACTCGTGGACAAAGC	GATTCGATGGCCACGTTCTC
Shank3	NM_021423.4	AGGAACTTGCTTCCATTCGG	AGTCAGCATCTGCAATGTCC
Grip1	NM_028736.2	GACTGGAGCGAACAGAACAG	GTGTTAGTGGGTTCTCGTGTC
Ank2	NM_178655.3	TCTGAACCCAGCGTTTTGTC	TCTCCGTGTACCATGGTTGT
Homer1a	NM_011982.4	AATTTAAGGAAGCTGCTCGGC	CCTGTGAAGGGGTACTGGTC
Cacna1a	NM_007578.3	CCTGATGATGACAAGACACC	TTCCAGCCTCAAAACAGAAG
Cacna1b	NM_001042528.2	TTGAGTACCTCACTCGGGAC	GTCGTATTCAGCCCAGACTC
Cacna1e	NM_009782.3	CCTGACTCGAGATTCCTCCAT	ATGCTGCTCTGTCATATTCTGC
Cacna1d	NM_028981.3	TGTGATGTGCCAGTAGGTGA	CACGTATCGGGTTGGTCTTG
Cacna1h	NM_021415.4	CCTGGACCTCTTCATCACCT	GTACTTAAGGGCCTCGTCCA
Cacna2d3	NM_009785.1	ATCCTGAGGAGAATGCAAGAGAG	TTATGTCTCCTATGTCGCACCA
Cacnb2	NM_023116.4	TAAGCCCAGTGCAAACAGTG	CGCATGGAAGGTACCACATC
Scn2a	NM_001099298.3	TGTTTGATGTGAGCGTGGTC	CCAAGTCCCACGTTGTCAAA
Scn9a	NM_001290674.1	ACGGAGGTCTATGCCAAACT	ACCAACGCAAAAAGTAGCCA
Kcnn1	NM_001363407.1	CTTAACCGCGTCACCTTCAA	TATCGTGGTACCTCTCACACA
Kcnn2	NM_001312905.2	TTATCTTCGGCATGTTCGGC	AAGAATACAGCGACGCCTTG
Itgav	NM_008402.3	ATTGACGGGCCAATGAACTG	ATTCCACAGCCCAAAGTGTG
Itgb1	NM_010578.2	CTTATTGGCCTTGCCTTGCT	GATTTTCACCCGTGTCCCAC
Nlgn3	NM_172932.4	CCAACTTGGATATCGTCGCC	CATCTTCCGTGGGCACATAC
Nrxn1	NM_020252.3	TGACAGCAATTTGCCACTGA	CCTGTGTGTGTCTGGGGATA
Reln	NM_011261.2	TCGTCCTAGTAAGCACTCGC	GGAAGGGACACATTGTACGC
Cntnap2	NM_001004357.2	CATGGTGTACCAGACTTGCC	ATTGCTTACAGGGCTTTCCG
Arc	NM_018790.3	CCCCCAGCAGTGATTCATAC	GGTTTCATGCTGGCTTGTCT
Creb	NM_133828.2	ACAGGAGTCTGTGGATAGTGT	CCTGAGGCAGCTTGAACAAC
cFos	NM_010234	CAGAAGGGGCAAAGTAGAGC	TGATCTGTCTCCGCTTGGA
Dusp6	NM_026268.3	TTTCTTTCATAGATGAAGCCCGAG	GGGTCCTTTCGAAGTCAAGC
Egr1	NM_007913.5	GTCCTTTTCTGACATCGCTCTGA	CGAGTCGTTTGGCTGGGATA

Egr2	NM_010118.3	GCCAAGGCCGTAGACAAAAT	GTTGATCATGCCATCTCCCG
Npas4	NM_153553.5	ACCTGTCCCCAGAAGATCAC	CCCCTCCACTTCCATCTTCA
BDNF tot	NM_007540.4	ATTACCTGGATGCCGCAAA	TAATACTGTCACACACGCTCA
BDNF II	NM_001048139.1	GCCATCCACACGTGACAAAAC	TGCTGAATGGACTCTGCTCTC
BDNF IV	NM_001048141.1	CAGAGCAGCTGCCTTGATGTTT	CGCCTTCATGCAACCGAAGTAT
Fmr1	NM_008031.3	GGGTTGGACCTAACTCCTCT	TGATGAAACCACTAACACCCTC
Msra	NM_026322.4	GGTCAGCAGTCTATCCCACA	TGCTTTGAAAGAACCTTTTGGTATT
Braf	NM_139294.5	GGGCTGGTTTCCAAACAGAA	AATTCTCCATATCCCCCTGCT
Gm9758	NM_198666.4	AGTCAGAGGCTGGACATTGC	GCATCCTTCCTCCCCTCTCT
Gm11168	ENSMUST00000 178077	TCACATCCTAAAGTGTTGTGTATT	TGGCGAGAAACTGTAGGAAGA
Asmt	NM_001308488.2	CTTCACCGCCATCTACAGGTC	TGAAGGGCGAGAGGTCGAAG
Speer4cos	NR_001585.3	TAACACCGAAAACACCTCCTCA	TTTCTCTAACATCCTGCTGCACT
Actb	NM_007393.5	TTGCTGACAGGATGCAGAAG	AGTCCGCCTAGAAGCACTTG
Gapdh	NM_001289726.1	TGTGTCCGTCGTGGATCTGA	CCTGCTTCACCACCTTCTTGA
Hprt1	NM_013556.2	AAGCTTGCTGGTGAAAAGGA	TTGCGCTCATCTTAGGCTTT

ChIP-qPCR primers (figure S5)		Forward sequence (5' → 3')	Reverse sequence (5' → 3')
Itgb3 promoter		GAGTCCAGGAAGTGACCCAAA	AGGCTGAGTGTGATGGGTAAA
gRNA 2	Off-target #1	GGGGACACCCCTAGGAAAAT	GGCAAGATACGATGCCTTCC
	Off-target #2	GATTGGTTGTGCAATGATCGAG	CTGGAGACATCTGACGAGGA
	Off-target #3	TGCTGACTCCTCAAGGAACG	CCCCAAAGGTATCCTCGGTC
	Off-target #4	TGGGATTGTGTGGTGGGAAT	AACCCTCACCACTGTTCTCA
	Off-target #5	GTCTGTCCTGAGGTCTGGTG	TTCCACGGAAGTCCTAGCAT
	Off-target #6	TTCAGCACCGAACTCCGA	CCTAGGTGAGGAAGGACGG
	Off-target #7	CCTTTGAAGTGCCAACAGGA	GCTTCACCCATCCATGCC
	Off-target #8	AAAGGCTGCAGGAAAGAGTG	TTTCATGAGTGGGTGAGGGA
	Off-target #9	ATCATGAAGGCTGCGTGAAC	CCTGCTTACGTATGGGTGGA
	Off-target #10	GCCACTTGCATGGAGATACG	AGACGGTGGTGTGCTTCTAT
gRNA 3	Off-target #1	TTTGTAGAGCCCTGAGCCTG	AGCTCTCCAGGGATTAGCAC
	Off-target #2	AATAGGCCATGGAGTGGTCA	TTTCTGTGTGCGTCTGCATC
	Off-target #3	GACTCACTGAGACAAGCCCT	TGGTGTTCTCTTAGGCCAGT
	Off-target #4	ACTCCTCTGGGATGGAAGTC	CAATGAGGCAGGGAGAAGAC
	Off-target #5	GGGTGCTGTGTACAGGT	CAGGGCACACACACACC
	Off-target #6	TGCCAATAAGCAGCTGAACT	CAGACTGTGACTCTGTGGGA
	Off-target #7	CCCCAAAACCATATGAAAGGGG	GTGGCTCGACTTATGTTCCTTG
	Off-target #8	GATCAGCCCTCAGTGAGACA	TGAGCCCTAAGGAGACACAC
	Off-target #9	ATAGCTCCAGCTAAGGCTCG	ATCTGTCTTCACGTTGGCAG
	Off-target #10	ACGTCAAGTAATTGGGTAGGC	AAGCTCTTGTCATCACCGTAG
